# Six New Antimicrobial Metabolites from the Deep-Sea Sediment-Derived Fungus *Aspergillus fumigatus* SD-406

**DOI:** 10.3390/md20010004

**Published:** 2021-12-21

**Authors:** Li-Hong Yan, Xiao-Ming Li, Lu-Ping Chi, Xin Li, Bin-Gui Wang

**Affiliations:** 1 CAS and Shandong Province Key Laboratory of Experimental Marine Biology, Institute of Oceanology, Chinese Academy of Sciences, Nanhai Road 7, Qingdao 266071, China; yanlihong@qdio.ac.cn (L.-H.Y.); lixmqd@qdio.ac.cn (X.-M.L.); chiluping@qdio.ac.cn (L.-P.C.); 2 Laboratory of Marine Biology and Biotechnology, Qingdao National Laboratory for Marine Science and Technology, Wenhai Road 1, Qingdao 266237, China; 3 College of Marine Sciences, University of Chinese Academy of Sciences, Yuquan Road 19A, Beijing 100049, China; 4 Center for Ocean Mega-Science, Chinese Academy of Sciences, Nanhai Road 7, Qingdao 266071, China

**Keywords:** *Aspergillus fumigatus*, deep-sea sediment-derived fungus, alkaloids, triterpenoid, antimicrobial activity

## Abstract

Six new metabolites, including a pair of inseparable mixtures of secofumitremorgins A (**1a**) and B (**1b**), which differed in the configuration of the nitrogen atom, 29-hydroxyfumiquinazoline C (**6**), 10*R*-15-methylpseurotin A (**7**), 1,4,23-trihydroxy-hopane-22,30-diol (**10**), and sphingofungin I (**11**), together with six known compounds (**2**–**5** and **8**–**9**), were isolated and identified from the deep-sea sediment-derived fungus *Aspergillus fumigatus* SD-406. Their structures were determined by detailed spectroscopic analysis of NMR and MS data, chiral HPLC analysis of the acidic hydrolysate, X-ray crystallographic analysis, *J*-based configuration analysis, and quantum chemical calculations of ECD, OR, and NMR (with DP4+ probability analysis). Among the compounds, **1a**/**1b** represent a pair of novel scaffolds derived from indole diketopiperazine by cleavage of the amide bond following aromatization to give a pyridine ring. Compounds **1**, **4**, **6**, **7**, **10** and **11** showed inhibitory activities against pathogenic bacteria and plant pathogenic fungus, with MIC values ranging from 4 to 64 μg/mL.

## 1. Introduction

Deep-sea sediment has proven to be a treasure trove for structurally unique and biologically active secondary metabolites [1]. In the extreme environment of the deep-sea, microorganisms have gradually developed unique metabolic mechanisms out of adaption, thus possessing the great potential to produce natural products with significant biological properties, such as antimicrobial [2,3], cytotoxic [4], and antiviral [5] activities.

**Figure 1:**
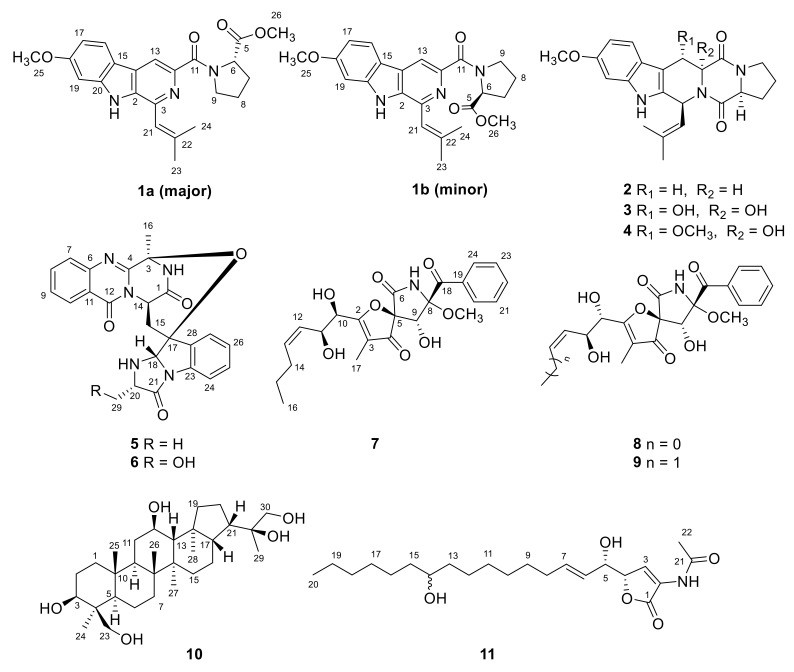
Structures of the isolated compounds **1**–**11**.

In the course of discovering bioactive metabolites from deep-sea-derived fungi [2,3,4], the fungal strain *Aspergillus fumigatus* SD-406, which was obtained from sediments collected from the deep-sea in the East China Sea, displayed abundant metabolites and antimicrobial activity in the preliminary screening, and thus attracted us to perform intensive chemical investigations on it. As a result, six new metabolites, including a mixture of a pair of inseparable isomers, secofumitremorgins A (**1a**) and B (**1b**), which differed in the configuration of the nitrogen atom, 29-hydroxyfumiquinazoline C (**6**), 10*R*-15-methylpseurotin A (**7**), 1,4,23-trihydroxy-hopane-22,30-diol (**10**), and sphingofungin I (**11**), together with six known compounds, fumitremorgin C (**2**) [6], 12,13-dihydroxyfumitremorgin C (**3**) [7], cyclotryprostatin B (**4**) [8], fumiquinazoline C (**5**) [9], 14-norpseurotin A (**8**) [10], and pseurotin A (**9**) [11] have been isolated and identified (Figure 1). Among these compounds, **1a**/**1b** represent a pair of novel scaffolds derived from indole diketopiperazine by cleavage of the amide bond following aromatization to give a pyridine ring. Details of the isolation and purification, structure elucidation, and biological evaluation of compounds **1**–**11** are described herein.

## 2. Results and Discussion

### 2.1. Structure Elucidation

The culture broth of fungus *Aspergillus fumigatus* SD-406 was extracted with EtOAc, and the crude extract was subjected to multiple chromatographic methods (a combination of column chromatography on Silica gel, Sephadex LH-20, and Lobar LiChroprep RP-18) to yield subfractions, which were further purified by preparative TLC and semipreparative HPLC to give compounds **1**–**11**.

**Figure 2:**
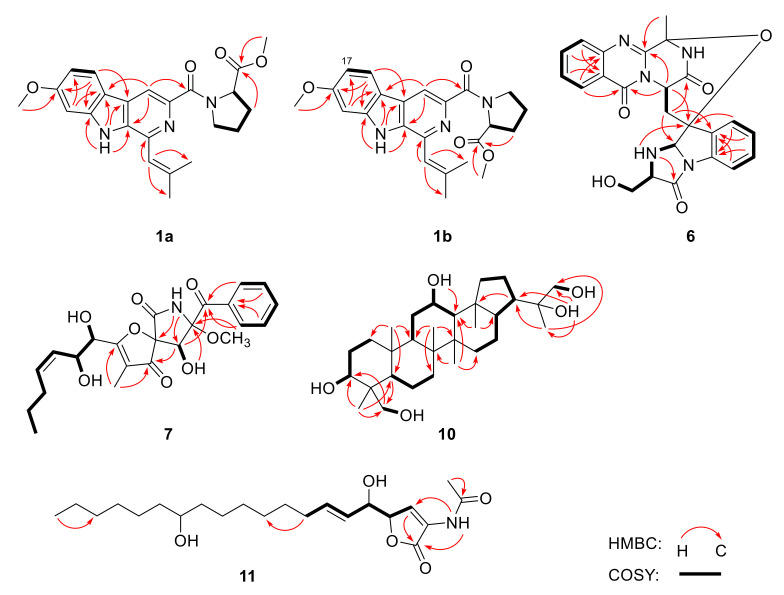
Key ^1^H-^1^H COSY (bold lines) and HMBC (red arrows) correlations of compounds **1a**, **1b**, **6**, **7**, **10** and **11**.

Compounds **1a**/**1b** were isolated as a mixture present in a ratio of 1:0.7 (major:minor). Attempts to separate two isomers by various types of chiral columns along with different elution ratios, unfortunately failed. The mixture (**1a**/**1b**) was found to have the same molecular formula as C_23_H_25_N_3_O_4_ on the basis of the HRESIMS *m/z* 406.1769 [M−H]^−^ (calculated for C_23_H_24_O_4_N_3_, 406.1772), requiring 13 degrees of unsaturation (Figure S8 in the supplementary material). The ^1^H- and ^13^C-NMR spectra (Figures S2 and S3 in the supplementary material) of the mixture presented two sets of similar data (Table 1), revealing that their planar structures both possessed 4 methyls (including 2 methoxyls), 3 aliphatic methylenes, 6 methines (including 5 sp^2^ hybridized and 1 connected to heteroatoms), and 10 quaternary carbons (including 2 carbonyls). Detailed analysis and comparison of the 1D and 2D NMR data (Figures S4–S7 in the supplementary material) indicated that the planar structures of **1a**/**1b** were similar to that of the known compound fumitremorgin C (**2**) [6]. However, signals for the methine groups at C-3 (*δ*_H/C_ 5.98/51.1) and C-12 (*δ*_H/C_ 4.16/56.9), methylene group at C-13 (*δ*_H/C_ 3.51/22.0), and amino carbonyl at C-5 (*δ*_C_ 165.9) in **2** were absent. Instead, signals for sp^2^ hybridized quaternary carbons at *δ*_C, major_ 138.5/*δ*_C, minor_ 138.2 and *δ*_C, major_ 141.9/*δ*_C, minor_ 141.8, for sp^2^ hybridized methines at *δ*_H/C, major_ 8.33/113.3/*δ*_H/C, minor_ 8.43/113.4, and for methyl ester groups at *δ*_C, major_ 172.6/*δ*_C, minor_ 172.9 and *δ*_H/C, major_ 3.67/51.6/*δ*_H/C, minor_ 3.46/51.4 were observed in the NMR spectra of **1a**/**1b** (Table 1), respectively. The above observation suggested that compounds **1a** and **1b** might be the derivatives of cleavage at the amide bond between N-4 and C-5 of fumitremorgin C (**2**). Meanwhile, aromatization occurred to generate a pyridine ring. This deduction was further verified by the key HMBC correlations from H-13 to C-2, C-11 and C-15, from H-26 and H-7 to C-5, and from H-21 to C-3 (Figure 2).

**Figure 3:**
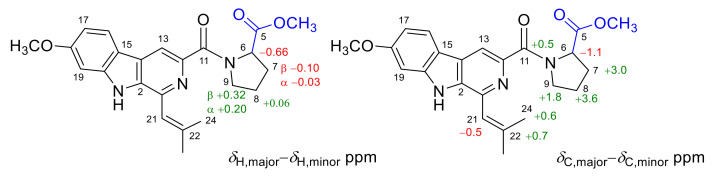
The main differences in chemical shifts between two groups of ^1^H NMR and ^13^C NMR data for compound **1**.

**Figure 4:**
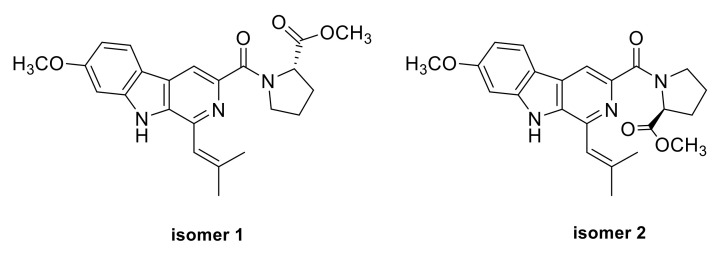
Structures of two isomers for DP4+ probability analysis of compound **1a** and **1b**.

The main differences between the two sets of NMR data were the chemical shifts around proline and isopentenyl moieties (Figure 3). Compared to the minor set, obvious higher chemical shifts for C-7, C-8, C-9, C-11, C-22, and C-24, and lower chemical shifts for C-6 and C-21 in the major set were observed (Figure 3). Based on the above deviation, a distinction in configuration of nitrogen atom N-10 between **1a** and **1b** was considered, and two candidate structures **isomers 1** and **2** were proposed (Figure 4). The C-N bond in the amino carbonyl cannot freely rotate due to the delocalization of the nitrogen atom’s lone electron pairs, thus resulting in the different orientation of the methyl ester group in isomers **1**/**2,** which explained well the differences in chemical shifts of proline and isopentenyl moieties. Besides, in the major set, the influence of π-systems of the aromatic rings led to obvious lower chemical shifts of H-6 and H-7α/β, whereas H-8α/β and H-9α/β remained unaffected (Figure 3). In the minor set, on the other hand, the chemical shifts for H-8α/β and H-9α/β were pushed to lower values by the aromatic rings, while H-6 and H-7α/β were unaffected (Figure 3). Thus, the major NMR data were assigned to **isomer 1** (**1a**) and the minor NMR data were assigned to **isomer 2** (**1b**).

To further confirm the assignment, a comparison of the observed NMR data with those of computed values for two possible isomers using DFT-NMR calculations with DP4+ probability analysis (see excel files in the supplementary material) was carried out [12]. As a result, the experimental NMR data of the major ^1^H and ^13^C NMR resonances corresponded to the computed NMR data for isomer **1** (100% probability, Table S5 in the supplementary material), while the calculated chemical shifts for isomer **2** were consistent with the minor ^1^H and ^13^C NMR resonances (100% probability, Table S6 in the supplementary material).

To determine the configuration of proline, a chiral HPLC analysis of the mixture’s acidic hydrolysate was carried out. The result of the HPLC analysis showed that the retention time of the acidic hydrolysate of mixture **1** was identical with that of _L_-Pro (Figure S1 in the supplementary material), indicating an _L_-proline, accordingly the 6*S* configuration of **1a**/**1b**. Thus, the structures of **1a** and **1b** were assigned as shown in Figure 1 and named secofumitremorgin A and secofumitremorgin B, respectively.

Compound **6** was obtained as a white solid. Its molecular formula was deduced as C_24_H_21_N_5_O_5_ on the basis of HRESIMS *m/z* 460.1603 [M+H]^+^ (calculated for C_24_H_22_N_5_O_5_, 460.1615), indicating 17 degrees of unsaturation (Figure S17 in the supplementary material). The ^1^H- and ^13^C-NMR spectra (Table 2, and Figures S11 and S12 in the supplementary material) displayed 1 methyl, 2 sp^3^ hybrid methylenes with 1 oxygenated, 8 aromatic methines and 3 sp^3^ hybrid methines connected to heteroatoms, and 10 quaternary carbons with 3 amino carbonyls. Detailed analysis of the 1D and 2D NMR data (Figures S13–S15 in the supplementary material) revealed that **6** showed close similarity to fumiquinazoline C (**5**) [9], except that the doublet methyl CH_3_-29 (*δ*_H_ 1.07/*δ*_C_ 18.8) of **5** was replaced by an oxygenated methylene (*δ*_H_ 3.41 H_a_, 3.21 H_b_/*δ*_C_ 61.8) in **6**. This was further supported by ^1^H-^1^H COSY correlations for the spin system of 19-NH/H-20/H-21/21-OH, to propose the structure of **6** (Figure 2).

**Figure 5:**
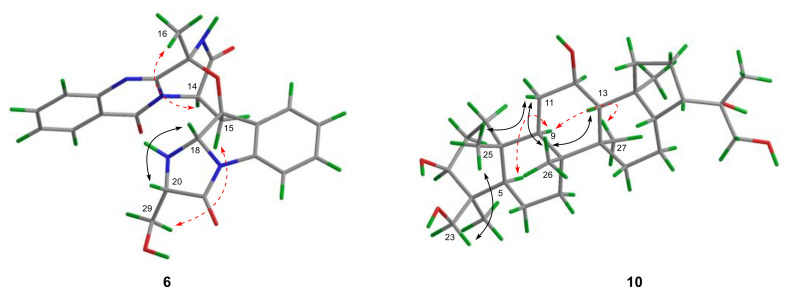
Key NOE correlations of compounds **6** and **10** (black solid lines: β-orientation; red dashed lines: α-orientation).

The relative configuration of **6** was deduced from analysis of NOESY data (Figure 5, and Figure S16 in the supplementary material). The NOESY correlations from H_3_-16 to H-14 indicated the cofacial orientation of them. Moreover, a NOE cross-peak from H-18 to H-20 suggested the same spatial orientation of them, while NOESY correlation from H-29 to H-15 placed these groups on the opposite face. The above observation also revealed the β-orientation of the ether bridge between C-3 and C-17 [13]. Thus, the relative configuration of **6** was identical to that of **5**. The absolute configuration of **6** was subsequently assigned as 3*R*,14*R*,17*S*,18*S*,20*S,* based on the same sign of the optical rotation to that of **5** ([α${]}_{25D}^{D}$ −193.7 (*c* = 0.17, CHCl_3_)) [13].

**Figure 6:**
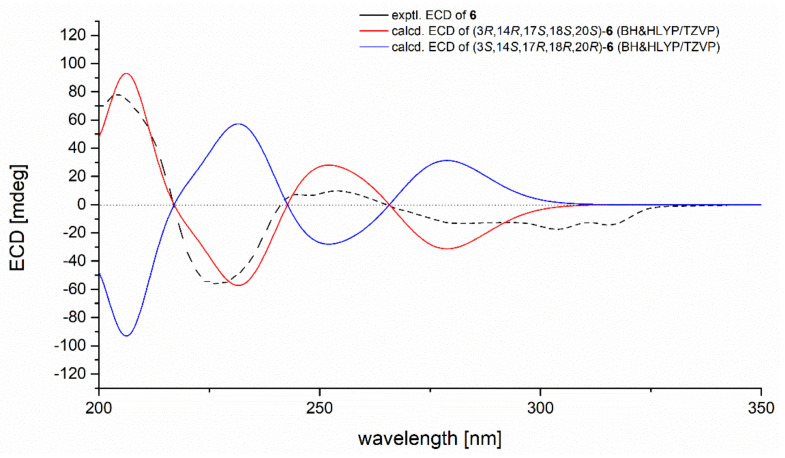
Experimental and calculated ECD spectra of compound **6**.

To further verify the absolute configuration of **6**, time-dependent, density functional (TDDFT)-ECD calculations at the BH&HLYP/TZVP level were performed. The calculated ECD spectrum for the (3*R*,14*R*,17*S*,18*S*,20*S*)-**6** matched well with that of the experimental curve (Figure S18 in the supplementary material), allowing the establishment of the absolute configuration of **6** as 3*R*,14*R*,17*S*,18*S*,20*S* (Figure 6). The trivial name 29-hydroxyfumiquinazoline C was assigned to **6**.

Compound **7** was isolated as a yellowish solid. The molecular formula was determined as C_23_H_27_NO_8_ by HRESIMS *m/z* 446.1799 [M+H]^+^ (calculated for C_23_H_28_NO_8_, 446.1809), which was 14 amu more than that of the previously-reported pseurotin A (**9**) [11], and accounted for 11 degrees of unsaturation (Figure S25 in the supplementary material). The ^1^H-, ^13^C-, and HSQC NMR spectra (Table 2, and Figures S19, S20, S22 and S23 in the supplementary material) revealed signals for three methyls (including one methoxyl), two aliphatic methylenes, ten methines (including three oxygenated and seven sp^2^ hybridized), two oxygenated, sp^3^ hybridized quaternary carbons, three sp^2^ hybridized quaternary carbons, two keto carbonyls (*δ*_C_ 196.7 and *δ*_C_ 196.3), and one amino carbonyl (*δ*_C_ 166.5). Detailed comparison of its NMR data with those of pseurotin A (**9**) showed close similarity in the planar structure, except for the additional appearance of an aliphatic methylene C-15 (*δ*_H_ 1.30/*δ*_C_ 22.2). Moreover, the significantly higher chemical shift for C-14 (*δ*_C_ 29.2) was also observed in **7**. The above observation indicated that **7** was the 15-methylation derivative of **9**. ^1^H-^1^H COSY correlations (Figure S21 in the supplementary material) for the spin system of H-10/H-11/H-12/H-13/H-14/H-15/H-16 further confirmed the planar structure of **7** (Figure 2).

**Figure 7:**
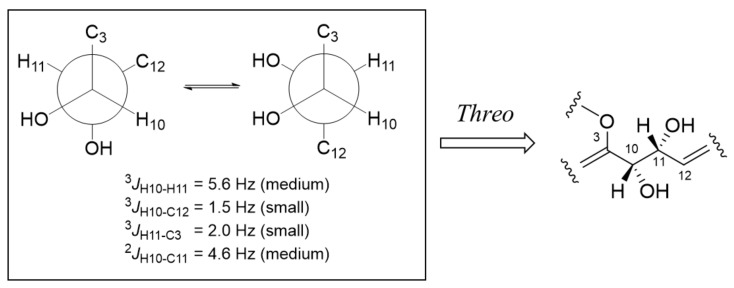
*J*-based configuration analysis of the chiral centers C-10 and C-11 of compound **7**.

To clarify the relative configurations of the chiral centers C-10 and C-11, *J*-based configuration analysis [14] was performed using ^3^*J*_H-H_, ^3^*J*_H-C_, and ^2^*J*_H-C_ coupling constants obtained from the ^1^H NMR and *J*-HMBC spectra (Figure S24 in the supplementary material). The medium ^3^*J*_H10-H11_ (5.6 Hz), the small ^3^*J*_H10-C12_ (1.5 Hz), the small ^3^*J*_H11-C3_ (2.0 Hz), and the medium ^2^*J*_H10-C11_ (4.6 Hz) matched with a pair of gauche/anti equilibrating rotamers in Figure 7, indicating the *syn/syn* orientation of H-10 and H-11. The absolute configurations of C-5 and C-8 were assigned based on electronic circular dichroism (ECD) spectrum of **7** (Figure S26 in the supplementary material), according to the previously reported rule [15]. As described in the previous reference, a negative Cotton effect at around 280 nm in the ECD spectrum (Figure S26 in the supplementary material) revealed *S* configuration of C-8, while the negative Cotton effect at around 230 nm and positive Cotton effect at 250 nm suggested the 5*S* configuration. The *cis* configurations of 8-OCH_3_ and 9-OH were deduced from the large coupling constant (*J* = 9.0 Hz) between H-9 and 9-OH [16,17]. Therefore, the absolute configuration of C-9 was assigned as 9*R*.

**Figure 8:**
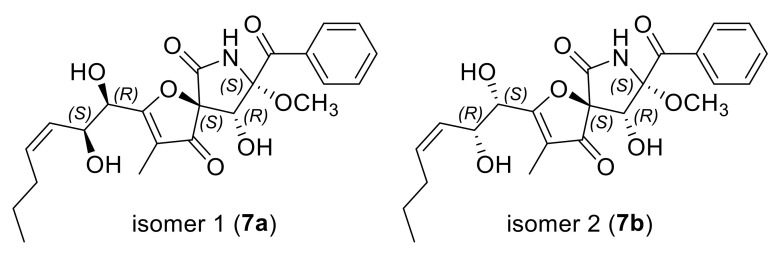
Structures of two possible isomers for DP4+ probability analysis of compound **7**.

Subsequently, NMR calculations with DP4+ probability analysis (see the excel files in the supplementary material) were carried out to correlate the stereochemical relationship between C-5/C-8/C-9 and C-10/C-11. The experimental ^1^H and ^13^C NMR data of **7** were compared with the calculated ^1^H and ^13^C NMR data of **7a** and **7b** (two possible isomers of **7**, Figure 8) and matched well with those calculated for the isomer **7a** (5*S*,8*S*,9*R*,10*R*,11*S*) with a DP4+ probability of 100% (Table S7 in the supplementary material). It could be seen that compound **7** possessed different absolute configurations with pseurotin A (**9**, 5*S*,8*S*,9*R*,10*S*,11*S*), which might lead to the opposite rotation of **7** ${\left[\alpha \right]}_{D}^{25}$ +6.0 (*c* 0.17, MeOH) to that of pseurotin A ${\left[\alpha \right]}_{D}^{20}$ −5 ± 1 (*c* 0.5, MeOH) [18]. Therefore, the chiral centers of compound **7** were tentatively assigned as 5*S*,8*S*,9*R*,10*R*,11*S*. Compound **7** was given the trivial name 10*R*-15-methylpseurotin A.

Compound **10**, initially obtained as a white amorphous powder, was found to have the molecular formula C_30_H_52_O_5_ on the basis of HRESIMS data *m/z* 527.3521 [M+^35^Cl]^−^ (calculated for C_30_H_52_O_5_Cl, 527.3509), suggesting five degrees of unsaturation (Figure S34 in the supplementary material). The 1D NMR spectra (Table 3, and Figures S28 and S29 in the supplementary material) suggested **10** was a pentacyclic triterpenoid containing six singlet methyls, eleven methylenes with two oxygenated, seven methines with two oxygenated, and six quaternary carbons with one oxygenated. Combined with 2D NMR (Figures S30–S32 in the supplementary material), **10** was suggested to be similar to the previously reported hopane-22,30-diol [19]. However, the signals for two methylenes at C-3 (*δ*_C_ 42.2) and C-12 (*δ*_C_ 24.2), and one methyl at C-23 (*δ*_C_ 33.4) present in the NMR spectra of hopane-22,30-diol were not detected in those of **10**, while resonances for two oxygenated methines at C-3 (*δ*_H_ 3.16/*δ*_C_ 78.6) and C-12 (*δ*_H_ 3.67/*δ*_C_ 67.9), and one oxygenated methylene at C-23 (*δ*_H_ 3.25, 3.80/*δ*_C_ 62.8) were observed in the NMR of **10**. These data indicated that C-3, C-12, and C-23 in hopane-22,30-diol were all substituted by hydroxy in **10**. This deduction was supported by key ^1^H-^1^H COSY correlations from 3-OH to H-3, from 12-OH to H-12, and from 23-OH to H-23 (Figure 2).

The relative configuration of **10** was partially assigned by NOESY spectrum (Figure 5, and Figure S33 in the supplementary material). NOE cross-peaks from H-5 to H-9 and from H-9 to H-27 indicated the cofacial orientation of these groups. Further, NOE correlations from H-23 to H-25, from H-13 to H-26, and from H-11α to H-25 and H-26 placed them on the same side. However, relative configurations of the rest of the chiral centers could not be assigned through analysis of NMR data.

**Figure 9:**
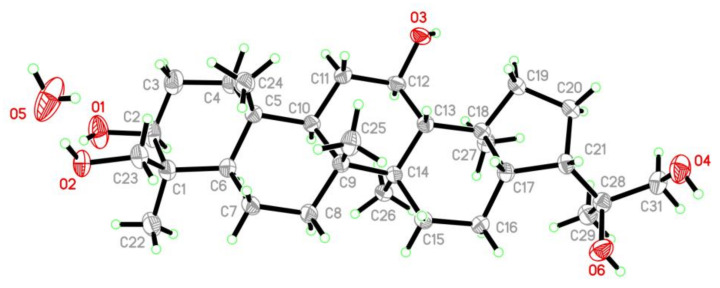
X-ray crystallographic structures of compound **10** [Flack parameter −0.04(5)].

After many attempts, the single crystal of **10** suitable for X-ray diffraction was obtained by slowly crystallizing in solvent MeOH at −4 °C (Figure 9), which not only confirmed the planar structure but also determined its relative configurations. Since the Flack parameter [−0.04(5)] was negative, calculation of optical rotation (OR) was performed at three different levels including BH&HLYP/TZVP, CAM-B3LYP/TZVP, and PBE0/TZVP. The calculated OR values (Table S1 in the supplementary material) for the (3*S*,4*S*,5*R*,8*R*,9*R*,10*R*,12*R*,13*R*,14*R*,17*S*,18*S*,21*S*,22*S*)-isomer of compound **10** matched well with that of the experimental OR [α${]}_{D}^{25}$ +30.0 (*c* 0.20, MeOH), allowing the establishment of the absolute configuration of **10** as 3*S*,4*S*,5*R*,8*R*,9*R*,10*R*,12*R*,13*R*,14*R*,17*S*,18*S*,21*S*,22*S*, which was identical with the result of X-ray diffraction. Thus, the trivial name 1,4,23-trihydroxy-hopane-22,30-diol was assigned to **10**.

Compound **11** was isolated as a white amorphous powder. The HRESIMS data *m/z* 394.2579 [M−H]^−^ (calculated for C_22_H_36_NO_5_, 394.2599) demonstrated its molecular formula to be C_22_H_37_NO_5_, accounting for five degrees of unsaturation (Figure S40 in the supplementary material. The 1D NMR (Table 3, and Figures S35 and S36 in the supplementary material) and HSQC spectra (Figure S38 in the supplementary material) displayed signals for a triplet methyl, 11 aliphatic methylenes, 3 oxygenated methines, 3 olefinic methines, 3 quaternary carbons with 2 carbonyls, and an amino group. The lipid side chain was deduced from the highly overlapped peak at *δ*_H_ 1.22–1.27, with an integral of 20 protons in the ^1^H-NMR spectrum. These NMR features were similar to those of sphingofungin H, which was isolated from *Aspergillus penicilliodes* Speg [20], except that the signals of an oxygenated methine C-3 (*δ*_H_ 4.57/*δ*_C_ 70.1) and a nitrogen-bearing methine C-2 (*δ*_H_ 4.70/*δ*_C_ 55.5) in sphingofungin H were replaced by an olefinic methine C-3 (*δ*_H_ 7.32/*δ*_C_ 127.4) and an olefinic quaternary carbon C-2 (*δ*_C_ 126.7) in **11**. These observations indicated that **11** was a dehydrated derivative of sphingofungin H at C-3 and C-2 with an additional double bond. This deduction was supported by key ^1^H-^1^H COSY correlation from H-3 to H-4 (Figure S37 in the supplementary material) and HMBC correlation from H-3 to C-1 (Figure S39 in the supplementary material).

The absolute configurations of C-4 and C-5 in **11** were established as 4*S*,5*S* based on the ECD spectrum (Figure S41 in the supplementary material), which showed a negative Cotton effect at ~210 nm and a positive Cotton effect at ~240 nm, which is similar to those of the previously reported acetyl derivative of malondungin [21]. The trivial name sphingofungin I was assigned to **11**.

The other known compounds, fumitremorgin C (**2**) [6], 12,13-dihydroxyfumitremorgin C (**3**) [7], cyclotryprostatin B (**4**) [8], fumiquinazoline C (**5**) [9], 14-norpseurotin A (**8**) [10], and pseurotin A (**9**) [11] were also isolated and identified. The structures of these compounds were determined by comparing their spectroscopic data with that reported in the literature.

### 2.2. Antimicrobial Activity

The isolated compounds (**1**–**11**) were evaluated for antimicrobial activity against several human-, aquatic-, and plant-pathogenic microbes (Table 4). Compound **4** showed activity against *Fusarium graminearum* Schw. with an MIC value of 64 μg/mL. Compound **1** exhibited activity against aquatic pathogenic bacterium *Vibrio alginolyticus* and *Edwardsiella tarda*, and the plant pathogenic fungi *F. graminearum* Schw. with MIC values of 32, 64, and 4 μg/mL, respectively. However, **2** and **3** showed no obvious activity against tested strains. These data indicated that the cleavage of diketopiperazine allowed for stronger activity against aquatic-, and plant-pathogens. Compared with compound **5**, the hydroxylation at C-29 of compound **6** afforded its weak inhibitory effect against the plant pathogenic fungi *F. oxysporum* with an MIC value of 64 μg/mL. Compared with compounds **8** and **9**, compound **7** with the extension of side chain exhibited moderate activity against the plant-pathogenic fungi *F. graminearum* Schw., with an MIC value of 16 μg/mL. The triterpenoid **10** displayed moderate activity against *V. alginolyticus* and *F. graminearum* Schw., with MIC values of 16 and 32 μg/mL, respectively. In addition, the five-membered lactone **11** showed excellent antibacterial activity against *Pseudomonas aeruginosa*, *V. alginolyticus*, and *E. tarda* with MIC values of 8, 8, and 8 μg/mL, respectively.

## 3. Experimental Section

### 3.1. General Experimental Procedures

Detailed information for apparatus, reagents, solvents, and materials are same as described in our previous publication [3].

### 3.2. Fungal Material

The fungus *Aspergillus fumigatus* SD-406 was isolated from the deep-sea sediment of the East China Sea (121°20.2′ E, 26°45.5′ N), collected in September 2017. The fungal strain was identified as *Aspergillus fumigatus* according to the ITS (internal transcript spacer) region sequence, which is the same (100%) as that of *Aspergillus fumigatus* (accession No. MT635279). The sequence data of SD-406 have been deposited in GenBank with the accession No. OL662987. The strain is preserved at the Key Laboratory of Experimental Marine Biology, Institute of Oceanology, Chinese Academy of Sciences (IOCAS).

### 3.3. Fermentation, Extraction and Isolation

The fungal strain *Aspergillus fumigatus* SD-406 was cultivated on potato dextrose agar medium at 28 °C for 7 days, which was then transferred into 100 × 1 L Erlenmeyer flasks with rice solid medium (each flask containing 70 g rice, peptone from animal 0.3 g, yeast extract 0.5 g, corn steep liquor 0.2 g, monosodium glutamate 0.1 g and naturally sourced seawater) and incubated at room temperature for 30 days. Then, the solid fermented substrate was extracted three times with EtOAc and the combined extracts were concentrated under reduced pressure to give a dark brown crude extract (76 g).

The EtOAc extract was subjected to Silica gel VLC (vacuum liquid chromatography) and fractionated using solvent mixtures of increasing polarity consisting of petroleum ether (PE) and EtOAc (50:1 to 1:1), and subsequently with CH_2_Cl_2_-MeOH (50:1 to 1:1) to yield 11 fractions (Frs. 1–11). Purification of fraction 6 (15.6 g), performed with CC (Column Chromatography) over Lobar LiChroprep RP-18 with a MeOH-H_2_O gradient (from 1:9 to 10:0), yielded 10 subfractions (fractions 6.1–6.10). Fraction 6.2 (565.0 mg) was subjected to CC on Si gel (CH_2_Cl_2_-MeOH, 70:1 to 10:1) and then purified by semipreparative HPLC (50% MeOH-H_2_O, 12 mL/min) to give **8** (25.3 mg) and **9** (23.8 mg). Fraction 6.4 (1.0 g) was further purified by CC on Si gel eluting with a CH_2_Cl_2_/MeOH gradient (from 150:1 to 50:1) to yield seven subfractions (Frs. 6.4.1–6.4.7). Fraction 6.4.3 was further purified by semipreparative HPLC (65% MeOH-H_2_O, 12 mL/min) to give **5** (27.4 mg). Fraction 6.4.4 was further purified by preparative TLC, as well as Sephadex LH-20 (MeOH), to yield **4** (4.2 mg). Fraction 6.4.7 was purified by semipreparative HPLC (60% MeOH−H_2_O, 10 mL/min) to give **7** (13.1 mg). Fraction 6.5 (1.2 g) was subjected to CC on Si gel (CH_2_Cl_2_-MeOH, 200:1 to 50:1) and subsequent recrystallization afforded **3** (25.7 mg). Fraction 7 (5.4 g) was further fractionated by CC over Lobar LiChroprep RP-18 with a MeOH-H_2_O gradient (from 1:9 to 10:0) to yield 10 subfractions (Frs. 7.1–7.10). Fraction 7.4 was separated by CC on Si gel and Sephadex LH-20 (MeOH) to obtain **6** (9.8 mg). Fraction 7.5 was purified by CC on Si gel eluting with CH_2_Cl_2_-MeOH 100/1 to give **2** (26.6 mg). Fraction 8 (7.2 g) was fractionated by CC over Lobar LiChroprep RP-18 with a MeOH-H_2_O gradient (from 1:9 to 10:0) to yield 10 subfractions (Frs. 8.1−8.10). Fraction 8.7 was further separated by Sephadex LH-20 (MeOH) and preparative TLC to give **11** (3.5 mg). Fraction 9 (4.0 g) was also purified by CC over Lobar LiChroprep RP-18 with a MeOH-H_2_O gradient (from 1:9 to 10:0) to afford 10 subfractions (Frs. 9.1–9.10). Recrystallization of fraction 9.5 yielded **10** (5.6 mg). Fraction 9.6 (80.0 mg) was further purified by CC on Si gel eluting with CH_2_Cl_2_-MeOH 80-1 and then by semipreparative HPLC (80% MeOH−H_2_O, 3 mL/min) to give **1** (6.0 mg).

Secofumitremorgin A/B (**1a**/**1b**): colorless amorphous powder; [α${]}_{D}^{25}$ −30.0 (*c* = 0.3, MeOH); UV (MeOH) *λ_max_* (log *ε*) 215 (4.28) nm, 281 (4.48) nm, 305 (4.08) nm, 340 (3.77) nm; ^1^H and ^13^C NMR data, Table 1; HRESIMS *m/z* 406.1769 [M-H]^−^ (calculated for C_23_H_24_O_4_N_3_, 406.1772).

29-Hydroxyfumiquinazoline C (**6**): white solid; [α${]}_{D}^{25}$ −276.0 (*c* = 0.17, MeOH); UV (MeOH) *λ_max_* (log *ε*) 224 (3.73) nm, 280 (3.19) nm, 304 (2.87) nm, 316 (2.79) nm; ECD (0.44 mm, MeOH) *λ_max_* (Δ*ε*) 226 (−39.00), 254 (+6.86), 304 (−12.10) nm; ^1^H and ^13^C NMR data, Table 2; HRESIMS *m/z* 460.1603 [M+H]^+^ (calculated for C_24_H_22_N_5_O_5_, 460.1615).

10*R*-15-Methylpseurotin A (**7**): yellowish solid; [α${]}_{D}^{25}$ +6.0 (*c* = 0.17, MeOH); UV (MeOH) *λ_max_* (log *ε*) 252 (3.25) nm, 280 (3.07) nm; ECD (0.45 mm, MeOH) *λ_max_* (Δ*ε*) 233 (−3.83), 252 (+6.24), 277 (−14.66), 312 (+4.24), nm; ^1^H and ^13^C NMR data, Table 2; HRESIMS *m/z* 446.1799 [M+H]^+^ (calculated for C_23_H_28_NO_8_, 446.1809).

1,4,23-Trihydroxy-hopane-22,30-diol (**10**): white amorphous powder; mp 323–326 °C; [α${]}_{D}^{25}$ +30.0 (*c* = 0.20, MeOH); ^1^H and ^13^C NMR data, Table 3; HRESIMS *m/z* 527.3521 [M+^35^Cl]^−^ (calculated for C_30_H_52_O_5_Cl, 527.3509).

Sphingofungin I (**11**): white amorphous powder; [α${]}_{D}^{25}$ +30.4 (*c* = 0.23, MeOH); UV (MeOH) *λ_max_* (log *ε*) 246 (3.07) nm, 331 (2.18) nm; ECD (0.51 mm, MeOH) *λ_max_* (Δ*ε*) 210 (−3.98), 246 (+4.52) nm; ^1^H and ^13^C NMR data, Table 3; HRESIMS *m/z* 394.2579 [M−H]^−^ (calculated for C_22_H_36_NO_5_, 394.2599).

### 3.4. Acidic Hydrolysis of Compound **1**

Compound **1** (1 mg) was dissolved in 10 mL of 6 N HCl and heated in a sealed tube at 110 °C for 24 h. The solutions were then evaporated to dryness under reduced pressure. Each sample, including the standard amino acids l-Pro and d-Pro, were dissolved in 1 mL of eluting solvent (2 mM CuSO_4_·5H_2_O in 100 mL of H_2_O). Chiral HPLC analysis, both alone and by co-injection with standards, was carried out using a Phenomenex-Chirex-3126 column (150 mm × 4.60 mm, 5 μm; flow rate 1.0 mL/min at 25 °C; detection at 254 nm).

### 3.5. X-ray Crystallographic Analysis of Compound **10**

Crystallographic data were collected on an Agilent Xcalibur Eos Gemini CCD plate diffractometer utilizing graphite–monochromatic Cu-Kα radiation (λ = 1.54178 Å) at 293 (2) K [22]. The data were corrected for absorption using the program SADABS [23]. The structures were solved by direct methods with the SHELXTL software package [24]. All nonhydrogen atoms were refined anisotropically. The H atoms connected to C atoms were calculated theoretically, and those to O atoms were assigned by difference Fourier maps [25]. The structures were optimized by full-matrix, least-squares techniques.

*Crystal data for compound **10**:* C_30_H_52_O_5_·H_2_O, F.W. = 510.73, monoclinic space group *P*2(1), unit cell dimensions *a* = 6.5814 (7) Å, *b* = 29.791 (3) Å, *c* = 7.1418 (7) Å, *V* = 1331.1 (2) Å, α = γ = 90°, β = 108.085 (8)°, *Z* = 2, *d*_calcd_ = 1.274 mg/m^3^, crystal dimensions 0.60 × 0.15× 0.12 mm, *μ* = 0.685 mm^−1^, *F*(000) = 564. The 4871 measurements yielded 4742 independent reflections after equivalent data were averaged. The final refinement gave *R*_1_ = 0.0292 and w*R*_2_ = 0.0771 [*I* > 2*σ*(*I*)]. The Flack parameter was −0.04(5).

### 3.6. ECD Calculation of Compound **6** and OR Calculation of Compound **10**

Conformational searches were carried out via molecular mechanics with the MM+ method in HyperChem 8.0 software, and the geometries were optimized at the gas-phase B3LYP/6-31G(d) level in Gaussian09 software (Version D.01; Gaussian, Inc.: Wallingford, CT, USA) [26] to afford the energy-minimized conformers. Then, the optimized conformers were subjected to the calculations of ECD spectra using the TD-DFT at BH&HLYP/TZVP levels, and solvent effects of the MeOH solution were evaluated at the same DFT level using the SCRF/PCM method.

Optical rotations of the optimized conformers were calculated using the TDDFT method at BH&HLYP/TZVP, CAM-B3LYP/TZVP and PBE0/TZVP levels in methanol (λ = 589.4 nm). The calculated optical rotations were later obtained according to the Boltzmann weighting of each conformer.

### 3.7. Computational NMR Chemical Shift and DP4+ Analyses

All the theoretical calculations were conducted in Gaussian09 program package [26]. Conformational searches for possible isomers based on molecular mechanics were conducted with the MM+ method in HyperChem 8.0 software. The corresponding stable conformers, whose Boltzmann distributions were higher than 2%, were further optimized at the B3LYP/6-31G(d) PCM level in DMSO (Tables S2–S4, and Figures S9, S10 and S27 in the supplementary material). Then, all optimized conformers were subjected to the DFT method at the mPW1PW91/6-31+G(d) PCM level in DMSO to acquire calculated shielding tensors. Then, the calculated shielding tensors were averaged based on Boltzmann distribution theory. Finally, the DP4+ analysis of the calculated shielding tensors and experimental chemical shifts was applied, using the Excel template provided by the original authors [12].

### 3.8. Bioassay

The antimicrobial activities against human pathogenic bacteria (*Pseudomonas aeruginosa*), aquatic pathogens (*Edwardsiella tarda* and *Vibrio alginolyticus*), and plant pathogens (*Fusarium oxysporum* and *Fusarim graminearum* Schw.) were determined by a serial dilution technique, using 96-well microtiter plates with minor modifications as per our previous report [27]. The human, aquatic, and plant pathogenic strains were offered by the Institute of Oceanology, Chinese Academy of Sciences. Chloramphenicol was used as a positive control for the bacteria, and amphotericin B was used as a positive control for the fungi.

## 4. Conclusions

In summary, 11 compounds, including 6 new compounds (**1a**, **1b**, **6**, **7**, **10** and **11**), were obtained from the deep-sea, sediment-derived fungus *Aspergillus fumigatus* SD-406. Among them, secofumitremorgins A/B featured an unusual seco-diketopiperazine scaffold and the formation of a pyridine moiety. The stereoconfigurations of isolated compounds were determined by chiral HPLC analysis of the acidic hydrolysate, X-ray crystallographic analysis, J-based configuration analysis, and quantum chemical calculations of ECD, OR, and NMR (with DP4+ probability analysis). Compounds **1**, **4**, **6**, **7**, **10** and **11** showed inhibitory activities against pathogenic bacteria and plant-pathogenic fungus, with MIC values ranging from 4 to 64 μg/mL, possessing the potential to be developed as antibiotic lead compounds.

## Figures and Tables

**Table 1:** ^1^H and ^13^C NMR data of compounds **1a** and **1b** (measured in DMSO-*d_6_*).

No.	1a (Major)		1b (Minor)	
	*δ*_C_, Type ^a^	*δ*_H_ (*J* in Hz) ^b^	*δ*_C_, Type ^a^	*δ*_H_ (*J* in Hz) ^b^
1-NH		11.75, s		11.55, s
2	134.6, C		134.4, C	
3	138.5, C		138.2, C	
5	172.6, C		172.9, C	
6	59.6, CH	4.55, dd, (8.5, 4.6)	60.7, CH	5.21, dd, (8.6, 3.5)
7	28.3, CH_2_	β 1.87, m; α 2.25, m	31.3, CH_2_	β 1.97, m; α 2.28, m
8	25.2, CH_2_	1.89, m	21.6, CH_2_	1.83, m
9	49.6, CH_2_	β 4.01, m; α 3.89, m	47.8, CH_2_	3.69, m
11	166.7, C		166.2, C	
12	141.9, C		141.8, C	
13	113.3, CH	8.33, s	113.4, CH	8.43, s
14	128.1, C		128.3, C	
15	114.9, C		114.9, C	
16	122.7, CH	8.17, d, (8.7)	122.7, CH	8.17, d, (8.6)
17	109.6, CH	6.88, dd, (8.7, 2.2)	109.6, CH	6.87, dd, (8.6, 2.2)
18	160.4, C		160.4, C	
19	94.8, CH	7.04, d, (2.2)	94.8, CH	7.04, d, (2.2)
20	142.3, C		142.5, C	
21	119.5, CH	6.79, s	120.0, CH	6.59, s
22	141.1, C		140.4, C	
23	20.2, CH_3_	2.19, s	20.0, CH_3_	1.91, s
24	27.1, CH_3_	2.06, s	26.5, CH_3_	2.03, s
25	55.3, CH_3_	3.88, s	55.3, CH_3_	3.87, s
26	51.6, CH_3_	3.67, s	51.4, CH_3_	3.46, s

^a^ Measured at 125 MHz; ^b^ Measured at 500 MHz.

**Table 2:** ^1^H and ^13^C NMR data for compounds **6** and **7** (measured in DMSO-*d_6_*).

No.	6		No.	7	
	δ_C_, Type ^a^	δ_H_ (J in Hz) ^b^		δ_C_, Type ^a^	δ_H_ (J in Hz) ^b^
1	169.9, C		2	186.9, C	
2-NH		9.97, s	3	111.5, C	
3	83.7, C		4	196.7, C	
4	150.3, C		5	91.1, C	
6	146.4, C		6	166.5, C	
7	128.2, CH	7.82, dd, (8.3, 1.2)	7-NH		9.94, s
8	134.7, CH	7.91, td, (8.3, 1.6)	8	92.4, C	
9	128.0, CH	7.65, td, (8.3, 1.2)	8-OCH_3_	51.6, CH_3_	3.25, s
10	126.2, CH	8.21, dd, (8.3, 1.6)	9	74.9, CH	4.40, d, (9.4)
11	120.9, C		9-OH		6.22, s, (9.4)
12	160.0, C		10	71.9, CH	4,34, t, (5.3)
14	51.3, CH	5.34, dd, (6.7, 1.2)	11	68.3, CH	4.45, m
15	30.7, CH_2_	β 3.31, m ; α 1.90, m	12	129.8, CH	5.42, dd, (8.7, 11.0)
16	23.9, CH_3_	1.90, s	13	131.9, CH	5.43, dd, (6.9, 11.0)
17	86.4, C		14	29.2, CH_2_	1.99, m
18	87.2, CH	5.18, d, (8.6)	15	22.2, CH_2_	1.30, m
19-NH		2.32, t, (8.6)	16	13.5, CH_3_	0.83, t, (7.4)
20	64.3, CH	3.64, m	17	5.6, CH_3_	1.64, s
21	169.6, C		18	196.3, C	
23	135.7, C		19	133.4, C	
24	114.7, CH	7.32, dd, (7.3, 1.2)	20	130.2, CH	8.25, d, (7.8)
25	129.6, CH	7.35, td, (7.3, 1.2)	21	128.3, CH	7.53, t, (7.8)
26	125.8, CH	7.24, td, (7.3, 1.2)	22	133.7, CH	7.67, t, (7.8)
27	124.8, CH	7.31, dd. (7.3, 1.2)	23	128.3, CH	7.53, t, (7.8)
28	138.8, C		24	130.2, CH	8.25, d, (7.8)
29	61.8, CH_2_	β 3.21, m; α 3.41, m			
29-OH		4.16, t, (5.5)			

^a^ Measured at 125 MHz; ^b^ measured at 500 MHz.

**Table 3:** ^1^H and ^13^C NMR data for compounds **10** and **11** (measured in DMSO-*d_6_*).

No.	10		No.	11	
	δ_C_, Type ^a^	δ_H_ (J in Hz) ^b^		δ_C_, Type ^a^	δ_H_ (J in Hz) ^b^
1	38.1, CH_2_	α 0.89, m; β 1.61, m	NH		10.03, s
2	27.3, CH_2_	α 1.58, m; β 1.58, m	1	168.9, C	
3	78.6, CH	3.16, m	2	126.7, C	
3-OH		4.96, d, (4.8)	3	127.4, CH	7.32, d, (1.9)
4	42.1, C		4	83.5, CH	4.98, m
5	55.3, CH	0.69, m	5	71.5, CH	4.15, d, (5.2)
6	18.6, CH_2_	α 1.56, m; β 1.45, m	6	128.5, CH	5.32, m
7	33.0, CH_2_	α 1.28, m; β 1.15, m	7	132.9, CH	5.65, m
8	40.8, C		8	31.3, CH_2_	1.98, m
9	48.5, CH	1.20, m	9	29.0, CH_2_	1.20–1.29, m
10	36.2, C		10	28.8, CH_2_	1.20–1.29, m
11	32.2, CH_2_	α 1.67, m; β 1.21, m	11	28.4, CH_2_	1.20–1.29, m
12	67.9, CH	3.67, m	12	25.1, CH_2_	1.20–1.29, m
12-OH		3.82, overlap	13	37.1, CH_2_	1.20–1.29, m
13	55.0, CH	1.20, m	14	69.5, CH	3.34, m
14	42.2, C		15	37.1, CH_2_	1.20–1.29, m
15	34.7, CH_2_	α 1.26, m; β 1.08, m	16	25.1, CH_2_	1.20–1.29, m
16	20.9, CH_2_	α 1.53, m; β 1.94, m	17	28.4, CH_2_	1.20–1.29, m
17	54.1, CH	1.29, m	18	31.5, CH_2_	1.20–1.29, m
18	42.9, C		19	22.0, CH_2_	1.20–1.29, m
19	43.9, CH_2_	α 1.93, m; β 1.03, m	20	13.8, CH_3_	1.20–1.29, m
20	25.6, CH_2_	α 1.38, m; β 1.52, m	21	169.5, C	
21	43.2, CH	2.13, m	22	22.9, CH_3_	2.06, s
22	74.4, C				
22-OH		3.59, s			
23	62.8, CH_2_	α 3.80, overlap; β 3.25, dd, (10.9, 7.7)			
23-OH		4.06, dd, (7.7, 3.1)			
24	22.8, CH_3_	1.05, s			
25	15.8, CH_3_	0.76, s			
26	16.3, CH_3_	0.88, s			
27	17.6, CH_3_	0.86, s			
28	16.0, CH_3_	0.81, s			
29	23.1, CH_3_	0.95, s			
30	69.2, CH_2_	3.17, m			
30-OH		4.33, t, (5.7)			

^a^ Measured at 125 MHz; ^b^ measured at 500 MHz.

**Table 4:** Antimicrobial activities of compounds ^a^ **1**, **4**, **6**, **7**, **10** and **11** (MIC, μg/mL).

Strain	Compound						
	1	4	6	7	10	11	Positive Control
*Pseudomonas aeruginosa* ^b^	- ^d^	-	-	-	-	8	4
*Vibrio alginolyticus* ^b^	32	-	-	-	16	8	1
*Edwardsiella tarda* ^b^	64	-	-	-	-	8	1
*Fusarium oxysporum* ^c^	-	-	64	-	-	-	2
*Fusarium graminearum* Schw. ^c^	4	64	-	16	32	-	0.5

^a^ Compounds **2**, **3**, **5**, **8**, and **9** showed no activity against tested strains. ^b^ Chloramphenicol as positive control. ^c^ Amphotericin B as positive control. ^d^ (-) = MIC > 64 μg/mL.

## Supplementary Materials

The following supporting information can be downloaded at https://www.mdpi.com/article/10.3390/md20010004/s1, Table S1: OR calculations of compound **10**; Tables S2–S6: DP4+ probability analysis data of compounds **1a**, **1b**, and **7**; Figure S1: Chiral HPLC analysis of the acidic hydrolysate of compounds **1a**/**1b**; Figures S2–S8, S11–26 and S28–S41: The HRESIMS, 1D and 2D NMR spectra, ECDs of compounds **1**, **6**, **7**, **10** and **11**; Figures S9, S10 and S27: Optimized geometries of predominant conformers for compounds **1a**, **1b** and **7**, respectively. The excel files for DP4+ probability analysis for compounds **1a**, **1b** and **7** were also provided.
