# Supplementary material for: Six New Antimicrobial Metabolites from the Deep-Sea Sediment-Derived Fungus Aspergillus fumigatus SD-406
Source: Mar Drugs. 2021 Dec 21;20(1):4. doi: 10.3390/md20010004 (PMC8780737; doi:10.3390/md20010004)

Supplementary Material

Six New Antimicrobial Metabolites from the Deep-sea Sediment Derived Fungus *Aspergillus fumigatus* SD-406

Li-Hong Yan [1,2,3], Xiao-Ming Li [1,2,4], Lu-Ping Chi[1,2,3], Xin Li [1,2,4,*] and Bin-Gui Wang [1,2,3,4,*]

Contents

Table S1. OR calculations of compound **10** (at 589.4 nm);	S1
Table S2. The Cartesian coordinates of the lowest energy conformers (Conf. 1-8) for **isomer 1** of compounds **1a/1b;**	S1-6
Table S3. The Cartesian coordinates of the lowest energy conformers (Conf. 1-9) for **isomer 2**f compounds **1a/1b;**	S6-13
Table S4 The Cartesian coordinates of the lowest energy conformers (Conf. 1-6) for **isomer 7a** of compound **7;**	S13-17
Table S5 DP4+ probability analysis of compound **1a**;	S18-19
Table S6 DP4+ probability analysis of compound **1b**;	S20-21
Table S7 DP4+ probability analysis of compound **7**;	S22-23
Figure S1. Chiral HPLC analysis of the acidic hydrolysate of compounds **1a/1b**;	S24
Figure S2. ^{1}H-NMR (500 MHz, DMSO-d_6) spectrum of compounds **1a/1b**;	S24
Figure S3. ^{13}C NMR (125 MHz, DMSO-d_6) and DEPT spectra of compounds **1a/1b**;	S25
Figure S4. ^{1}H–^{1}H COSY spectrum of compounds **1a/1b**;	S25
Figure S5. HSQC spectrum of compounds **1a/1b**;	S26
Figure S6. HMBC spectrum of compounds **1a/1b**;	S26
Figure S7. NOESY spectrum of compounds **1a/1b**;	S27
Figure S8. HR-ESI-MS spectrum of compounds **1a/1b**;	S27
Figure S9 Optimized geometries of predominant conformers (weighting factors) for **isomer 1** at the B3LYP/6-311g(d,p) level.	S28
Figure S10 Optimized geometries of predominant conformers (weighting factors) for **isomer 2** at the B3LYP/6-311g(d,p) level.	S28
Figure S11. ^{1}H-NMR (500 MHz, DMSO-d_6) spectrum of compound **6**;	S29
Figure S12. ^{13}C NMR (125 MHz, DMSO-d_6) and DEPT spectra of compound **6**;	S29
Figure S13. ^{1}H–^{1}H COSY spectrum of compound **6**;	S30
Figure S14. HSQC spectrum of compound **6**;	S30
Figure S15. HMBC spectrum of compound **6**;	S31
Figure S16. NOESY spectrum of compound **6**;	S31
Figure S17. HR-ESI-MS spectrum of compound **6**;	S32
Figure S18. ECD spectrum of compound **6**;	S32
Figure S19. ^{1}H-NMR (500 MHz, DMSO-d_6) spectrum of compound **7**;	S33
Figure S20. ^{13}C NMR (125 MHz, DMSO-d_6) and DEPT spectra of compound **7**;	S33
Figure S21. ^{1}H–^{1}H COSY spectrum of compound **7**;	S34
Figure S22. HSQC spectrum of compound **7**;	S34
Figure S23. HMBC spectrum of compound **7**;	S35
Figure S24. *J*-HMBC spectrum of compound **7**;	S35
Figure S25. HR-ESI-MS spectrum of compound **7**;	S36
Figure S26. ECD spectrum of compound **7**;	S36
Figure S27 Optimized geometries of predominant conformers (weighting factors) for **isomers 7a and 7b** at the B3LYP/6-311g(d,p) level.	S37
Figure S28. ^{1}H-NMR (500 MHz, DMSO-d_6) spectrum of compound **10**;	S38
Figure S29. ^{13}C NMR (125 MHz, DMSO-d_6) and DEPT spectra of compound **10**;	S38

Figure S30. ^{1}H–^{1}H COSY spectrum of compound **10**; S39
Figure S31. HSQC spectrum of compound **10**; S39
Figure S32. HMBC spectrum of compound **10**; S40
Figure S33. NOESY spectrum of compound **10**; S40
Figure S34. HR-ESI-MS spectrum of compound **10**; S41
Figure S35. ^{1}H-NMR (500 MHz, DMSO-d_6) spectrum of compound **11**; S41
Figure S36. ^{13}C NMR (125 MHz, DMSO-d_6) and DEPT spectra of compound **11**; S42
Figure S37. ^{1}H–^{1}H COSY spectrum of compound **11**; S42
Figure S38. HSQC spectrum of compound **11**; S43
Figure S39. HMBC spectrum of compound **11**; S43
Figure S40. HR-ESI-MS spectrum of compound **11**; S44
Figure S41. ECD spectrum of compound **11**; S44

Table S1. OR calculations of compound **10** (at 589.4 nm).

Isomers	BH&HLYP/TZVP	CAM-3LYP/TZVP	PBE0/TZVP
3*S*,4*S*,5*R*,8*R*,9*R*,10*R*,12*R*,13*R*,14*R*, 17*S*,18*S*,21*S*,22*S*	+44.8	+46.7	+50.9
3*R*,4*R*,5*S*,8*S*,9*S*,10*S*,12*S*,13*S*,14*S*, 17*R*,18*R*,21*R*,22*R*	-44.8	-46.7	-50.9
experimental OR values	$[\alpha]_{D}^{25}$ +30.0 (c =0.20, MeOH)		

Table S2. The Cartesian coordinates of the lowest energy conformers (Conf. 1-8) for **isomer 1** of compounds **1a/1b**.

Conf. 1-1	X axis(Å)	Y axis(Å)	Z axis(Å)	Conf. 1-2	X axis(Å)	Y axis(Å)	Z axis(Å)
C	-1.676403	-0.373761	-0.131373	C	-1.596467	-0.527839	-0.223271
C	-1.790929	1.02724	0.048633	C	-1.746349	0.855987	0.044869
C	-3.019095	-0.915174	-0.065702	C	-2.920819	-1.109862	-0.173989
C	-3.892887	0.186574	0.143911	C	-3.818878	-0.053807	0.114421
N	-3.129875	1.34415	0.224022	N	-3.091382	1.119991	0.257228
C	-3.564678	-2.197763	-0.1699	C	-3.43343	-2.405414	-0.353144
C	-4.944276	-2.374357	-0.070913	C	-4.798218	-2.618884	-0.246606
C	-5.791502	-1.262375	0.134067	C	-5.678408	-1.547028	0.039269
C	-5.2731	0.033123	0.245013	C	-5.202255	-0.246475	0.225583
C	-0.656088	1.862528	0.029183	C	-0.636172	1.723747	0.060527
N	0.554559	1.310434	-0.158331	N	0.586374	1.219581	-0.176205
C	0.674718	-0.018887	-0.340591	C	0.74109	-0.092784	-0.442026
C	-0.405822	-0.908183	-0.339957	C	-0.313771	-1.010853	-0.482295
C	2.038966	-0.601259	-0.604542	C	2.116322	-0.62049	-0.763259
N	3.155112	0.0303	-0.13738	N	3.225477	0.015391	-0.285176
O	2.142419	-1.652245	-1.25217	O	2.233338	-1.634528	-1.464706
C	-0.806239	3.309864	0.254924	C	-0.823909	3.150455	0.373242
O	-7.151559	-1.354022	0.240113	O	-6.997075	-1.893547	0.115142
C	-7.766786	-2.632863	0.1466	C	-7.955393	-0.883129	0.397467
C	4.451025	-0.574305	-0.450078	C	4.534158	-0.519696	-0.669282
C	5.464199	0.429048	0.148547	C	5.525704	0.501498	-0.064914
C	4.70773	1.020987	1.348488	C	4.790537	1.016591	1.182399
C	3.263345	1.135496	0.838444	C	3.324257	1.086765	0.728575
C	-0.060336	4.333948	-0.213178	C	-0.113265	4.221242	-0.042395
C	-0.389119	5.752756	0.185728	C	-0.476394	5.604495	0.44228
C	1.112184	4.196463	-1.149168	C	1.050513	4.172357	-0.998041
C	4.598991	-1.954117	0.198887	C	4.816013	-1.939108	-0.16616
O	5.368522	-2.757719	-0.562611	O	4.156966	-2.241429	0.970491
C	5.603076	-4.079938	-0.044637	C	4.36716	-3.572129	1.477969
O	4.155268	-2.270015	1.283398	O	5.611105	-2.681897	-0.703572

H	-3.505085	2.279907	0.254432	H	-3.493094	2.040524	0.35028
H	-2.922791	-3.060118	-0.32979	H	-2.76965	-3.236377	-0.576519
H	-5.356588	-3.372861	-0.154079	H	-5.226186	-3.607155	-0.380635
H	-5.948555	0.86781	0.405971	H	-5.864394	0.582805	0.447538
H	-0.228536	-1.964452	-0.508727	H	-0.110189	-2.048233	-0.723196
H	-1.651997	3.574768	0.892779	H	-1.668772	3.35319	1.034768
H	-7.578581	-3.096777	-0.830235	H	-7.773895	-0.424904	1.378795
H	-7.421429	-3.302129	0.945115	H	-7.954468	-0.10577	-0.378073
H	-8.836995	-2.45291	0.262142	H	-8.923479	-1.386637	0.40558
H	4.566882	-0.697098	-1.52946	H	4.617222	-0.582676	-1.757154
H	5.678478	1.204537	-0.596924	H	5.678513	1.315074	-0.784283
H	6.412441	-0.045504	0.419187	H	6.501894	0.055918	0.147069
H	4.754265	0.332477	2.198719	H	4.899374	0.304342	2.007829
H	5.108514	1.98882	1.667867	H	5.160704	1.989096	1.523544
H	3.081568	2.094496	0.343389	H	3.085785	2.055284	0.278892
H	2.5216	1.025033	1.632116	H	2.616094	0.9171	1.542647
H	0.47002	6.225108	0.68348	H	-0.730821	6.261692	-0.401323
H	-0.612122	6.367384	-0.697979	H	-1.322807	5.59295	1.137196
H	-1.245285	5.805361	0.866611	H	0.376593	6.072949	0.954009
H	1.219158	3.185026	-1.541301	H	0.924135	4.93087	-1.783189
H	1.012332	4.905772	-1.982654	H	1.985676	4.419208	-0.474178
H	2.047414	4.454471	-0.630835	H	1.176642	3.191165	-1.455902
H	6.22906	-4.574689	-0.78791	H	3.7544	-3.639001	2.37758
H	6.114663	-4.028174	0.920568	H	4.04757	-4.30921	0.736788
H	4.653948	-4.608702	0.075186	H	5.423053	-3.72956	1.714859

**Conf. 1-3**	**X axis(Å)**	**Y axis(Å)**	**Z axis(Å)**	**Conf. 1-4**	**X axis(Å)**	**Y axis(Å)**	**Z axis(Å)**
C	-1.765376	-0.670228	-0.144093	C	-1.637463	-0.337534	-0.185098
C	-1.78088	0.746893	-0.13895	C	-1.693322	1.052297	0.085953
C	-3.145234	-1.103783	-0.079186	C	-2.999011	-0.830435	-0.125792
C	-3.937998	0.068469	-0.030147	C	-3.82448	0.287992	0.172562
N	-3.099202	1.173667	-0.082725	N	-3.014872	1.40796	0.310899
C	-3.78336	-2.354841	-0.047606	C	-3.596982	-2.081337	-0.302824
C	-5.165373	-2.412871	0.03326	C	-4.980496	-2.210677	-0.187769
C	-5.938069	-1.227338	0.084639	C	-5.77908	-1.08283	0.106737
C	-5.336111	0.033478	0.052406	C	-5.20762	0.18156	0.291735
C	-0.586387	1.491288	-0.179707	C	-0.527477	1.843828	0.091841
N	0.586218	0.838318	-0.235539	N	0.656799	1.259883	-0.157597
C	0.617406	-0.509013	-0.233314	C	0.721801	-0.059062	-0.425375
C	-0.528139	-1.312622	-0.183874	C	-0.393173	-0.904723	-0.455877
C	1.943475	-1.232995	-0.250586	C	2.054826	-0.678547	-0.759582
N	3.061458	-0.638729	-0.764574	N	3.211043	-0.110969	-0.307785

O	2.000445	-2.389835	0.189198	O	2.094383	-1.70727	-1.448567
C	-0.638664	2.963758	-0.201149	C	-0.618131	3.27939	0.408118
O	-7.286829	-1.42516	0.164944	O	-7.139369	-1.128889	0.234068
C	-8.142172	-0.292205	0.226296	C	-7.807459	-2.372868	0.063071
C	4.286689	-1.43934	-0.826605	C	4.474482	-0.737578	-0.704717
C	5.140681	-0.713602	-1.889589	C	5.542	0.224802	-0.134385
C	4.71169	0.753807	-1.745973	C	4.864868	0.80849	1.115655
C	3.208369	0.671433	-1.436993	C	3.397975	0.967019	0.686576
C	0.22707	3.857094	0.324075	C	0.147664	4.303844	-0.02571
C	-0.01413	5.337844	0.152754	C	-0.118211	5.707111	0.464514
C	1.459292	3.504943	1.116536	C	1.282679	4.182424	-1.008958
C	5.033725	-1.57328	0.504156	C	4.670624	-2.164722	-0.183361
O	4.641321	-0.686711	1.43733	O	4.012428	-2.405948	0.968355
C	5.292682	-0.79697	2.716841	C	4.144043	-3.739635	1.493896
O	5.922649	-2.383617	0.673477	O	5.405775	-2.966527	-0.721349
H	-3.39225	2.12872	0.057058	H	-3.352435	2.353829	0.404852
H	-3.202255	-3.272473	-0.083078	H	-2.993026	-2.955112	-0.533076
H	-5.688737	-3.3632	0.061179	H	-5.433968	-3.1845	-0.328976
H	-5.915102	0.949433	0.086898	H	-5.846445	1.029364	0.51968
H	-0.421965	-2.391335	-0.165194	H	-0.261363	-1.952961	-0.69964
H	-1.514104	3.371269	-0.711512	H	-1.433324	3.534037	1.088576
H	-7.933325	0.316695	1.115963	H	-7.654208	-2.773262	-0.947336
H	-8.047321	0.327901	-0.675029	H	-7.476364	-3.109901	0.805986
H	-9.1566	-0.689538	0.288311	H	-8.867388	-2.159564	0.211575
H	4.050289	-2.464773	-1.121302	H	4.533589	-0.822696	-1.792649
H	4.870563	-1.097233	-2.880692	H	5.736171	1.01417	-0.87056
H	6.2114	-0.881497	-1.745445	H	6.489867	-0.281438	0.069718
H	5.240718	1.222391	-0.907426	H	4.942328	0.105275	1.952288
H	4.914302	1.348525	-2.642716	H	5.304285	1.760302	1.431963
H	2.605345	0.692052	-2.354455	H	3.213994	1.942112	0.225664
H	2.858968	1.476471	-0.79404	H	2.695603	0.856092	1.515538
H	-0.915806	5.547156	-0.432461	H	-0.947539	5.747769	1.178594
H	-0.112046	5.834915	1.128336	H	0.773581	6.122555	0.955427
H	0.837469	5.814266	-0.353879	H	-0.35132	6.378104	-0.374385
H	1.486977	4.089986	2.046525	H	2.242683	4.385216	-0.511772
H	1.516365	2.442528	1.354624	H	1.344277	3.191053	-1.458072
H	2.366238	3.77188	0.554458	H	1.176508	4.938039	-1.799897
H	4.836163	-0.027416	3.339995	H	5.191332	-3.962354	1.716174
H	5.121579	-1.789719	3.140827	H	3.543875	-3.752611	2.404334
H	6.368008	-0.626714	2.614159	H	3.764435	-4.465437	0.770062

**Conf. 1-5**	**X axis(Å)**	**Y axis(Å)**	**Z axis(Å)**	**Conf. 1-6**	**X axis(Å)**	**Y axis(Å)**	**Z axis(Å)**

H	-3.264815	2.414911	0.07079	C	-1.470631	-0.508853	-0.215524
H	-3.383796	-2.98483	-0.090658	C	-1.675403	0.8941	-0.296546
H	-5.840693	-2.962732	0.047892	C	-2.782021	-1.114708	-0.097329
H	-5.885638	1.354783	0.097479	C	-3.726521	-0.051673	-0.127439
H	-0.561509	-2.270405	-0.168119	N	-3.036029	1.146884	-0.235612
H	-1.318775	3.546486	-0.692321	C	-3.24785	-2.427486	0.016325
H	-7.893068	-2.505228	1.07309	C	-4.618221	-2.672317	0.096859
H	-9.176965	-1.567098	0.258599	C	-5.536314	-1.598815	0.063128
H	-7.996095	-2.485762	-0.717983	C	-5.099069	-0.273907	-0.049527
H	3.89416	-2.600589	-1.136729	C	-0.590995	1.774686	-0.445254
H	4.785162	-1.273767	-2.893124	N	0.65098	1.274107	-0.549808
H	6.142349	-1.145456	-1.764321	C	0.860056	-0.053483	-0.471126
H	5.307249	1.007708	-0.909992	C	-0.166756	-0.991116	-0.301049
H	4.975142	1.163404	-2.641646	C	2.257527	-0.593772	-0.63187
H	2.631912	0.653957	-2.335962	N	3.339444	0.212236	-0.425691
H	2.948474	1.402271	-0.769532	O	2.420159	-1.776961	-0.963369
H	-0.598009	5.682987	-0.406735	C	-0.733567	3.243284	-0.581956
H	0.218938	5.919525	1.155623	O	-6.892261	-1.757797	0.136599
H	1.16755	5.850518	-0.325876	C	-7.427863	-3.069339	0.26316
H	2.577878	3.725434	0.579273	C	4.661634	-0.37787	-0.642027
H	1.713251	4.081687	2.071143	C	5.62233	0.817457	-0.445477
H	1.654781	2.439432	1.369464	C	4.885656	1.698337	0.575572
H	4.865355	-0.223676	3.325071	C	3.411452	1.566266	0.164624
H	5.026739	-2.00155	3.125238	C	-1.377175	4.096972	0.239602
H	6.346468	-0.927812	2.587892	C	-1.413106	5.575847	-0.06658
C	-1.801548	-0.474365	-0.139635	C	-2.04099	3.702211	1.537535
C	-1.736768	0.940929	-0.12864	C	4.952741	-1.489393	0.371128
C	-3.205463	-0.829125	-0.077098	O	5.752864	-2.429649	-0.171418
C	-3.932192	0.391744	-0.023469	C	6.120231	-3.518792	0.694657
C	-3.911113	-2.035279	-0.05169	O	4.584122	-1.499486	1.527313
C	-5.303151	-2.022225	0.027824	H	-3.454688	2.057379	-0.352503
C	-6.001798	-0.795388	0.083133	H	-2.550565	-3.260758	0.04135
C	-5.321731	0.427873	0.056675	H	-4.968832	-3.693751	0.184036
C	-0.500773	1.614892	-0.167465	H	-5.828051	0.530364	-0.071935
C	0.586205	-0.451232	-0.229584	H	0.076739	-2.047239	-0.272269
C	-0.604656	-1.187262	-0.18216	H	-0.198502	3.658941	-1.436027
C	1.867519	-1.251463	-0.252218	H	-7.170306	-3.691813	-0.603418
C	-0.467846	3.087771	-0.184079	H	-7.079373	-3.555917	1.183229
C	-8.137517	-1.893479	0.195173	H	-8.510719	-2.941119	0.308314
C	4.192665	-1.592761	-0.837856	H	4.728545	-0.81147	-1.642499
C	5.082846	-0.912769	-1.901367	H	5.734735	1.342971	-1.401585
C	4.743958	0.576921	-1.746404	H	6.618136	0.504192	-0.11708
C	3.240801	0.584555	-1.42498	H	5.0323	1.304423	1.586876

C	0.446941	3.928573	0.34467	H	5.223942	2.739793	0.560458
C	0.289893	5.421155	0.178364	H	3.125868	2.317239	-0.578884
C	1.656199	3.504742	1.137451	H	2.724931	1.657909	1.008198
C	4.937444	-1.778243	0.487899	H	-0.913123	5.810885	-1.011553
C	5.262784	-1.023138	2.699105	H	-2.448196	5.941412	-0.128838
N	-3.027579	1.444759	-0.070826	H	-0.926899	6.152885	0.732714
N	0.632197	0.894992	-0.226918	H	-1.89603	2.649846	1.790983
N	3.017512	-0.721323	-0.765492	H	-1.633837	4.306816	2.359803
O	1.856786	-2.412398	0.180629	H	-3.121874	3.907259	1.517265
O	-7.363246	-0.700667	0.163414	H	5.225199	-4.050285	1.028252
O	4.610676	-0.868781	1.424524	H	6.751929	-4.169413	0.089053
O	5.77239	-2.645372	0.650459	H	6.667964	-3.147816	1.565528

Conf. 1-7	X axis(Å)	Y axis(Å)	Z axis(Å)	Conf. 1-8	X axis(Å)	Y axis(Å)	Z axis(Å)
C	-1.395718	-0.66513	-0.265514	C	-1.360962	-0.611554	-0.295913
C	-1.619821	0.737297	-0.292669	C	-1.605685	0.741723	0.059711
C	-2.694959	-1.291982	-0.144581	C	-2.636428	-1.293889	-0.240552
C	-3.650664	-0.246646	-0.118523	C	-3.595735	-0.331999	0.161119
N	-2.982398	0.966466	-0.195647	N	-2.957022	0.887987	0.326103
C	-3.142405	-2.6215	-0.071493	C	-3.057185	-2.612921	-0.477706
C	-4.500451	-2.879225	0.023038	C	-4.392661	-2.942448	-0.31154
C	-5.438535	-1.818557	0.045197	C	-5.334069	-1.965474	0.0945
C	-5.028445	-0.484465	-0.024785	C	-4.950642	-0.643666	0.336931
C	-0.551122	1.63767	-0.433188	C	-0.556649	1.673602	0.124019
N	0.694809	1.159307	-0.579944	N	0.701627	1.260611	-0.098814
C	0.923943	-0.167857	-0.551215	C	0.94821	-0.017348	-0.446191
C	-0.086357	-1.124961	-0.393625	C	-0.049534	-0.991575	-0.575341
C	2.325231	-0.685678	-0.756167	C	2.359096	-0.4437	-0.765108
N	3.402936	0.133181	-0.578776	N	3.423963	0.294137	-0.33502
O	2.493936	-1.866172	-1.093376	O	2.546884	-1.472268	-1.430194
C	-0.71823	3.107985	-0.516797	C	-0.736577	3.093364	0.507484
O	-6.743256	-2.209851	0.13954	O	-6.614445	-2.422323	0.221914
C	-7.756656	-1.214228	0.167817	C	-7.629659	-1.512747	0.623063
C	4.727762	-0.430147	-0.853514	C	4.760038	-0.154909	-0.736305
C	5.669786	0.787843	-0.711782	C	5.683161	0.977521	-0.230104
C	4.961044	1.665923	0.330503	C	4.948111	1.507669	1.010248
C	3.473177	1.497804	-0.012781	C	3.46723	1.432631	0.607598
C	-1.350048	3.923387	0.351378	C	-1.581197	3.999051	-0.025806
C	-1.416618	5.41111	0.097527	C	-1.617713	5.415381	0.498198
C	-1.970304	3.473884	1.653183	C	-2.488923	3.736597	-1.204054
C	5.142291	-1.57086	0.081162	C	5.174115	-1.513907	-0.163104
O	4.56014	-1.503693	1.295342	O	4.572163	-1.803352	1.008167

C	4.898186	-2.564034	2.208335	C	4.908657	-3.079098	1.583835
O	5.968626	-2.405144	-0.225646	O	6.014786	-2.218831	-0.681962
H	-3.415855	1.874133	-0.275176	H	-3.384397	1.736606	0.665359
H	-2.433325	-3.444762	-0.090955	H	-2.345008	-3.372295	-0.789351
H	-4.87875	-3.89481	0.080059	H	-4.750217	-3.951994	-0.486897
H	-5.735637	0.337093	-0.008043	H	-5.660741	0.114773	0.646591
H	0.172089	-2.177795	-0.408723	H	0.224415	-1.996425	-0.87648
H	-0.214879	3.560258	-1.371549	H	-0.049538	3.43068	1.283815
H	-7.64231	-0.554149	1.037965	H	-8.552718	-2.093898	0.654719
H	-7.752311	-0.614916	-0.752313	H	-7.423774	-1.099961	1.619617
H	-8.701389	-1.754972	0.244265	H	-7.738723	-0.693339	-0.099843
H	4.758408	-0.861542	-1.857069	H	4.81217	-0.276885	-1.820969
H	5.725717	1.306337	-1.676574	H	5.744923	1.754972	-1.001067
H	6.685737	0.495842	-0.430692	H	6.698979	0.625552	-0.02852
H	5.161338	1.291302	1.340568	H	5.139952	0.857686	1.871305
H	5.275082	2.714146	0.289075	H	5.24778	2.525659	1.280464
H	3.14164	2.231794	-0.754138	H	3.133503	2.348141	0.108951
H	2.820459	1.59186	0.856977	H	2.803331	1.25825	1.4566
H	-0.91271	5.968143	0.900037	H	-0.940979	5.556059	1.347019
H	-0.950915	5.68542	-0.854322	H	-1.336392	6.130158	-0.288002
H	-2.458168	5.7629	0.081214	H	-2.631879	5.69139	0.820946
H	-1.548489	4.055932	2.484305	H	-2.35316	2.743813	-1.638348
H	-3.054195	3.662825	1.67077	H	-3.548547	3.853747	-0.931208
H	-1.802961	2.415587	1.865142	H	-2.295801	4.478537	-1.991165
H	4.606444	-3.52876	1.785258	H	5.979974	-3.132955	1.796506
H	5.972688	-2.566707	2.411493	H	4.32744	-3.143569	2.50433
H	4.332767	-2.354742	3.11707	H	4.634319	-3.883483	0.896452

Table S3. The Cartesian coordinates of the lowest energy conformers (Conf. 1-9) for **isomer 2** of compounds **1a/1b**.

Conf. 2-1	X axis(Å)	Y axis(Å)	Z axis(Å)	Conf. 2-2	X axis(Å)	Y axis(Å)	Z axis(Å)
C	1.586476	-0.563064	-0.35575	C	-1.455546	-0.981949	-0.206204
C	1.3086	0.821657	-0.241286	C	-1.254865	0.395663	0.078442
C	3.013634	-0.720445	-0.15817	C	-2.883587	-1.213095	-0.158752
C	3.541274	0.581565	0.060333	C	-3.485914	0.02627	0.17041
N	2.493943	1.493162	0.021673	N	-2.49019	0.982435	0.297911
C	3.886692	-1.811686	-0.152391	C	-3.705114	-2.336917	-0.347746
C	5.249317	-1.607998	0.062455	C	-5.077537	-2.209262	-0.207185
C	5.749383	-0.303636	0.273632	C	-5.657942	-0.961085	0.125953
C	4.898135	0.807723	0.276051	C	-4.871162	0.177351	0.319714
C	0.000145	1.317793	-0.392271	C	0.037429	0.94494	0.132513
N	-0.999176	0.452836	-0.639824	N	1.095545	0.130833	-0.021729
C	-0.747599	-0.864881	-0.761176	C	0.921796	-1.175634	-0.297668

C	0.528208	-1.42637	-0.632986	C	-0.333616	-1.781017	-0.423265
C	-1.872518	-1.819483	-1.090396	C	2.134715	-2.040743	-0.53176
N	-3.168314	-1.521776	-0.768941	N	3.340897	-1.687335	0.004447
O	-1.604298	-2.884349	-1.659536	O	2.028527	-3.070969	-1.20864
C	-0.26421	2.758768	-0.237475	C	0.330799	2.363913	0.43493
O	7.070085	-0.0228	0.489354	O	-7.01897	-0.973634	0.235675
C	8.008564	-1.091226	0.512084	C	-7.689874	0.233423	0.570415
C	-3.691339	-0.403114	0.016293	C	3.632363	-0.659219	1.006626
C	-5.223313	-0.544625	-0.15831	C	4.922215	-1.193673	1.679173
C	-5.429237	-2.062568	-0.27253	C	5.644535	-1.923522	0.535979
C	-4.201952	-2.535973	-1.062791	C	4.499145	-2.563032	-0.26117
C	-1.179149	3.530013	-0.862952	C	-0.190506	3.464443	-0.145856
C	-1.29874	4.99521	-0.518627	C	0.239721	4.840389	0.306117
C	-2.127602	3.052079	-1.93187	C	-1.15464	3.453251	-1.308688
C	-3.289185	-0.490799	1.492083	C	3.856509	0.750678	0.455332
O	-3.566287	0.674537	2.119098	O	4.212193	0.766444	-0.841021
C	-3.246503	0.728121	3.521896	C	4.429904	2.066654	-1.416827
O	-2.810977	-1.456177	2.047843	O	3.798006	1.743942	1.154649
H	2.603301	2.495953	0.022154	H	-2.624541	1.938191	0.591664
H	3.51283	-2.81893	-0.316244	H	-3.272648	-3.300837	-0.602419
H	5.917366	-2.460909	0.063836	H	-5.739757	-3.057648	-0.34682
H	5.311031	1.797583	0.445043	H	-5.302403	1.139307	0.572784
H	0.655073	-2.495488	-0.759014	H	-0.395852	-2.835078	-0.67007
H	0.386833	3.256747	0.484361	H	1.115493	2.499177	1.178414
H	7.783152	-1.803996	1.315782	H	-7.372059	0.606327	1.553125
H	8.038262	-1.61826	-0.450309	H	-7.520228	1.007396	-0.189884
H	8.97788	-0.626565	0.700775	H	-8.751544	-0.016733	0.603079
H	-3.323012	0.548456	-0.362955	H	2.819222	-0.565204	1.727172
H	-5.515375	-0.045625	-1.090177	H	4.637528	-1.902126	2.466429
H	-5.779911	-0.079885	0.660455	H	5.509285	-0.395007	2.141692
H	-5.435583	-2.520162	0.723591	H	6.190853	-1.207161	-0.087775
H	-6.368747	-2.326138	-0.768575	H	6.360743	-2.668866	0.896055
H	-4.38827	-2.565894	-2.144278	H	4.270364	-3.580627	0.079402
H	-3.846857	-3.526001	-0.765456	H	4.691825	-2.616438	-1.336826
H	-0.593987	5.296961	0.263397	H	0.955662	4.793594	1.132283
H	-1.121857	5.622782	-1.403641	H	0.703614	5.397472	-0.52028
H	-2.314541	5.230164	-0.169474	H	-0.624711	5.435455	0.634903
H	-2.114238	3.745753	-2.784093	H	-0.751636	4.065449	-2.127398
H	-1.893371	2.046136	-2.28161	H	-1.343807	2.451812	-1.701238
H	-3.160163	3.043877	-1.552966	H	-2.120121	3.908239	-1.039842
H	-3.814917	-0.026285	4.072944	H	3.511498	2.657047	-1.363015
H	-2.177601	0.556133	3.672976	H	5.231371	2.591242	-0.889115
H	-3.525157	1.731993	3.843986	H	4.707068	1.880262	-2.454918

Conf. 2-3	X axis(Å)	Y axis(Å)	Z axis(Å)	Conf. 2-4	X axis(Å)	Y axis(Å)	Z axis(Å)
C	1.504331	-0.796771	0.163582	C	1.446663	-0.769249	-0.234139
C	1.213248	0.562764	-0.128208	C	1.196129	0.620585	-0.079914
C	2.94589	-0.934524	0.111049	C	2.879387	-0.949734	-0.109713
C	3.465536	0.344766	-0.230177	C	3.433185	0.337385	0.133896
N	2.405294	1.229777	-0.35812	N	2.402387	1.265738	0.136465
C	3.836069	-1.994938	0.302939	C	3.735669	-2.053024	-0.164473
C	5.206587	-1.784721	0.156209	C	5.10642	-1.876553	0.020431
C	5.697581	-0.504901	-0.187408	C	5.631578	-0.587606	0.264526
C	4.830296	0.575891	-0.384315	C	4.79859	0.535793	0.323205
C	-0.113341	1.024642	-0.177851	C	-0.114091	1.126301	-0.12323
N	-1.114475	0.14243	-0.01326	N	-1.137546	0.26487	-0.252192
C	-0.85422	-1.147714	0.267887	C	-0.918662	-1.053502	-0.407532
C	0.440191	-1.667462	0.389253	C	0.361578	-1.623076	-0.41844
C	-2.003554	-2.093402	0.511529	C	-2.086252	-2.000089	-0.557367
N	-3.240607	-1.816436	0.001691	N	-3.37334	-1.539752	-0.605954
O	-1.816665	-3.122061	1.173641	O	-1.867511	-3.216595	-0.637179
C	-0.502467	2.418517	-0.487695	C	-0.470022	2.552872	0.045479
O	7.024716	-0.220593	-0.350695	O	6.960942	-0.334095	0.457934
C	7.980744	-1.257674	-0.167653	C	7.883371	-1.416109	0.421714
C	-3.624009	-0.795358	-0.976161	C	-3.892622	-0.16853	-0.582337
C	-4.89205	-1.404183	-1.62664	C	-5.353138	-0.34319	-1.066978
C	-5.536224	-2.195003	-0.477296	C	-5.742389	-1.732819	-0.54275
C	-4.331576	-2.77127	0.279455	C	-4.450846	-2.546872	-0.693899
C	-0.048877	3.556999	0.076605	C	0.039621	3.623759	-0.597439
C	-0.574472	4.896366	-0.384215	C	-0.471333	5.011263	-0.287941
C	0.926471	3.622191	1.22807	C	1.065818	3.560716	-1.703986
C	-3.928143	0.588333	-0.397301	C	-3.851899	0.517146	0.786283
O	-4.232765	0.565483	0.91181	O	-3.650179	-0.335481	1.808442
C	-4.522044	1.840568	1.511839	C	-3.605724	0.253578	3.120419
O	-3.967963	1.590046	-1.085568	O	-4.042237	1.709054	0.930301
H	2.475233	2.190136	-0.65908	H	2.496711	2.247289	0.349549
H	3.469211	-2.983555	0.566228	H	3.341969	-3.049061	-0.349348
H	5.888139	-2.613209	0.308076	H	5.761145	-2.738568	-0.024744
H	5.237099	1.547926	-0.645826	H	5.231199	1.513591	0.511682
H	0.573674	-2.714057	0.639424	H	0.463732	-2.695239	-0.540695
H	-1.302608	2.495374	-1.222951	H	-1.300755	2.718083	0.730621
H	7.949423	-1.653971	0.855438	H	8.863562	-0.972349	0.604332
H	7.826398	-2.074559	-0.884336	H	7.66533	-2.153536	1.2049
H	8.952974	-0.795911	-0.348121	H	7.883532	-1.908975	-0.559046
H	-2.837123	-0.636113	-1.714173	H	-3.32751	0.483006	-1.246892
H	-4.581117	-2.082073	-2.430774	H	-5.359871	-0.332696	-2.163605

H	-5.540695	-0.639254	-2.063031	H	-6.000754	0.466467	-0.719146
H	-6.109046	-1.523465	0.172069	H	-6.034824	-1.674312	0.512276
H	-6.213683	-2.978969	-0.830253	H	-6.576314	-2.176547	-1.095831
H	-4.045784	-3.765709	-0.085787	H	-4.393086	-3.060081	-1.662448
H	-4.492306	-2.855701	1.35834	H	-4.316988	-3.305558	0.082126
H	-1.293177	4.793429	-1.202855	H	-1.22618	5.001581	0.504173
H	-1.06728	5.429468	0.441269	H	0.348585	5.672861	0.02727
H	0.245093	5.544566	-0.72712	H	-0.917301	5.471332	-1.181022
H	1.183746	2.64006	1.630544	H	0.686134	4.091985	-2.58769
H	1.857928	4.134272	0.942706	H	1.998601	4.072569	-1.422373
H	0.494028	4.217808	2.043945	H	1.311426	2.540511	-2.006556
H	-5.380455	2.309367	1.022584	H	-3.450741	-0.579951	3.806103
H	-4.743076	1.624246	2.557564	H	-2.778046	0.964949	3.186265
H	-3.652461	2.497803	1.429488	H	-4.544364	0.769312	3.341002

**Conf. 2-5**	**X axis(Å)**	**Y axis(Å)**	**Z axis(Å)**	**Conf. 2-6**	**X axis(Å)**	**Y axis(Å)**	**Z axis(Å)**
C	1.4918	-1.015334	0.089939	C	1.395828	-0.955014	-0.244773
C	1.26692	0.364537	-0.160026	C	1.237812	0.450392	-0.107289
C	2.926328	-1.209827	0.091866	C	2.812316	-1.228105	-0.123885
C	3.508758	0.054224	-0.171722	C	3.44962	0.017083	0.099514
N	2.49385	0.9896	-0.309169	N	2.486294	1.014572	0.09494
C	3.768246	-2.318648	0.278598	C	3.596538	-2.392729	-0.167788
C	5.141516	-2.152553	0.199123	C	4.967491	-2.298174	0.009188
C	5.702125	-0.880026	-0.068772	C	5.583255	-1.043007	0.234821
C	4.894346	0.244545	-0.258262	C	4.83393	0.13555	0.282873
C	-0.036139	0.880044	-0.249146	C	-0.035698	1.041985	-0.151181
N	-1.080744	0.041839	-0.144649	N	-1.11487	0.250067	-0.265825
C	-0.884897	-1.268017	0.102764	C	-0.984264	-1.082366	-0.406259
C	0.383547	-1.845841	0.240998	C	0.254245	-1.736424	-0.414821
C	-2.066957	-2.19307	0.275617	C	-2.213091	-1.950963	-0.540919
N	-3.328479	-1.80506	-0.077255	N	-3.467922	-1.407558	-0.580849
O	-1.879732	-3.325328	0.7396	O	-2.075702	-3.179532	-0.614478
C	-0.336816	2.30131	-0.541154	C	-0.293942	2.491466	0.001343
O	7.066438	-0.85474	-0.121762	O	6.938439	-1.091003	0.395605
C	7.71828	0.3796	-0.387013	C	7.642818	0.11928	0.636484
C	-3.782578	-0.590383	-0.753388	C	-3.897195	-0.005248	-0.563111
C	-5.153701	-1.016366	-1.337262	C	-5.373586	-0.089165	-1.02355
C	-5.686365	-2.011426	-0.29502	C	-5.842543	-1.44713	-0.481826
C	-4.427919	-2.763071	0.158678	C	-4.609298	-2.343508	-0.649829
C	0.115182	3.394263	0.106687	C	0.279711	3.516632	-0.661659
C	-0.291792	4.777147	-0.346111	C	-0.132707	4.939949	-0.368038
C	0.9788	3.36212	1.345192	C	1.287249	3.368549	-1.777324

C	-3.9568	0.581675	0.215153	C	-3.791563	0.689634	0.797146
O	-4.014277	1.748465	-0.467759	O	-3.631488	-0.164089	1.825606
C	-4.23293	2.926639	0.327737	C	-3.530898	0.433529	3.13054
O	-4.105435	0.497308	1.414704	O	-3.902066	1.892818	0.930941
H	2.615012	1.957767	-0.565683	H	2.647066	1.990298	0.294718
H	3.350587	-3.300613	0.484134	H	3.136208	-3.362388	-0.3377
H	5.819395	-2.98845	0.338901	H	5.601462	-3.178574	-0.018716
H	5.310326	1.224808	-0.461799	H	5.292276	1.103136	0.45321
H	0.461187	-2.90676	0.449173	H	0.283755	-2.814351	-0.524727
H	-1.046296	2.445264	-1.35597	H	-1.104398	2.720843	0.692406
H	7.432647	0.777571	-1.369792	H	7.531272	0.819187	-0.202317
H	7.495504	1.122627	0.390249	H	7.304018	0.599108	1.564267
H	8.786736	0.157862	-0.381707	H	8.692151	-0.163205	0.736031
H	-3.088419	-0.281205	-1.533728	H	-3.301759	0.602011	-1.243017
H	-4.983749	-1.517128	-2.298187	H	-5.397699	-0.086136	-2.120004
H	-5.81432	-0.162589	-1.514988	H	-5.962395	0.762799	-0.672221
H	-6.131694	-1.473599	0.549186	H	-6.110335	-1.362893	0.578004
H	-6.445036	-2.686424	-0.703638	H	-6.713566	-1.839271	-1.016361
H	-4.248922	-3.672891	-0.428171	H	-4.599778	-2.861239	-1.6177
H	-4.450091	-3.054377	1.212438	H	-4.512568	-3.107637	0.126257
H	-0.922613	4.746268	-1.240193	H	-0.876477	4.993215	0.432727
H	-0.841856	5.303882	0.44667	H	0.734049	5.549134	-0.073008
H	0.590146	5.393318	-0.572839	H	-0.557769	5.41591	-1.263
H	0.492811	3.931561	2.149516	H	2.254576	3.822803	-1.513668
H	1.161227	2.350767	1.714537	H	1.461994	2.329671	-2.065303
H	1.950271	3.849529	1.173228	H	0.933487	3.909247	-2.666056
H	-5.170383	2.84518	0.884905	H	-4.42885	1.015337	3.356021
H	-3.405831	3.065092	1.028999	H	-3.425687	-0.401817	3.823413
H	-4.278688	3.752981	-0.382844	H	-2.655169	1.086395	3.179557

**Conf. 2-7**	**X axis(Å)**	**Y axis(Å)**	**Z axis(Å)**	**Conf. 2-8**	**X axis(Å)**	**Y axis(Å)**	**Z axis(Å)**
H	2.457323	2.216578	-0.605365	C	1.694868	-0.65506	-0.101643
H	3.547625	-2.976239	0.446472	C	1.364621	0.720132	-0.040719
H	5.962916	-2.538369	0.270893	C	3.140559	-0.739999	-0.037432
H	5.234416	1.640256	-0.539357	C	3.623018	0.59492	0.054056
H	0.643268	-2.776872	0.451692	N	2.533694	1.456571	0.065885
H	-1.231589	2.460769	-1.348416	C	4.062574	-1.79019	-0.05438
H	7.986074	-1.553714	0.9087	C	5.427941	-1.514979	0.013225
H	7.916357	-1.907949	-0.848636	C	5.881855	-0.17978	0.099008
H	8.997919	-0.625435	-0.234283	C	4.981668	0.891998	0.121247
H	-3.072452	-0.42115	-1.522441	C	0.027766	1.155166	-0.090295
H	-4.869546	-1.803613	-2.275378	N	-0.952616	0.242156	-0.202393

H	-5.799689	-0.496789	-1.524185	C	-0.654751	-1.07377	-0.258158
H	-6.042172	-1.789107	0.562241	C	0.652994	-1.573841	-0.208613
H	-6.255844	-3.044322	-0.669185	C	-1.753089	-2.111779	-0.326268
H	-4.000252	-3.868466	-0.354878	N	-3.070871	-1.763647	-0.431967
H	-4.256071	-3.228882	1.269973	O	-1.445831	-3.310925	-0.27647
H	-1.257515	4.766272	-1.232634	C	-0.276954	2.593291	0.04695
H	-1.191573	5.331208	0.452583	O	7.20178	0.170073	0.167366
H	0.219419	5.508063	-0.584679	C	8.189242	-0.853391	0.155332
H	0.242246	4.046197	2.142488	C	-3.69491	-0.449693	-0.589959
H	1.006136	2.511298	1.699868	C	-5.101257	-0.797153	-1.135027
H	1.692064	4.057065	1.151371	C	-5.418685	-2.141449	-0.463183
H	-5.392772	2.594657	0.820842	C	-4.062563	-2.859348	-0.440428
H	-3.648442	2.929524	0.988153	C	-1.1682	3.335014	-0.641491
H	-4.54083	3.539276	-0.447286	C	-1.375729	4.790426	-0.301821
C	1.540781	-0.823226	0.073698	C	-2.016228	2.819728	-1.77372
C	1.223767	0.537945	-0.176549	C	-3.802129	0.38658	0.688688
C	2.986364	-0.924319	0.055493	O	-3.488985	-0.287114	1.807678
C	3.482787	0.37953	-0.221036	C	-3.551404	0.466948	3.032351
C	3.896999	-1.969756	0.231699	O	-4.186171	1.540768	0.678654
C	5.265306	-1.721168	0.131664	H	2.588287	2.462523	0.007992
C	5.733285	-0.417646	-0.148446	H	3.724513	-2.820919	-0.121851
C	4.844887	0.649259	-0.327327	H	6.1342	-2.336502	-0.001814
C	-0.111706	0.966338	-0.248654	H	5.359819	1.907331	0.191247
C	-0.813383	-1.231419	0.119251	H	0.808225	-2.645723	-0.245424
C	0.492693	-1.724115	0.241819	H	0.312791	3.103216	0.81272
C	-1.929275	-2.231225	0.31158	H	9.148315	-0.33673	0.220885
C	-0.507461	2.364348	-0.539462	H	8.078118	-1.526359	1.015337
C	8.033077	-1.116608	-0.097009	H	8.151253	-1.43484	-0.774896
C	-3.74863	-0.765798	-0.741085	H	-3.13923	0.16814	-1.292794
C	-5.081827	-1.298132	-1.325372	H	-5.035003	-0.919439	-2.222908
C	-5.551312	-2.308954	-0.26784	H	-5.82807	-0.00737	-0.927094
C	-4.246574	-2.960464	0.209902	H	-5.783243	-1.979276	0.558321
C	-0.120863	3.48485	0.103547	H	-6.179963	-2.715739	-1.000475
C	-0.619282	4.838347	-0.346179	H	-3.906568	-3.484424	-1.328629
C	0.755595	3.508889	1.333101	H	-3.920314	-3.500172	0.43433
C	-4.012173	0.406928	0.206306	H	-0.73783	5.118375	0.525796
C	-4.45356	2.728121	0.276726	H	-1.173122	5.431633	-1.171241
N	2.403934	1.243324	-0.345008	H	-2.422042	4.969466	-0.017129
N	-1.097846	0.061324	-0.128493	H	-3.060058	2.721991	-1.446854
N	-3.214711	-1.93396	-0.042033	H	-2.002267	3.530877	-2.611004
O	-1.664524	-3.341147	0.791775	H	-1.679423	1.845783	-2.13513
O	7.056851	-0.095644	-0.263998	H	-3.267855	-0.23447	3.817493
O	-4.147691	1.554813	-0.497149	H	-2.851814	1.305947	2.991402

O	-4.162588	0.332552	1.406273	H	-4.563764	0.846094	3.196891

Conf. 2-9	X axis(Å)	Y axis(Å)	Z axis(Å)
C	1.657843	-0.853364	-0.115309
C	1.415318	0.540647	-0.060629
C	3.093505	-1.029907	-0.049522
C	3.658792	0.266081	0.036871
N	2.630408	1.198747	0.043697
C	3.95015	-2.143102	-0.060288
C	5.320408	-1.949221	0.009626
C	5.86376	-0.644151	0.091859
C	5.040794	0.485231	0.107384
C	0.109898	1.061643	-0.113324
N	-0.927585	0.213755	-0.220806
C	-0.714392	-1.119445	-0.270521
C	0.557567	-1.703185	-0.21878
C	-1.877835	-2.084505	-0.333771
N	-3.17144	-1.651583	-0.425687
O	-1.648442	-3.301023	-0.289938
C	-0.099532	2.517469	0.015915
O	7.227116	-0.593634	0.154155
C	7.86152	0.674839	0.239379
C	-3.711144	-0.299435	-0.575466
C	-5.147653	-0.554939	-1.093087
C	-5.538127	-1.876521	-0.415217
C	-4.231845	-2.680924	-0.422336
C	-0.946857	3.311332	-0.670058
C	-1.056921	4.778836	-0.336648
C	-1.83843	2.844974	-1.789907
C	-3.738769	0.543705	0.702864
O	-3.465464	-0.151794	1.819023
C	-3.458655	0.604079	3.044071
O	-4.034094	1.723767	0.696173
H	2.750359	2.198769	-0.018107
H	3.546459	-3.150031	-0.124317
H	6.009199	-2.7879	0.002685
H	5.442232	1.490104	0.173711
H	0.643006	-2.783019	-0.251108
H	0.52758	2.993375	0.773827
H	7.639548	1.291321	-0.641872
H	7.559933	1.2109	1.149115
H	8.932454	0.468811	0.277784

H	-3.129582	0.279657	-1.290287
H	-5.110497	-0.680011	-2.182044
H	-5.81793	0.279915	-0.871418
H	-5.868465	-1.692685	0.614183
H	-6.346932	-2.398786	-0.935949
H	-4.133633	-3.30856	-1.317109
H	-4.115193	-3.335866	0.445657
H	-0.391269	5.068912	0.483238
H	-0.822062	5.401505	-1.211389
H	-2.086684	5.02617	-0.042867
H	-1.804046	3.558706	-2.624234
H	-1.556658	1.857186	-2.161011
H	-2.879582	2.796619	-1.444114
H	-3.219097	-0.116803	3.826246
H	-2.699629	1.389259	2.995431
H	-4.438492	1.05668	3.219591

Table S4. The Cartesian coordinates of the lowest energy conformers (conf. 1-10) for **isomers 7b** of compound **7**.

Conf. 7b-1	X axis(Å)	Y axis(Å)	Z axis(Å)	Conf. 7b-2	X axis(Å)	Y axis(Å)	Z axis(Å)
C	-0.550206	-1.367952	0.001552	C	-0.342031	-1.418887	-0.004794
C	0.685425	-0.461672	0.225255	C	0.80781	-0.408873	0.230532
C	1.923224	-1.406185	-0.066051	C	2.126255	-1.242413	-0.044972
N	1.334626	-2.505663	-0.792448	N	1.643227	-2.397374	-0.762084
C	-0.02657	-2.504494	-0.899498	C	0.288473	-2.511605	-0.890521
O	0.727014	0.161338	1.477744	O	0.781042	0.214328	1.483876
C	3.066183	-0.799305	-0.954762	C	3.224302	-0.553372	-0.931382
C	3.846605	0.393572	-0.525188	C	3.881193	0.719253	-0.524989
O	3.314576	-1.368507	-2.011859	O	3.54989	-1.129617	-1.963499
C	3.675494	1.037021	0.713607	C	3.613093	1.387721	0.682883
C	4.454387	2.150162	1.036199	C	4.278364	2.577559	0.985344
C	5.405754	2.63142	0.133934	C	5.212831	3.11008	0.094194
C	5.581112	1.997465	-1.102784	C	5.484545	2.451152	-1.111661
C	4.809538	0.887155	-1.429373	C	4.825312	1.265425	-1.418663
O	-0.708417	-3.230942	-1.595906	O	-0.318715	-3.297802	-1.590881
O	-1.607495	-0.69856	-0.697611	O	-1.44141	-0.842291	-0.720686
C	-2.780457	-0.945953	-0.032489	C	-2.599243	-1.183917	-0.070058
C	-2.642562	-1.626797	1.133179	C	-2.420986	-1.848194	1.099687
C	-1.220571	-1.8917	1.291147	C	-0.983943	-1.995311	1.276863
O	-0.620047	-2.404954	2.226115	O	-0.35572	-2.459229	2.219201
C	-3.673942	-2.042653	2.134402	C	-3.428013	-2.344011	2.08917
C	-3.985331	-0.441635	-0.763149	C	-3.830553	-0.775145	-0.81654
C	-3.993859	1.101905	-0.956768	C	-3.976722	0.767176	-0.971833

O	-5.109533	1.429958	-1.781516	O	-5.087961	1.013779	-1.829632
C	-4.152782	1.826621	0.349383	C	-4.249967	1.434159	0.345891
C	-3.250772	2.604459	0.965307	C	-3.437906	2.254079	1.028482
C	-1.86678	2.986624	0.511858	C	-2.058947	2.740741	0.665988
C	-1.637337	4.510282	0.552633	C	-1.893733	4.264244	0.847418
O	-4.093722	-1.079865	-2.044717	O	-3.852786	-1.385643	-2.115408
C	-0.215549	4.907792	0.140318	C	-2.747767	5.097774	-0.115069
O	2.441778	-1.76954	1.202973	O	2.669413	-1.541949	1.231594
C	3.325396	-2.891894	1.237232	C	3.618925	-2.608432	1.292968
H	0.654454	0.334964	-0.52392	H	0.714429	0.382928	-0.518617
H	1.885264	-2.998782	-1.488967	H	2.245097	-2.848322	-1.444902
H	0.836643	-0.541928	2.146163	H	0.966561	-0.47406	2.151114
H	2.936607	0.6778	1.417609	H	2.886911	0.98856	1.379077
H	4.315198	2.640738	1.995774	H	4.064834	3.086938	1.921154
H	6.00926	3.498519	0.390522	H	5.728617	4.036122	0.335677
H	6.318479	2.371257	-1.808126	H	6.209079	2.864245	-1.808322
H	4.934295	0.384893	-2.38319	H	5.025334	0.743574	-2.348852
H	-3.583918	-3.112673	2.353812	H	-3.346219	-1.795411	3.035391
H	-3.534956	-1.507074	3.081408	H	-4.451487	-2.233788	1.719822
H	-4.689891	-1.848048	1.77923	H	-3.253703	-3.402306	2.314597
H	-4.888499	-0.7227	-0.214893	H	-4.71529	-1.151822	-0.296264
H	-3.059972	1.385385	-1.460059	H	-3.055821	1.150733	-1.431159
H	-5.120543	0.779741	-2.507712	H	-5.016484	0.379991	-2.566903
H	-5.126282	1.680463	0.818444	H	-5.229734	1.19839	0.762447
H	-3.547683	3.037587	1.922856	H	-3.810522	2.629011	1.984657
H	-1.66651	2.615026	-0.500184	H	-1.804349	2.467928	-0.365797
H	-1.12819	2.501621	1.169016	H	-1.325725	2.229853	1.308055
H	-1.841855	4.877923	1.568439	H	-0.833675	4.517035	0.71066
H	-2.366724	5.001523	-0.105447	H	-2.140222	4.530752	1.885681
H	-3.20172	-1.257928	-2.390527	H	-2.940926	-1.475712	-2.442865
H	-0.078403	5.994815	0.18536	H	-2.593223	6.171093	0.048661
H	0.004596	4.587783	-0.886235	H	-3.816385	4.888182	0.011335
H	0.532234	4.447798	0.798964	H	-2.491828	4.879038	-1.159884
H	3.611944	-3.007963	2.283693	H	3.160197	-3.557521	0.995074
H	2.817489	-3.800885	0.896453	H	4.497672	-2.405056	0.666292
H	4.224663	-2.717075	0.631215	H	3.926508	-2.666578	2.338303

Conf. 7b-3	X axis(Å)	Y axis(Å)	Z axis(Å)	Conf. 7b-4	X axis(Å)	Y axis(Å)	Z axis(Å)
C	-0.556883	-1.307173	0.034634	C	-0.42893	-1.402044	0.063282
C	0.692105	-0.412214	0.223735	C	0.757668	-0.421316	0.230317
C	1.914565	-1.380032	-0.05855	C	2.043801	-1.321965	0.020546
N	1.304093	-2.486581	-0.756394	N	1.518358	-2.503334	-0.620624

C	-0.057517	-2.46867	-0.84896	C	0.160545	-2.574733	-0.744974
O	0.755735	0.239175	1.460224	O	0.75251	0.291917	1.434614
C	3.055741	-0.806386	-0.970988	C	3.168689	-0.734767	-0.903684
C	3.862493	0.378407	-0.568642	C	3.868442	0.53881	-0.580208
O	3.279382	-1.394112	-2.023469	O	3.475921	-1.389301	-1.893914
C	3.720912	1.041419	0.663651	C	3.63217	1.287624	0.586564
C	4.524738	2.14407	0.960013	C	4.336841	2.472045	0.811157
C	5.471286	2.596032	0.037773	C	5.27857	2.920383	-0.117802
C	5.616929	1.942859	-1.192787	C	5.518652	2.181283	-1.283204
C	4.820891	0.842509	-1.493128	C	4.820897	0.999903	-1.512175
O	-0.757271	-3.197392	-1.525348	O	-0.475989	-3.382088	-1.393702
O	-1.615755	-0.645769	-0.669812	O	-1.508846	-0.839416	-0.692387
C	-2.786786	-0.886991	0.001432	C	-2.676434	-1.096385	-0.021139
C	-2.642501	-1.538158	1.183467	C	-2.519512	-1.681952	1.192993
C	-1.219121	-1.795708	1.341907	C	-1.088203	-1.863551	1.382159
O	-0.613624	-2.285837	2.286057	O	-0.474545	-2.278384	2.356587
C	-3.669253	-1.940511	2.194935	C	-3.541169	-2.074828	2.213292
C	-3.998292	-0.432713	-0.750874	C	-3.896278	-0.714423	-0.799899
C	-4.044165	1.098853	-1.009146	C	-3.967051	0.801055	-1.143793
O	-5.151197	1.358507	-1.872495	O	-5.087462	0.998968	-2.003202
C	-4.253693	1.871484	0.262375	C	-4.169815	1.641863	0.084165
C	-3.53345	2.909283	0.711973	C	-3.308723	2.51221	0.632323
C	-2.341388	3.577936	0.082338	C	-1.932325	2.895291	0.15552
C	-1.114323	3.628557	1.014473	C	-1.743878	4.417865	-0.036295
O	-4.086183	-1.126878	-2.005106	O	-3.970049	-1.479054	-2.013439
C	0.05895	4.402911	0.403106	C	-1.811568	5.238652	1.258097
O	2.439129	-1.724375	1.212625	O	2.570195	-1.555698	1.317169
C	3.315676	-2.851655	1.260142	C	3.484886	-2.645262	1.453582
H	0.663403	0.366747	-0.544052	H	0.696635	0.31697	-0.574689
H	1.839983	-2.999475	-1.450138	H	2.103299	-3.022313	-1.268872
H	0.854798	-0.449736	2.145102	H	0.897805	-0.35366	2.152603
H	2.985659	0.705719	1.382877	H	2.899924	0.955533	1.310949
H	4.408527	2.649696	1.914804	H	4.147889	3.043602	1.715832
H	6.094062	3.455248	0.27399	H	5.824589	3.842979	0.062707
H	6.350429	2.293803	-1.913724	H	6.24872	2.528618	-2.00936
H	4.92278	0.325147	-2.441572	H	4.996033	0.416075	-2.409978
H	-3.574456	-3.006252	2.432536	H	-3.405935	-3.12228	2.506229
H	-3.529248	-1.388558	3.132316	H	-3.435092	-1.470321	3.122295
H	-4.687077	-1.756114	1.839658	H	-4.56119	-1.950922	1.838728
H	-4.896963	-0.711618	-0.193548	H	-4.790664	-0.978057	-0.22906
H	-3.108564	1.391258	-1.503447	H	-3.041434	1.07358	-1.667515
H	-5.129933	0.671399	-2.564252	H	-5.064328	0.279038	-2.66003
H	-5.120185	1.552497	0.843037	H	-5.14396	1.502813	0.55426

H	-3.851194	3.354336	1.657349	H	-3.640537	3.016285	1.540543
H	-2.625971	4.611385	-0.172819	H	-1.697773	2.389128	-0.78726
H	-2.066464	3.095222	-0.862589	H	-1.190413	2.539459	0.887498
H	-0.797308	2.606634	1.258138	H	-2.495328	4.785502	-0.748121
H	-1.404771	4.09687	1.966095	H	-0.764768	4.579055	-0.507892
H	-3.189873	-1.331242	-2.324244	H	-3.069557	-1.651985	-2.339372
H	0.920445	4.417047	1.080949	H	-1.609982	6.297837	1.058245
H	-0.217833	5.443797	0.190459	H	-1.068078	4.889582	1.986716
H	0.387534	3.948866	-0.540743	H	-2.797848	5.179942	1.733008
H	2.79996	-3.763056	0.937887	H	3.783421	-2.647055	2.503156
H	4.212048	-2.692638	0.645505	H	2.998638	-3.596719	1.211999
H	3.608351	-2.952131	2.306508	H	4.37389	-2.509456	0.822996

Conf. 7b-5	X axis(Å)	Y axis(Å)	Z axis(Å)	Conf. 7b-6	X axis(Å)	Y axis(Å)	Z axis(Å)
C	0.016569	-0.963154	0.236229	C	-0.131879	-0.851656	0.23948
C	-1.374853	-0.655633	-0.36984	C	-1.547428	-0.583513	-0.327839
C	-2.265177	-0.246597	0.87571	C	-2.367963	-0.026636	0.907917
N	-1.288345	0.046468	1.895454	N	-1.337578	0.353812	1.842216
C	0.014889	-0.22291	1.588011	C	-0.05521	0.029489	1.502042
O	-1.912805	-1.695657	-1.136027	O	-2.138615	-1.687712	-0.95156
C	-3.177218	1.018928	0.706666	C	-3.268382	1.231255	0.646259
C	-4.253375	1.082015	-0.320879	C	-4.390934	1.20555	-0.332033
O	-2.996042	1.947727	1.486885	O	-3.035642	2.234989	1.311571
C	-4.546898	0.033898	-1.211297	C	-4.756296	0.068054	-1.073737
C	-5.572649	0.179945	-2.147688	C	-5.82328	0.133602	-1.972348
C	-6.314133	1.362162	-2.204467	C	-6.534305	1.32415	-2.138905
C	-6.02766	2.410955	-1.32118	C	-6.176083	2.461801	-1.404257
C	-5.006035	2.27243	-0.387294	C	-5.113853	2.403172	-0.508042
O	0.996148	0.088336	2.235831	O	0.961311	0.390196	2.064169
O	1.089334	-0.443038	-0.556476	O	0.907145	-0.441745	-0.655835
C	2.023896	-1.430399	-0.705056	C	1.821649	-1.455808	-0.736476
C	1.666291	-2.640589	-0.198445	C	1.472565	-2.596538	-0.083768
C	0.347001	-2.464759	0.383134	C	0.184538	-2.334133	0.534218
O	-0.407219	-3.288454	0.888751	O	-0.555574	-3.083842	1.161328
C	2.397559	-3.950694	-0.192584	C	2.186581	-3.910582	0.035884
C	3.272477	-0.922957	-1.383601	C	3.044927	-1.045857	-1.519317
C	4.177052	-0.197008	-0.34614	C	3.986913	-0.203451	-0.611186
O	4.815175	-1.280595	0.368691	O	4.63757	-1.195051	0.215225
C	5.196896	0.690786	-1.002348	C	4.995793	0.583116	-1.399863
C	5.466648	1.966474	-0.679698	C	5.221253	1.904046	-1.312213
C	4.821789	2.833898	0.368632	C	4.532253	2.930223	-0.450086
C	4.12712	4.073053	-0.233925	C	5.516305	3.764787	0.398458

O	3.984017	-1.937539	-2.060962	O	3.728567	-2.141884	-2.090206
C	3.54524	5.003131	0.836487	C	6.243172	2.954054	1.477533
O	-3.078049	-1.374549	1.162378	O	-3.182893	-1.100507	1.353823
C	-3.665564	-1.4294	2.462818	C	-3.699355	-1.005959	2.681911
H	-1.266854	0.20542	-1.03623	H	-1.460294	0.199691	-1.086921
H	-1.498744	0.738139	2.608458	H	-1.502477	1.121801	2.485736
H	-2.065988	-2.448181	-0.532762	H	-2.271122	-2.36971	-0.265325
H	-3.974118	-0.884362	-1.183533	H	-4.20795	-0.858224	-0.959571
H	-5.790408	-0.634457	-2.83336	H	-6.097142	-0.75006	-2.542397
H	-7.112497	1.469379	-2.934656	H	-7.364743	1.368677	-2.839211
H	-6.601267	3.333041	-1.364167	H	-6.725599	3.390489	-1.533383
H	-4.771453	3.076278	0.302943	H	-4.823671	3.275987	0.067815
H	3.320564	-3.890282	0.395706	H	3.125282	-3.805052	0.592353
H	1.75653	-4.712412	0.262268	H	1.54816	-4.612205	0.581954
H	2.65821	-4.276375	-1.204742	H	2.416831	-4.340618	-0.943987
H	2.957097	-0.190578	-2.134887	H	2.704532	-0.410435	-2.344258
H	3.53494	0.36209	0.341393	H	3.368974	0.446106	0.016082
H	5.539878	-0.916458	0.900112	H	5.348626	-0.765614	0.715408
H	5.767475	0.218274	-1.801581	H	5.592624	-0.007664	-2.094506
H	6.250043	2.463222	-1.25619	H	6.005769	2.311691	-1.954198
H	5.60384	3.180186	1.062586	H	3.785621	2.46911	0.206443
H	4.099649	2.272117	0.972053	H	3.980205	3.616015	-1.110777
H	3.329408	3.741028	-0.911854	H	4.95621	4.581941	0.871561
H	4.847686	4.62647	-0.852938	H	6.252733	4.239592	-0.265927
H	4.580661	-2.342884	-1.405082	H	4.342179	-2.473223	-1.409159
H	3.053776	5.871305	0.38168	H	6.914099	3.591602	2.065318
H	4.329861	5.376832	1.506916	H	6.848615	2.152568	1.036574
H	2.801057	4.482797	1.452198	H	5.528918	2.493546	2.172557
H	-4.252418	-2.349312	2.480458	H	-4.305128	-1.902367	2.824912
H	-2.893956	-1.47546	3.239259	H	-2.887681	-0.994433	3.417715
H	-4.32823	-0.572377	2.645732	H	-4.331352	-0.116437	2.809783

Table S5 DP4+ probability analysis of compound **1a.**

Functional		Solvent?		Basis Set		Type of Data	
mPW1PW91		PCM		6-31+G(d,p)		Shielding Tensors	
		DP4+	100.00%	0.00%	-	-	-
**Nuclei**	**sp2?**	**xperimenta**	**Isomer 1**	**Isomer 2**	**Isomer 3**	**Isomer 4**	**Isomer 5**
C	x	128.1	68.696187	68.610927			
C	x	134.6	63.149925	64.374212			
C	x	114.9	81.241776	82.390394			
C	x	142.3	56.883976	56.263626			
C	x	122.7	75.293985	74.457137			
C	x	109.6	91.069051	91.727528			
C	x	160.3	38.151679	38.147949			
C	x	94.8	100.77355	102.1278			
C	x	138.5	60.221499	59.319637			
C	x	141.9	54.513557	54.955497			
C	x	113.3	82.479926	82.781732			
C	x	166.7	32.316834	33.867284			
C	x	119.5	78.15785	76.2045			
C		55.3	141.06121	141.2367			
C		59.6	131.60502	130.93646			
C		28.3	164.03429	160.7891			
C		25.2	167.78942	172.05316			
C		49.5	143.68487	145.13803			
C	x	141.1	49.782221	51.834293			
C		20.2	167.55664	167.97163			
C		27.1	175.10299	174.79247			
C	x	172.6	21.956767	22.106332			
C		51.6	143.15113	143.14681			
H		11.75	23.300178	23.492936			
H	x	8.17	23.004962	22.816854			
H	x	6.88	24.388194	24.282319			
H	x	7.04	24.215179	24.213443			
H	x	8.33	22.576726	22.303913			
H	x	6.79	24.535474	24.678212			
H		3.88	27.610338	27.56437			
H		3.88	27.602271	27.562895			
H		3.88	27.366184	27.374408			
H		4.55	26.849862	25.885967			
H		2.25	29.164588	29.096674			
H		1.87	29.543833	29.333223			
H		1.89	29.28754	29.615029			
H		1.89	29.504463	29.589748			
H		3.89	27.215012	27.784882			
H		4.01	27.330553	27.571975			
H		2.19	29.375931	29.457411			
H		2.19	29.390041	29.404384			
H		2.19	29.399801	29.370211			
H		2.06	28.958338	29.817098			
H		2.06	29.467512	29.248691			
H		2.06	29.282809	29.593292			
H		3.67	27.755986	27.90077			
H		3.67	27.758294	28.024424			
H		3.67	27.722865	27.891192			

Functional	Solvent?	Basis Set	Type of Data
mPW1PW91	PCM	6-31+G(d,p)	Shielding Tensors

	Isomer 1	Isomer 2	Isomer 3	Isomer 4	Isomer 5	Isomer 6
sDP4+ (H data)	99.56%	0.44%	-	-	-	-
sDP4+ (C data)	96.95%	3.05%	-	-	-	-
sDP4+ (all data)	99.99%	0.01%	-	-	-	-
uDP4+ (H data)	72.11%	27.89%	-	-	-	-
uDP4+ (C data)	98.89%	1.11%	-	-	-	-
uDP4+ (all data)	99.57%	0.43%	-	-	-	-
DP4+ (H data)	99.83%	0.17%	-	-	-	-
DP4+ (C data)	99.96%	0.04%	-	-	-	-
DP4+ (all data)	100.00%	0.00%	-	-	-	-

Table S6. DP4+ probability analysis of compound **1b**

Functional		Solvent?		Basis Set		Type of Data	
mPW1PW91		PCM		6-31+G(d,p)		Shielding Tensors	
		DP4+	0.00%	100.00%	-	-	-
Nuclei	sp2?	xperimenta	Isomer 1	Isomer 2	Isomer 3	Isomer 4	Isomer 5
C	x	128.3	68.6961873	68.6109273			
C	x	134.4	63.1499247	64.3742116			
C	x	114.9	81.2417762	82.3903943			
C	x	142.5	56.8839757	56.2636256			
C	x	122.7	75.2939851	74.4571373			
C	x	109.6	91.0690507	91.7275278			
C	x	160.3	38.1516788	38.1479486			
C	x	94.8	100.773546	102.1278			
C	x	138.2	60.2214987	59.3196371			
C	x	141.8	54.5135575	54.9554967			
C	x	113.4	82.4799258	82.7817325			
C	x	166.2	32.3168345	33.8672844			
C	x	120	78.1578498	76.2045			
C		55.3	141.061214	141.236703			
C		60.7	131.605021	130.936461			
C		31.3	164.034294	160.7891			
C		21.6	167.789416	172.053161			
C		47.8	143.684869	145.13803			
C	x	140.4	49.7822213	51.834293			
C		19.9	167.556636	167.971628			
C		26.5	175.102985	174.792466			
C	x	172.9	21.956767	22.1063318			
C		51.4	143.15113	143.146812			
H		11.55	23.3001778	23.4929364			
H	x	8.17	23.0049622	22.8168538			
H	x	6.87	24.3881935	24.2823191			
H	x	7.04	24.2151792	24.2134434			
H	x	8.43	22.576726	22.3039125			
H	x	6.59	24.5354738	24.6782118			
H		3.87	27.6103376	27.5643695			
H		3.87	27.6022711	27.5628948			
H		3.87	27.3661844	27.3744084			
H		5.21	26.8498616	25.8859672			
H		2.28	29.1645881	29.096674			
H		1.97	29.5438328	29.3332235			
H		1.83	29.28754	29.6150288			
H		1.83	29.5044628	29.5897475			
H		3.69	27.2150119	27.7848822			
H		3.69	27.330553	27.5719746			
H		1.91	29.3759312	29.4574115			
H		1.91	29.3900407	29.404384			
H		1.91	29.3998011	29.3702109			
H		2.03	28.9583379	29.8170976			
H		2.03	29.4675123	29.2486909			
H		2.03	29.2828089	29.5932925			
H		3.46	27.7559859	27.9007698			
H		3.46	27.7582935	28.0244238			
H		3.46	27.7228653	27.8911917			

Functional		Solvent?		Basis Set		Type of Data	
mPW1PW91		PCM		6-31+G(d,p)		Shielding Tensors	
	Isomer 1	Isomer 2	Isomer 3	Isomer 4	Isomer 5	Isomer 6	
sDP4+ (H data)	0.00%	100.00%	–	–	–	–	
sDP4+ (C data)	0.26%	99.74%	–	–	–	–	
sDP4+ (all data)	0.00%	100.00%	–	–	–	–	
uDP4+ (H data)	0.02%	99.98%	–	–	–	–	
uDP4+ (C data)	0.29%	99.71%	–	–	–	–	
uDP4+ (all data)	0.00%	100.00%	–	–	–	–	
DP4+ (H data)	0.00%	100.00%	–	–	–	–	
DP4+ (C data)	0.00%	100.00%	–	–	–	–	
DP4+ (all data)	0.00%	100.00%	–	–	–	–	

Table S7. DP4+ probability analysis of compound **7.**

Functional		Solvent?		Basis Set		Type of Data	
mPW1PW91		PCM		6-31+G(d,p)		Shielding Tensors	
		DP4+	100.00%	0.00%	-	-	-
Nuclei	sp2?	xperimenta	Isomer 1	Isomer 2	Isomer 3	Isomer 4	Isomer 5
C		91.1	101.430164	99.9309415			
C		74.9	119.133947	119.452039			
C		92.4	105.179152	103.192169			
C	x	166.5	33.2918413	30.7774664			
C	x	196.3	1.89352267	2.31874554			
C	x	133.4	66.50782	66.2249206			
C	x	130.2	63.4605916	62.8861971			
C	x	128.3	69.6808631	70.2998307			
C	x	133.7	62.0863358	60.2749689			
C	x	128.3	68.0314773	72.3171495			
C	x	130.2	67.277268	62.7786561			
C	x	186.9	9.05045029	5.97530612			
C	x	111.5	82.5380389	85.3230747			
C	x	196.7	1.31648299	2.49274938			
C		5.6	188.663258	187.973855			
C		71.9	122.180396	122.454146			
C		68.3	124.723411	123.102124			
C	x	129.8	71.7265826	70.1096391			
C	x	131.9	57.7494436	61.0857372			
C		29.2	163.186966	163.883029			
C		22.2	169.944114	171.104124			
C		13.5	181.03406	181.821077			
C		51.6	144.495337	144.367853			
H		4.4	26.8918503	26.6435514			
H	x	8.25	22.2173312	22.2297111			
H	x	7.53	23.6619316	23.5714441			
H	x	7.67	23.3758739	23.5237246			
H	x	7.53	23.5890035	23.4205045			
H	x	8.25	22.7057917	22.7671522			
H		1.64	29.7987648	29.8149259			
H		1.64	29.7621518	29.8037448			
H		1.64	29.6849628	29.8299236			
H		4.34	27.1299255	26.7944493			
H		4.45	26.6775897	26.6975652			
H	x	5.41	25.7236688	25.2955444			
H	x	5.43	25.4276015	25.4517071			
H		1.99	29.5812925	29.3108829			
H		1.99	29.4947758	29.2799105			
H		1.3	30.3012988	30.0476043			
H		1.3	30.2334978	29.9762157			
H		0.83	30.5803041	30.5347231			
H		0.83	30.8330007	30.8392104			
H		0.83	30.8965675	30.6496093			
H		3.25	27.8331797	27.8398647			
H		3.25	28.0193399	27.8019527			
H		3.25	28.4299435	28.5476358			

Functional	Solvent?	Basis Set	Type of Data
mPW1PW91	PCM	6-31+G(d,p)	Shielding Tensors

	Isomer 1	Isomer 2	Isomer 3	Isomer 4	Isomer 5	Isomer 6
sDP4+ (H data)	88.97%	11.03%	–	–	–	–
sDP4+ (C data)	99.17%	0.83%	–	–	–	–
sDP4+ (all data)	99.90%	0.10%	–	–	–	–
uDP4+ (H data)	100.00%	0.00%	–	–	–	–
uDP4+ (C data)	95.48%	4.52%	–	–	–	–
uDP4+ (all data)	100.00%	0.00%	–	–	–	–
DP4+ (H data)	100.00%	0.00%	–	–	–	–
DP4+ (C data)	99.96%	0.04%	–	–	–	–
DP4+ (all data)	100.00%	0.00%	–	–	–	–

Figure **S1**. Chiral HPLC analysis of the acidic hydrolysate of compounds **1a/1b**.

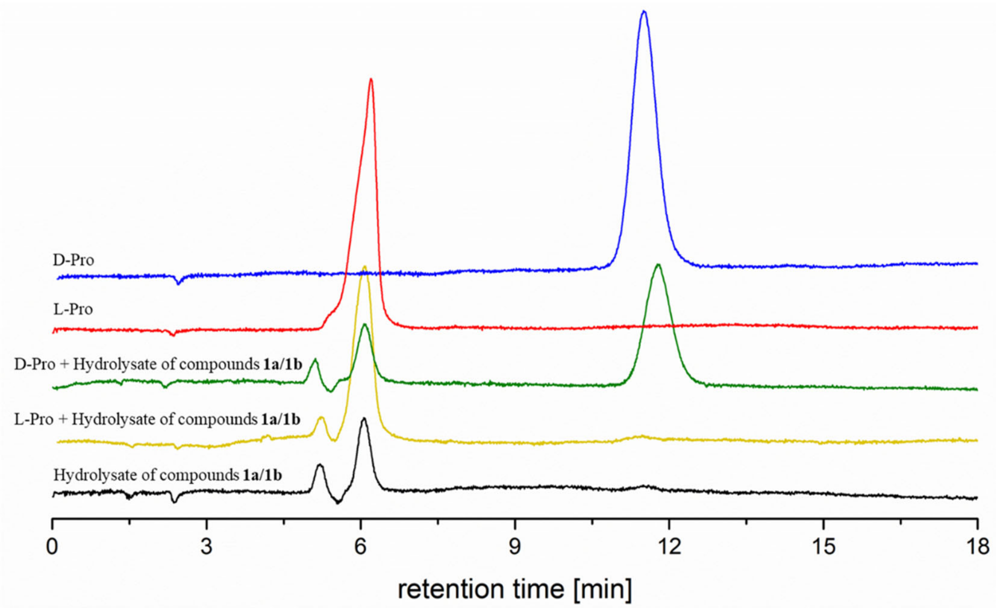

Figure S2. ^{1}H NMR (500 MHz, DMSO-d_6) spectrum of compounds **1a/1b.**

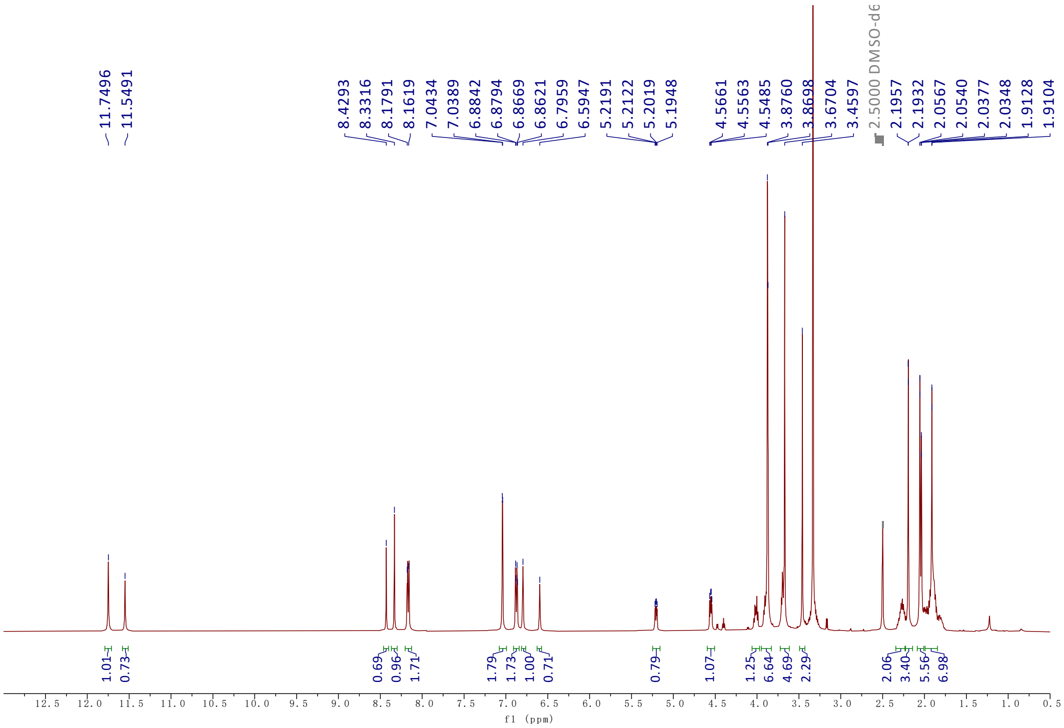

Figure S3. ^{13}C NMR (125 MHz, DMSO-d_6) and DEPT spectra of compounds **1a/1b**.

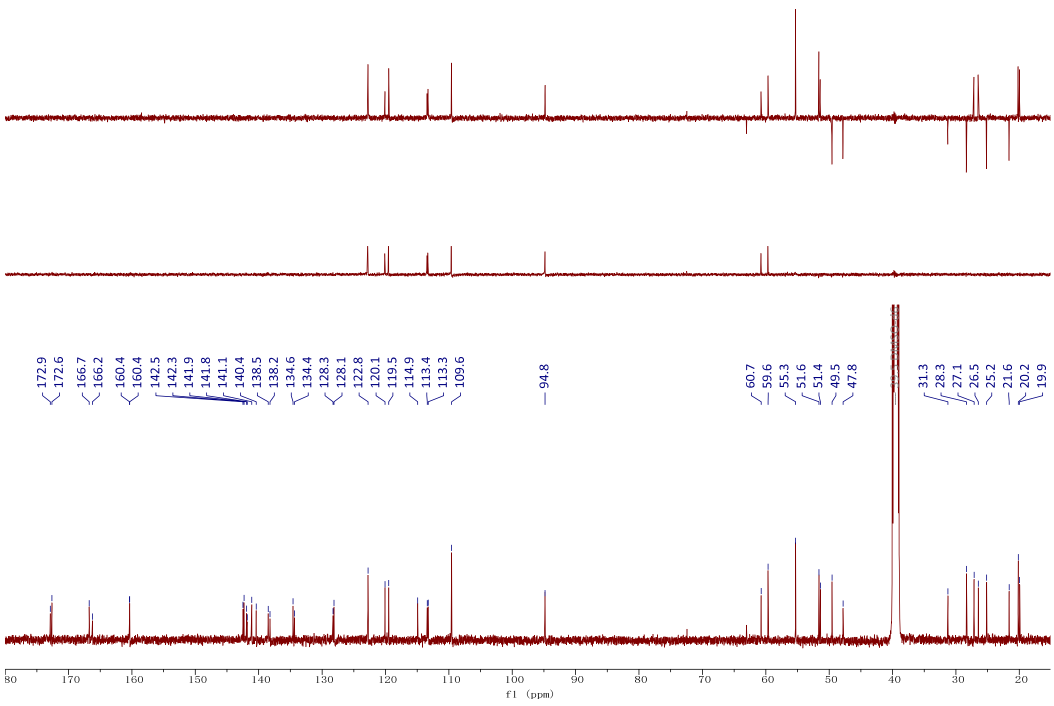

Figure S4. ^1H–^1H COSY spectrum of compounds **1a/1b**.

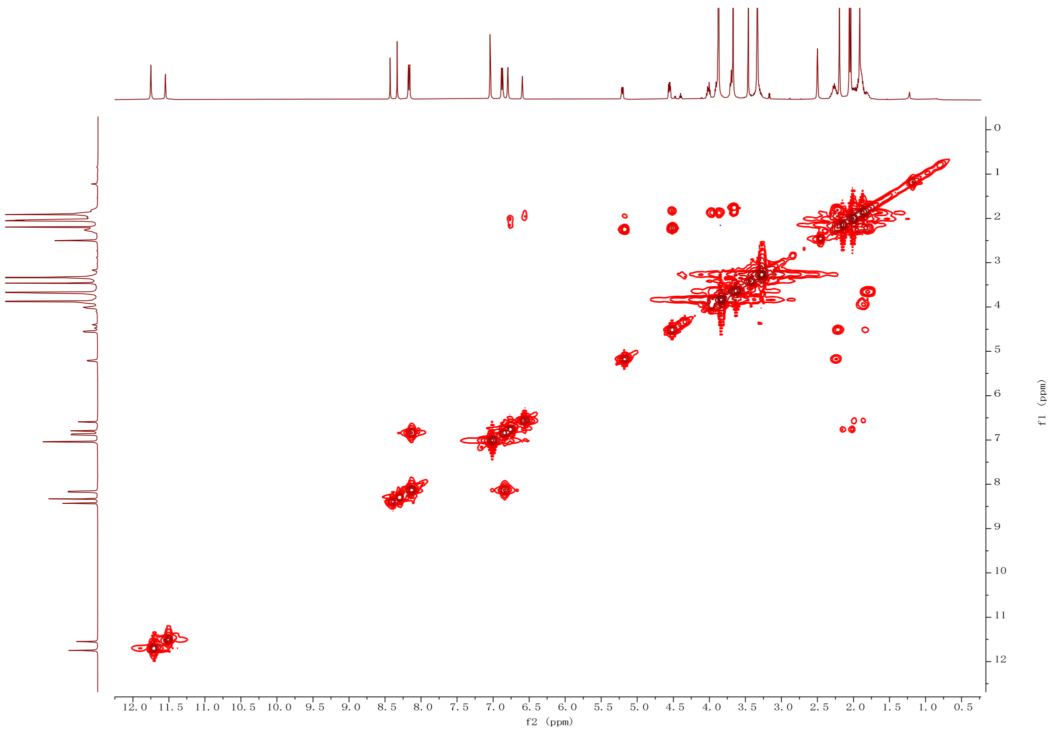

Figure S5. HSQC spectrum of compounds **1a/1b.**

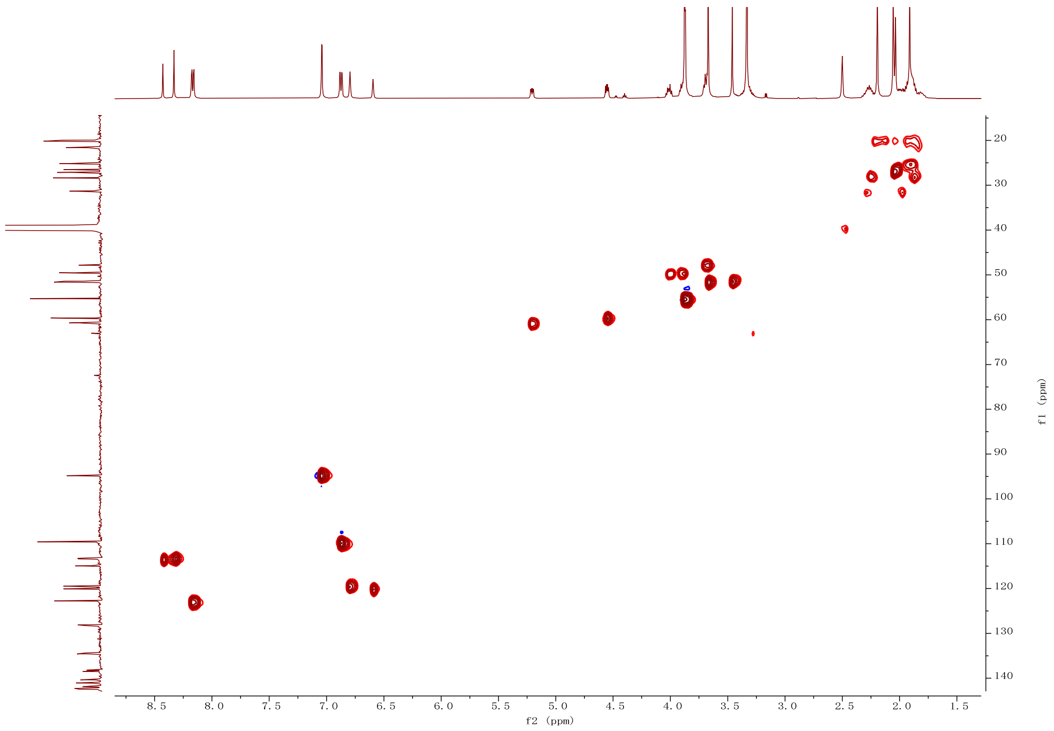

Figure S6. HMBC spectrum of compounds **1a/1b**.

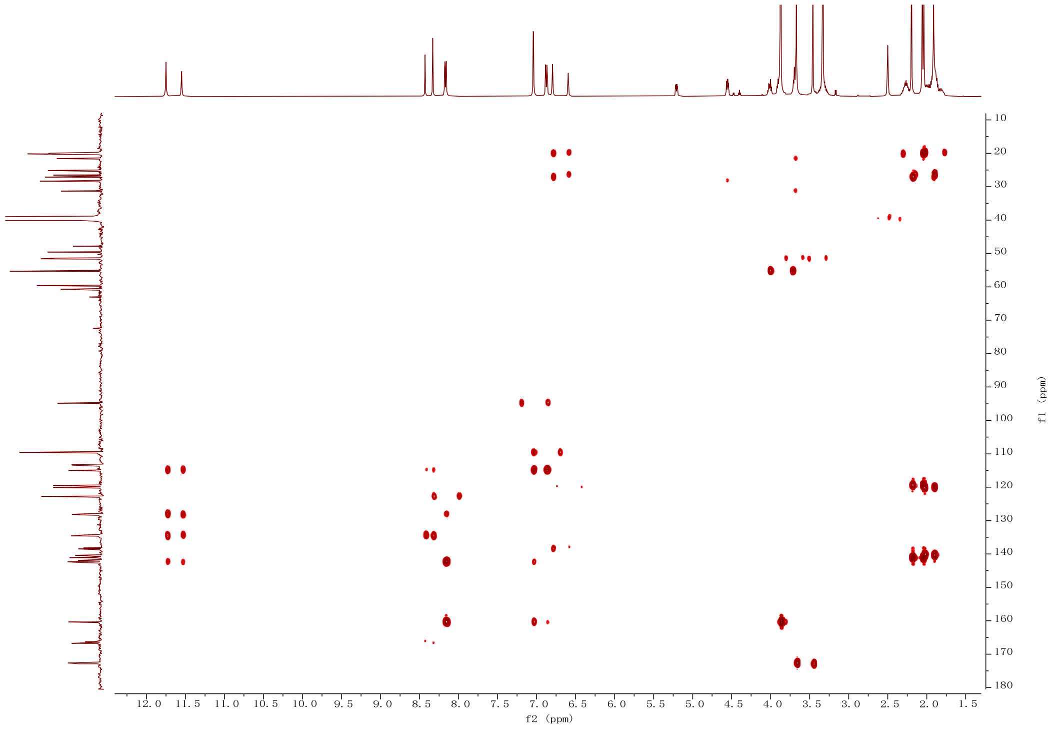

Figure S7. NOESY spectrum of compounds **1a/1b**.

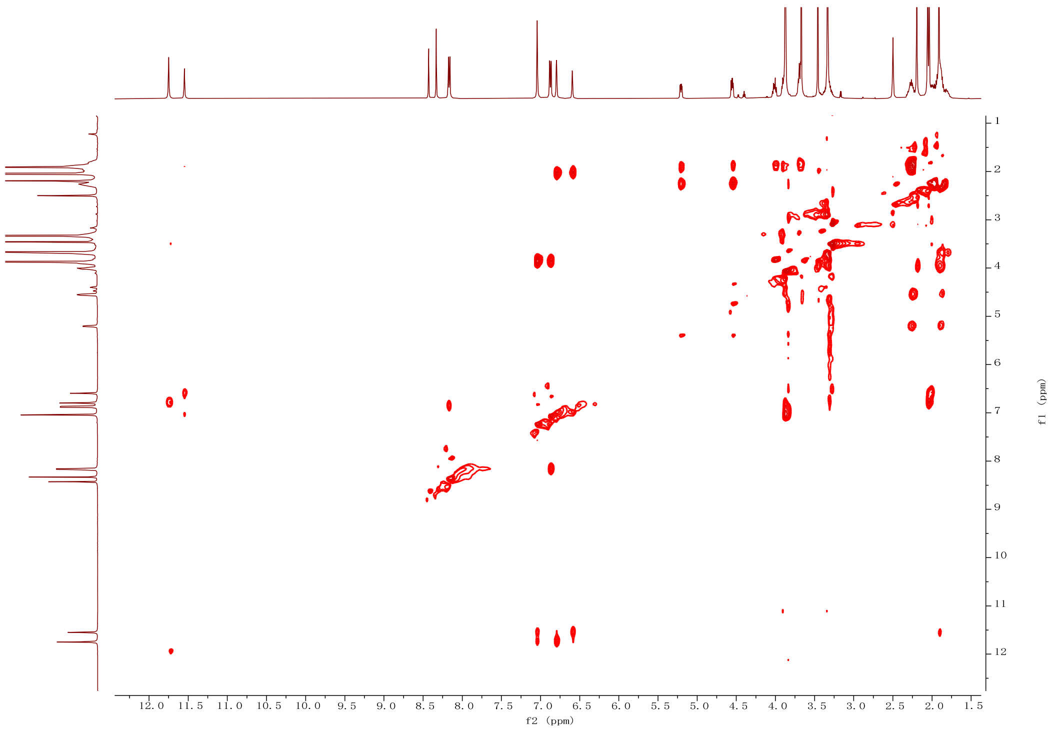

Figure S8. HR-ESI-MS spectrum of compounds **1a/1b**.

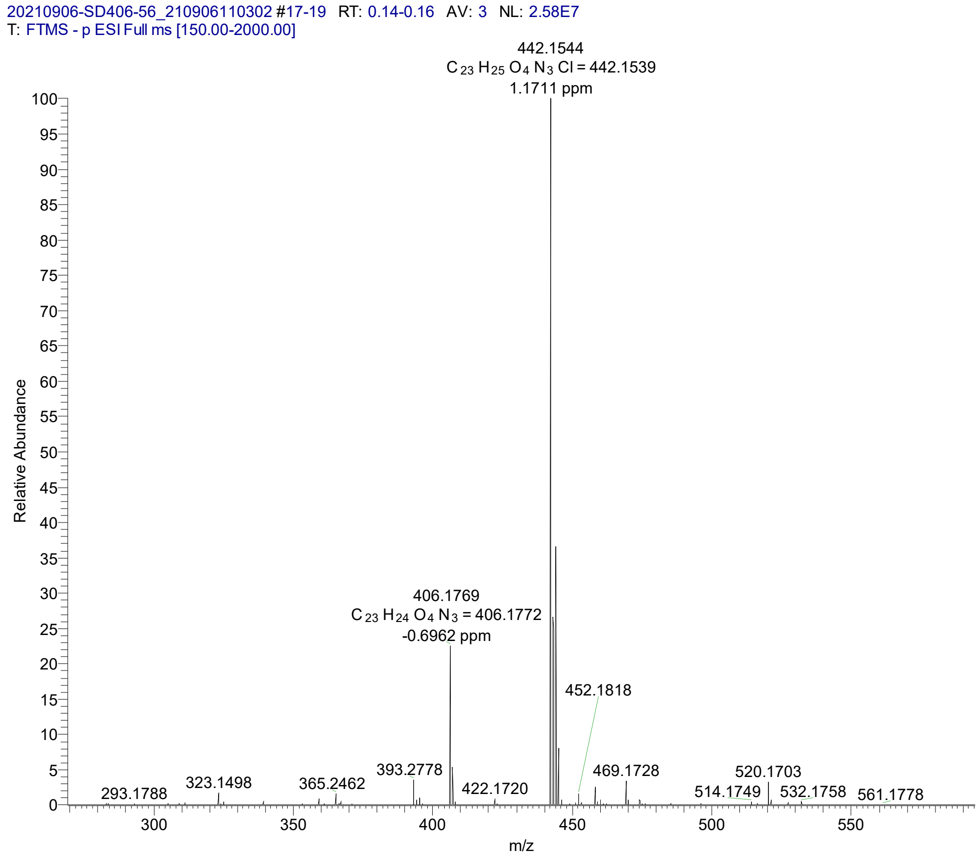

Figure **S9**. Optimized geometries of predominant conformers (weighting factors) for **isomer 1** at the B3LYP/6-311g(d,p) level above 2% population.

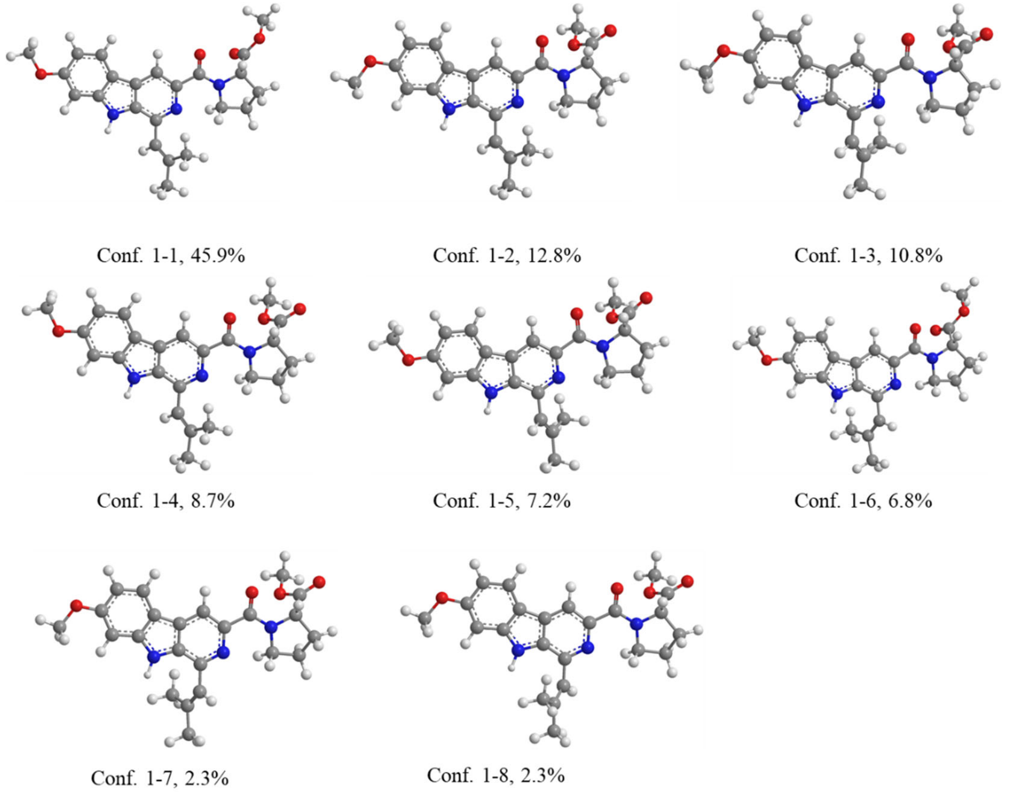

Figure **S10**. Optimized geometries of predominant conformers (weighting factors) for **isomer 2** at the B3LYP/6-311g(d,p) level above 2% population.

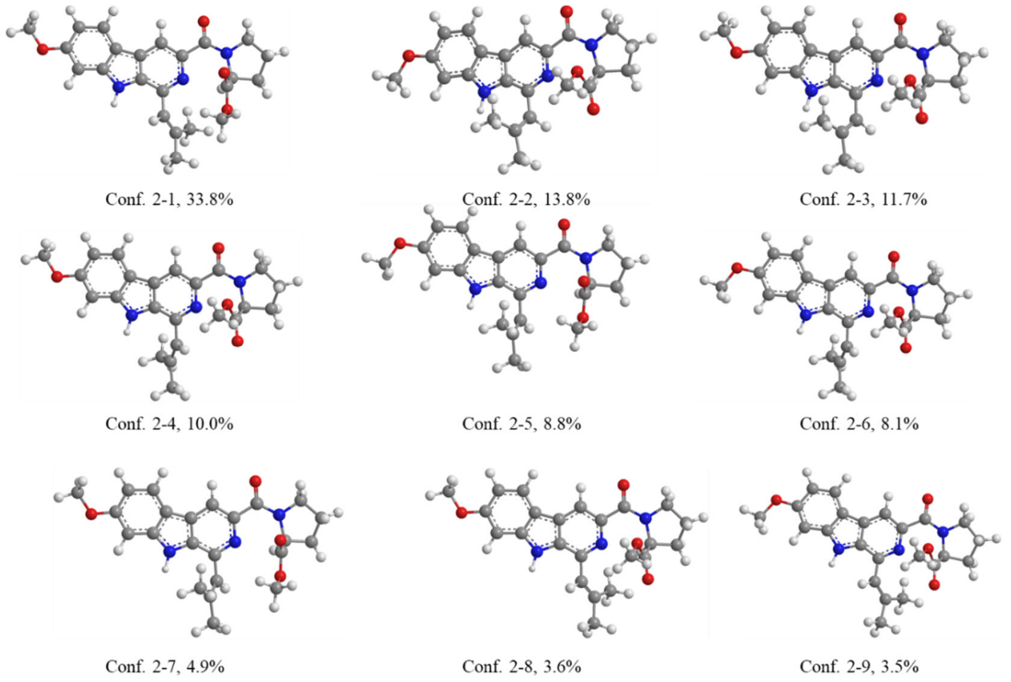

Figure S11. ^{1}H-NMR (500 MHz, DMSO-d_6) spectrum of compound **6**.

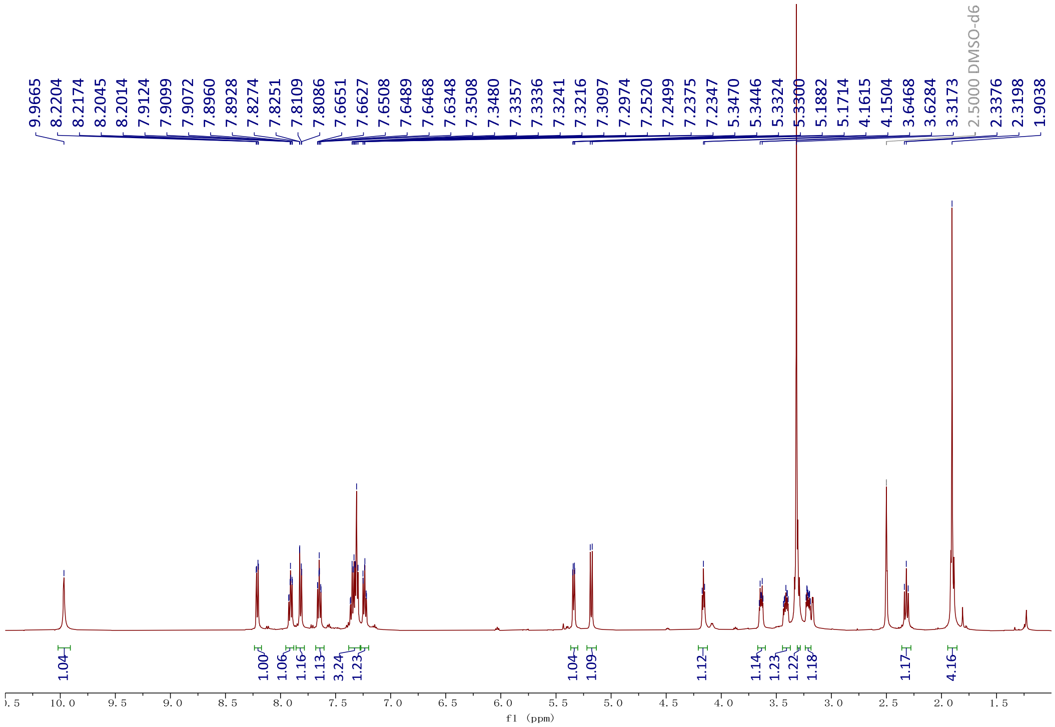

Figure S12. ^{13}C NMR (125 MHz, DMSO-d_6) and DEPT spectra of compound **6**.

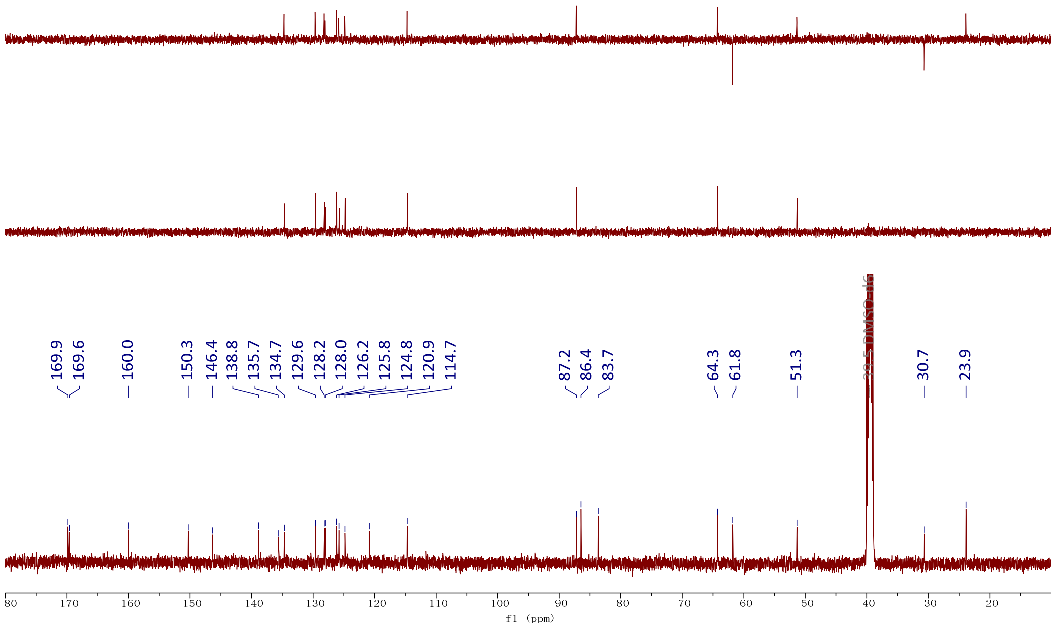

Figure S13. ^{1}H–^{1}H COSY spectrum of compound **6.**

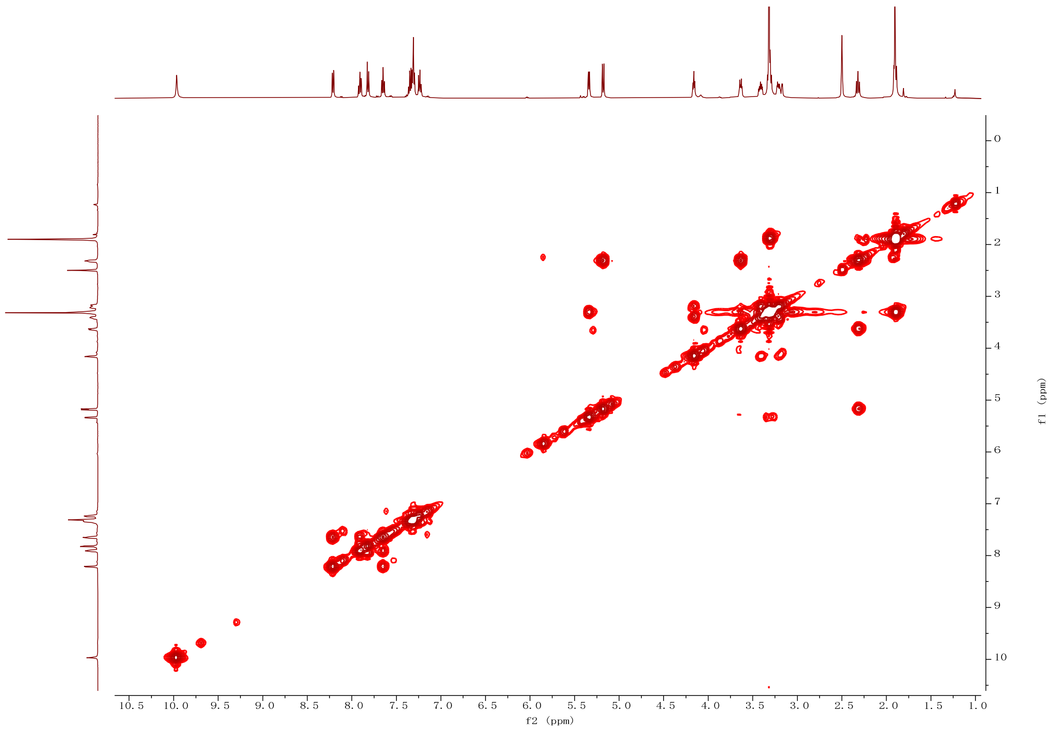

Figure S14. HSQC spectrum of compound **6**.

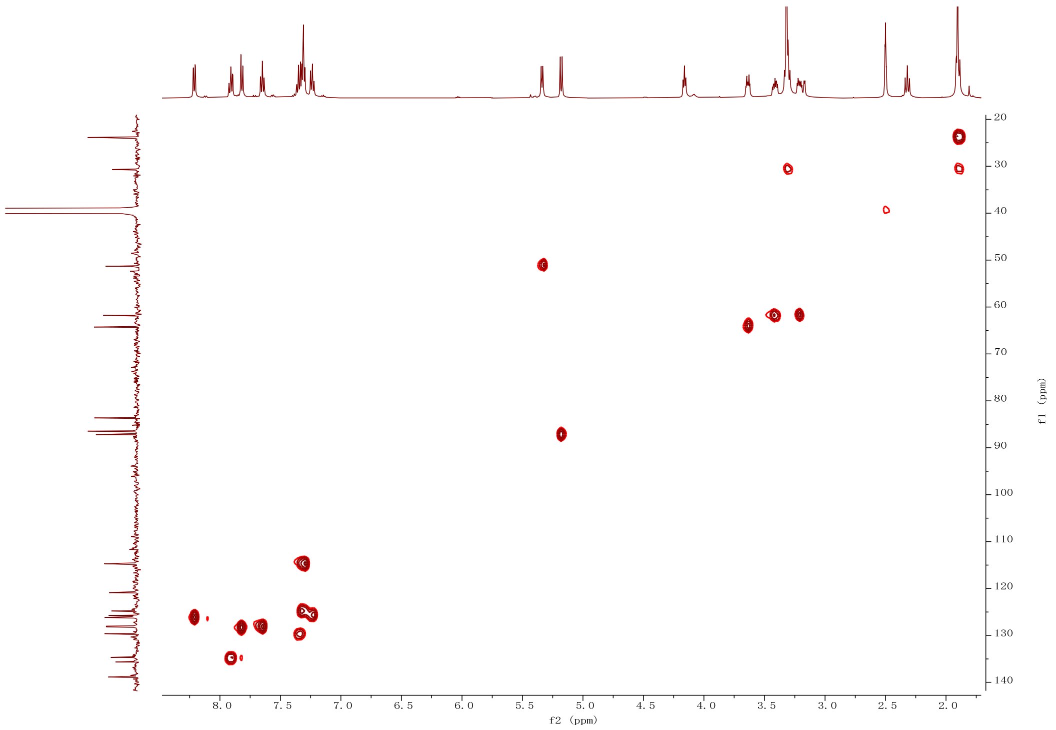

Figure S15. HMBC spectrum of compound **6**.

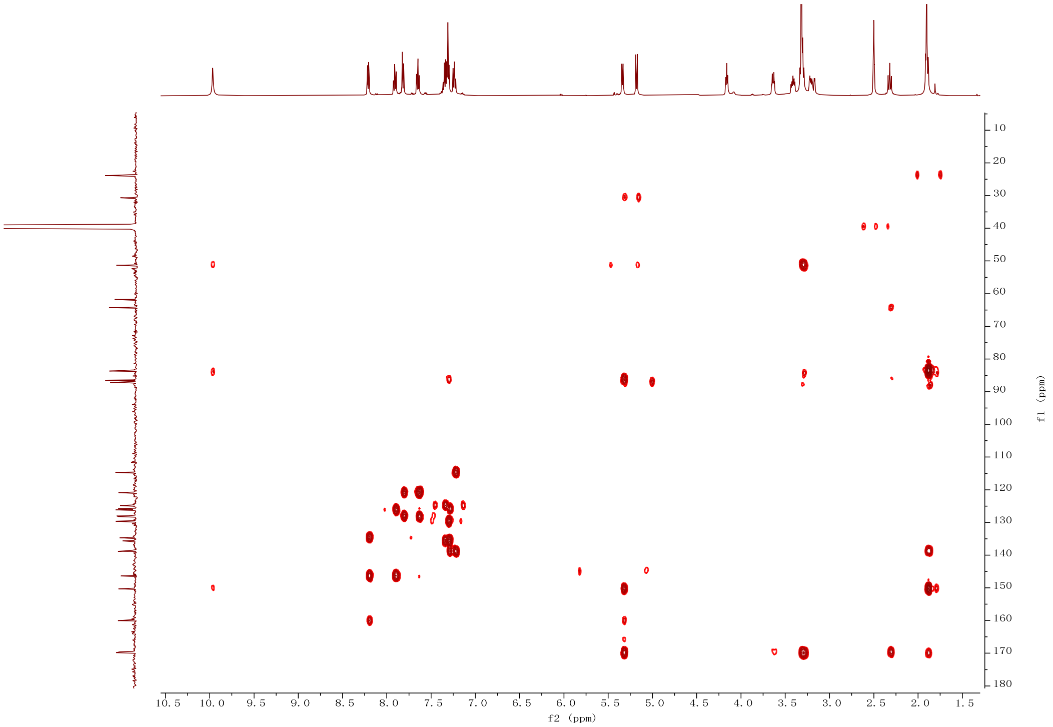

Figure S16. NOESY spectrum of compound **6**.

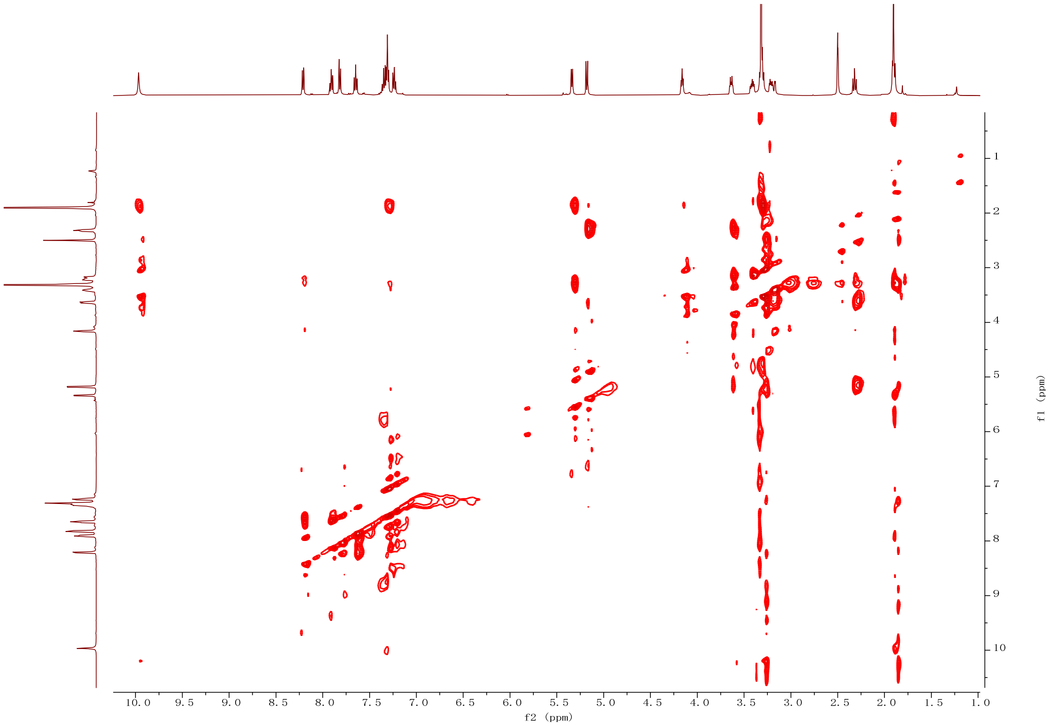

Figure S17. HR-ESI-MS spectrum of compound **6**.

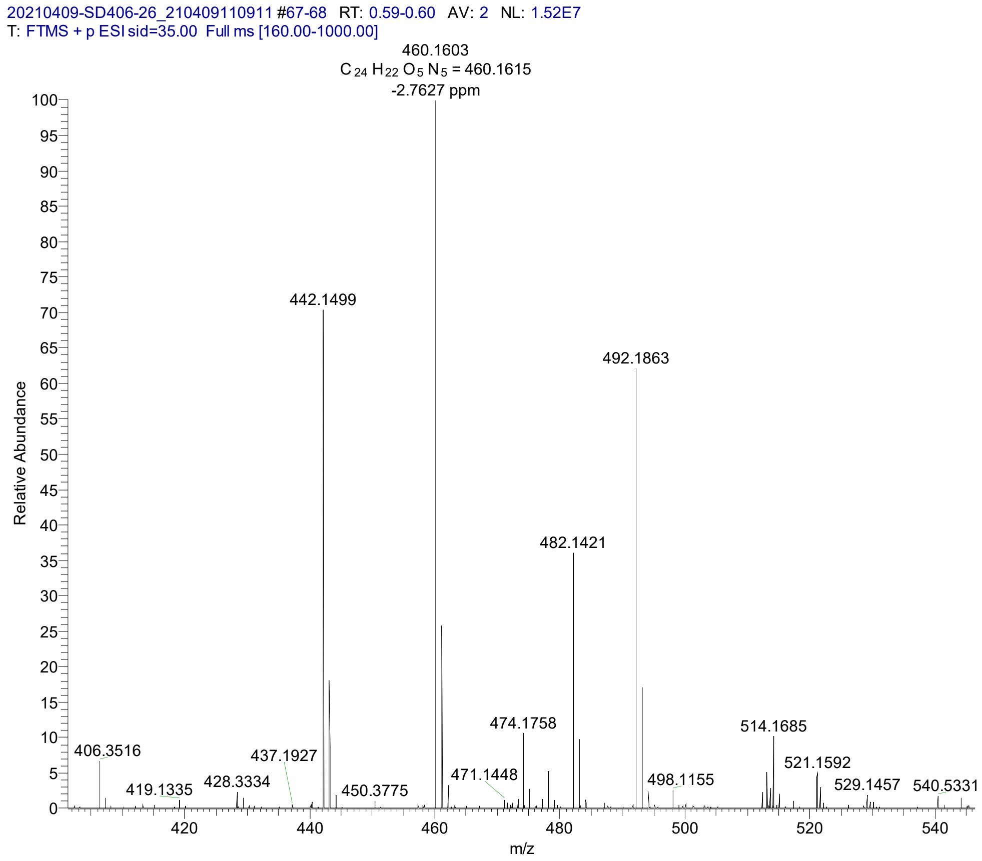

Figure S18. ECD spectrum of compound **6.**

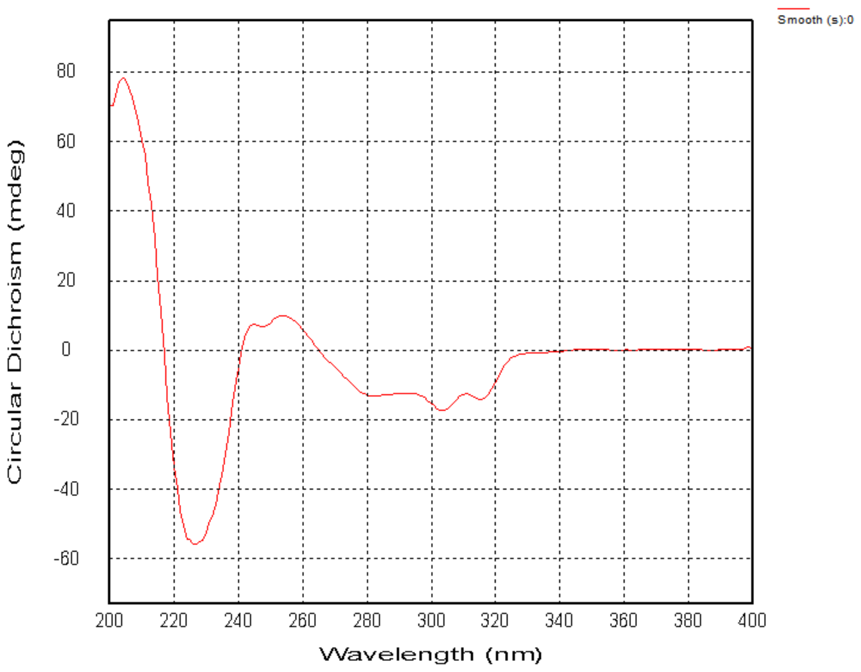

Figure S19. ^{1}H-NMR (500 MHz, DMSO-d_6) spectrum of compound **7.**

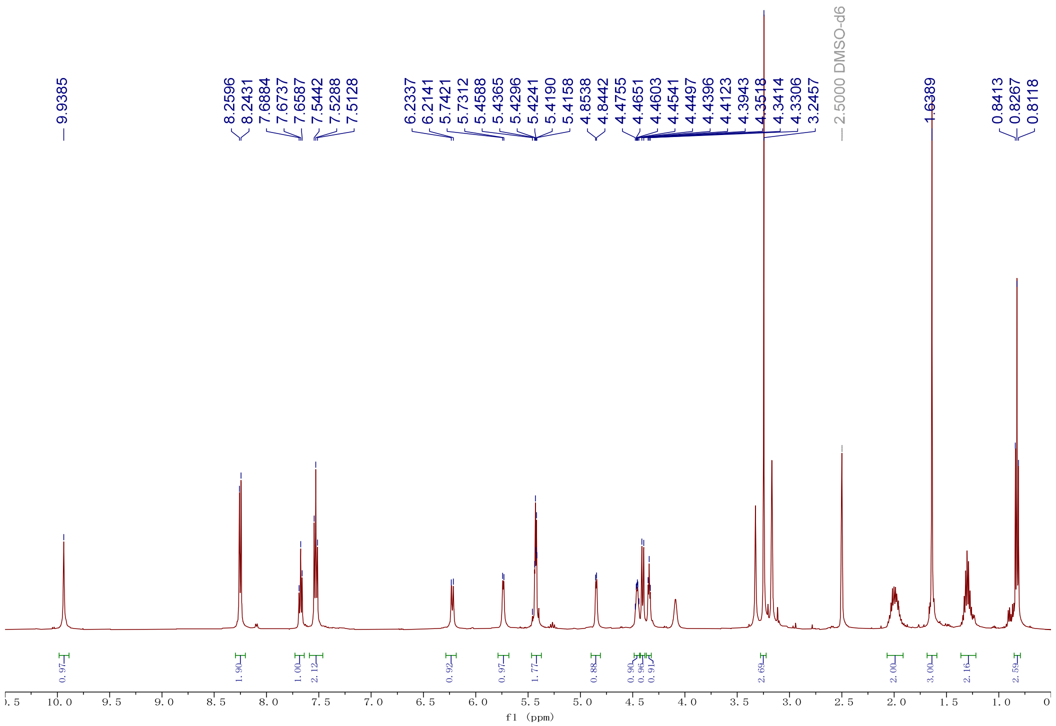

Figure S20. ^{13}C NMR (125 MHz, DMSO-d_6) and DEPT spectra of compound **7.**

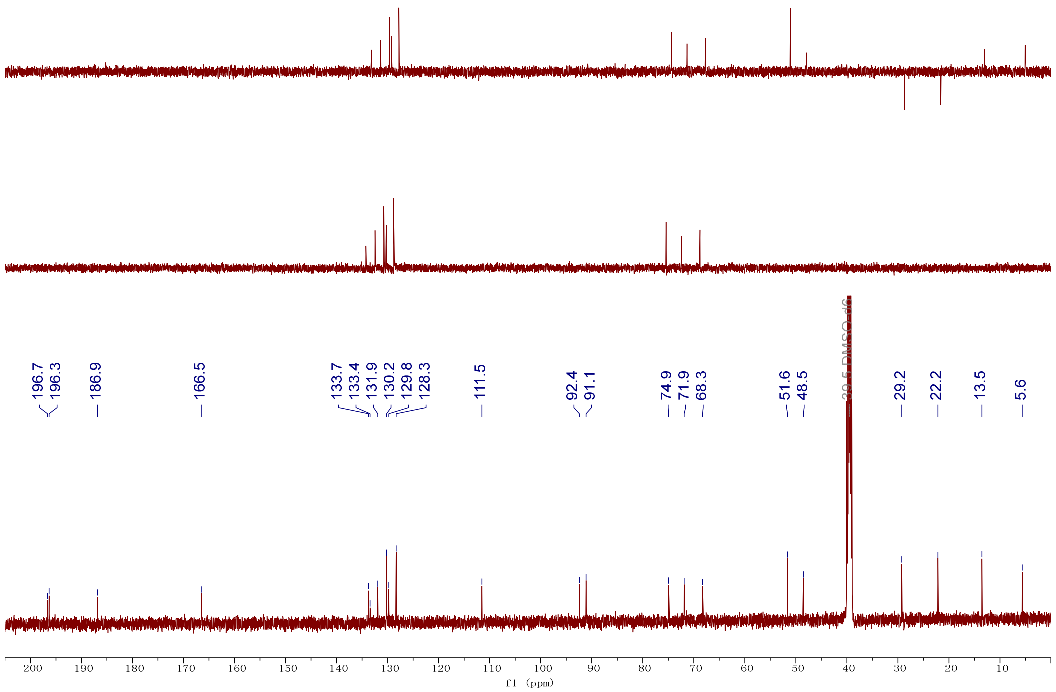

Figure S21. ^{1}H–^{1}H COSY spectrum of compound **7.**

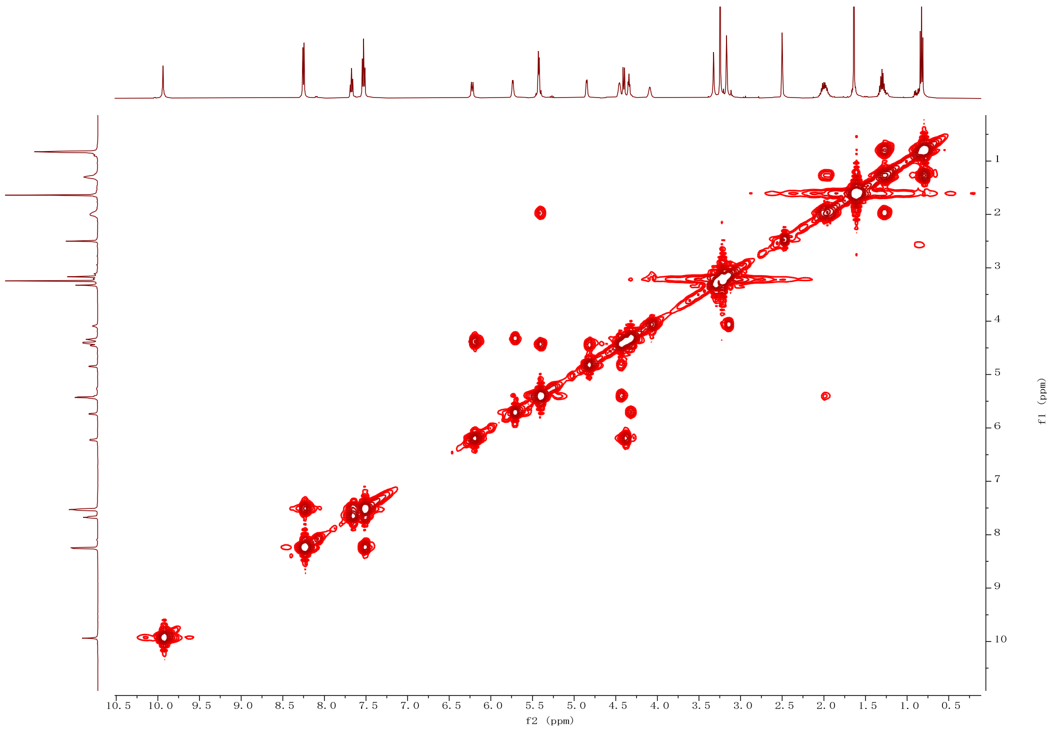

Figure S22. HSQC spectrum of compound **7.**

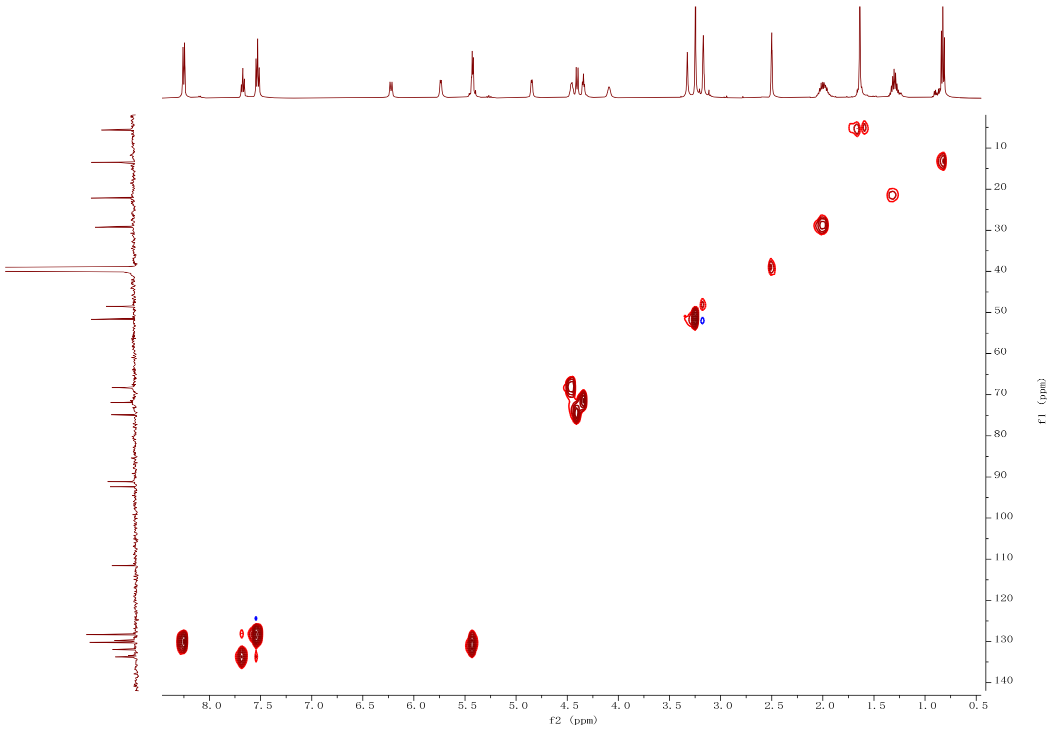

Figure S23. HMBC spectrum of compound **7.**

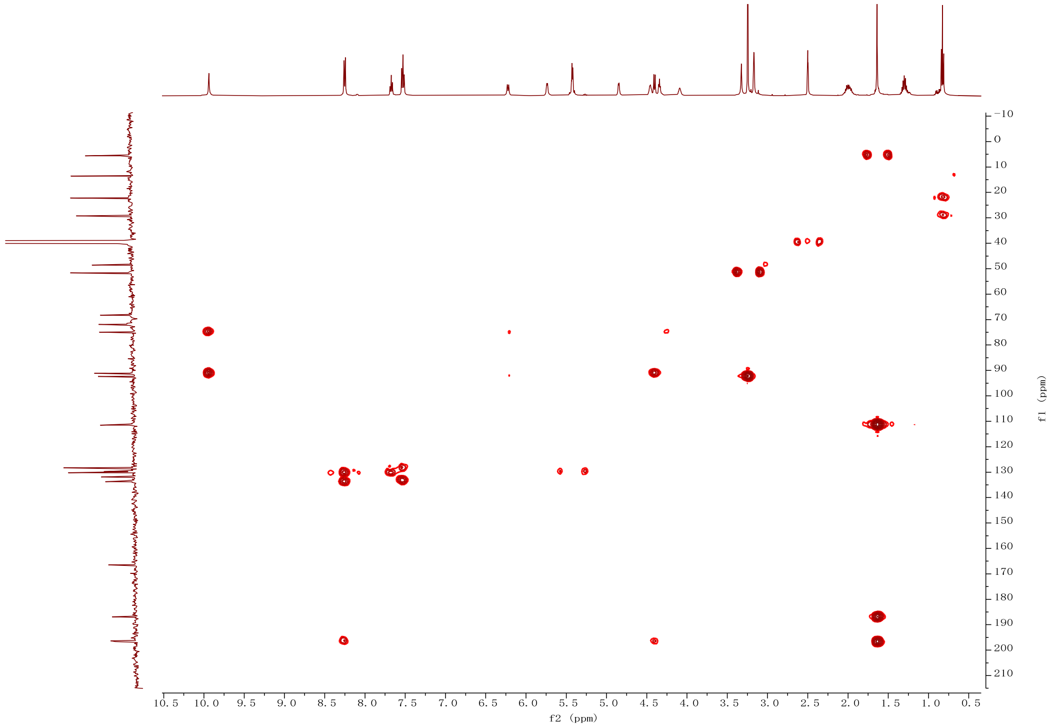

Figure S24. *J*-HMBC spectrum of compound **7.**

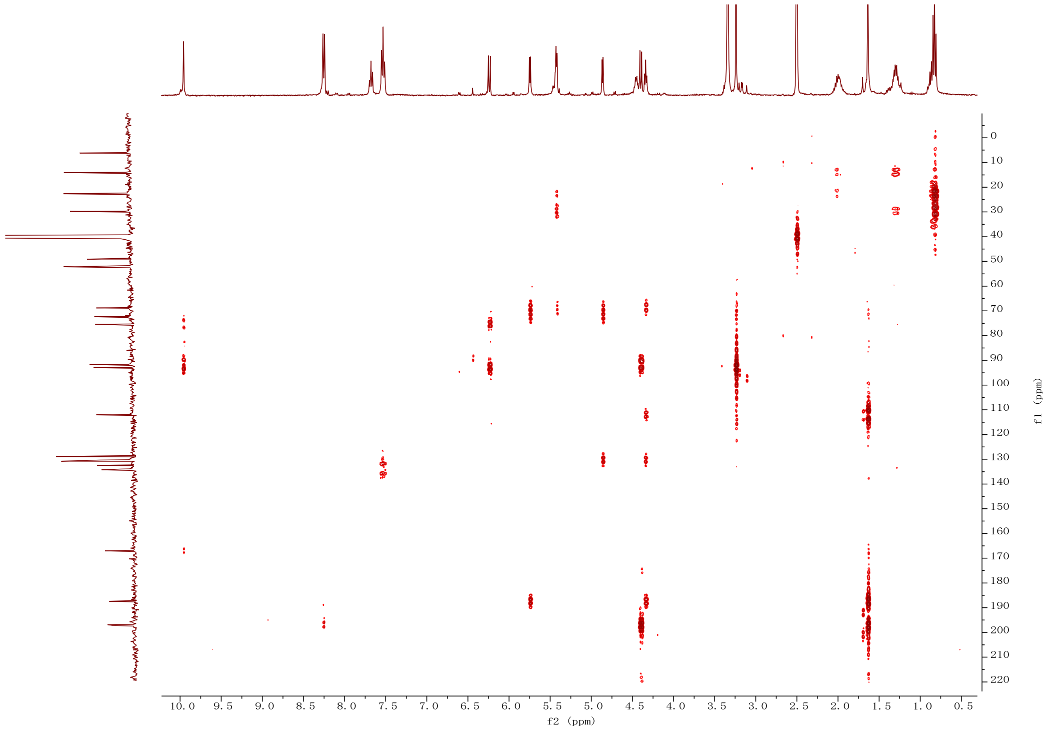

Figure S25. HR-ESI-MS spectrum of compound **7.**

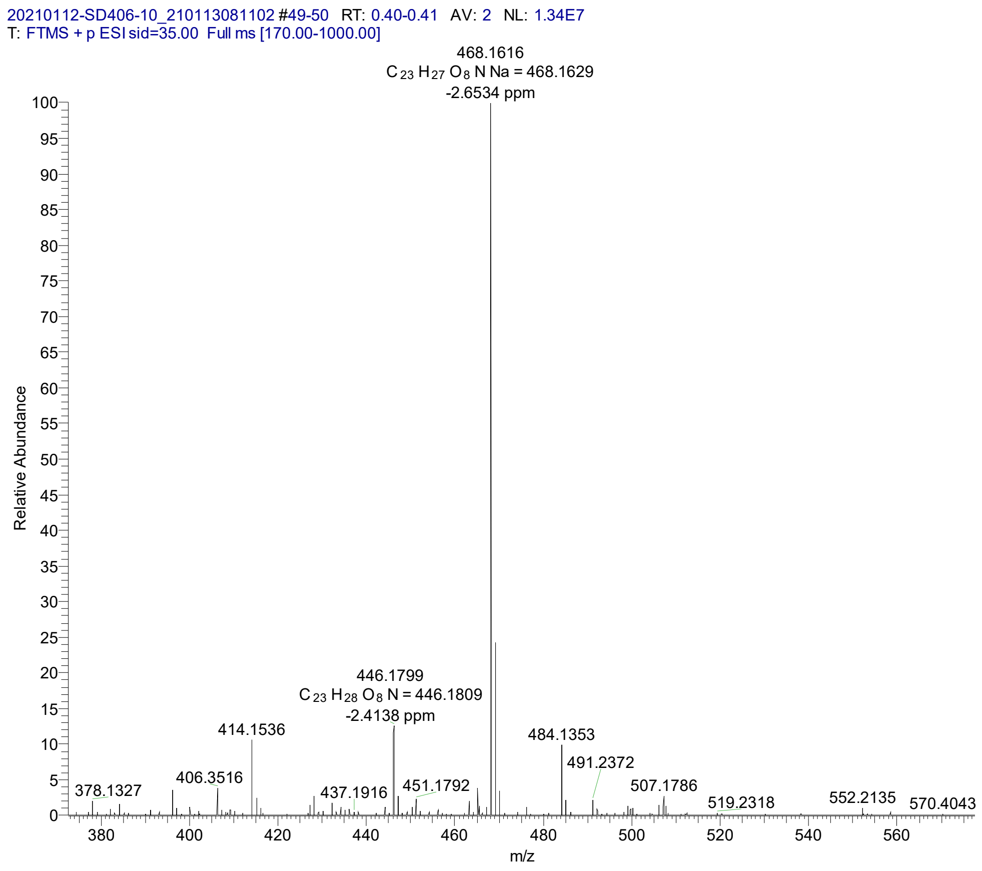

Figure S26. ECD spectrum of compound **7.**

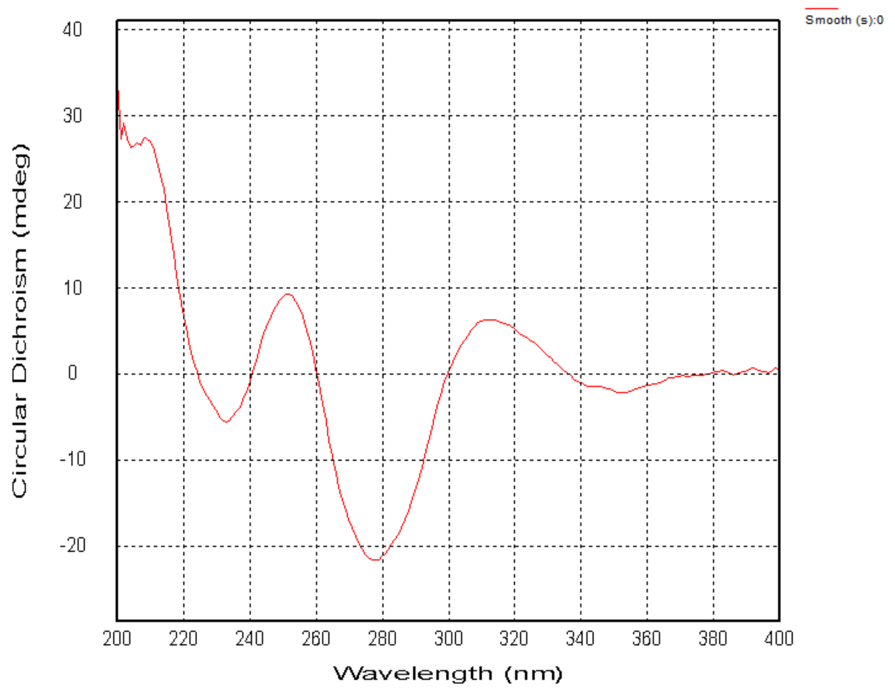

Figure S27 Optimized geometries of predominant conformers (weighting factors) for **isomers 7a-7d** at the B3LYP/6-311g(d,p) level above 2% population.

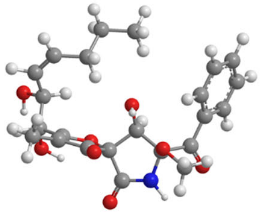

Conf. 7a-1, 33.6%

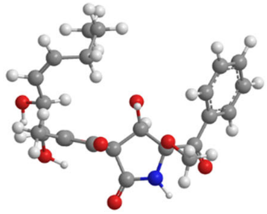

Conf. 7a-2, 30.7%

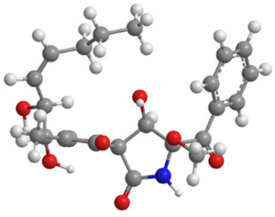

Conf. 7a-3, 12.7%

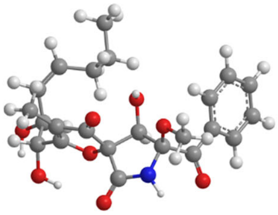

Conf. 7a-4, 6.0%

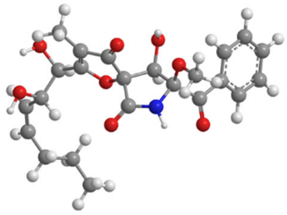

Conf. 7a-5, 3.6%

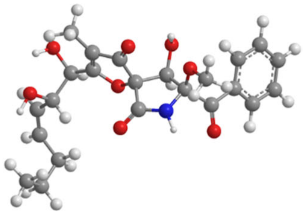

Conf. 7a-6, 3.3%

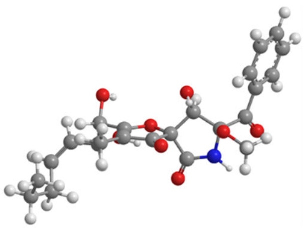

Conf. 7b-1, 58.6%

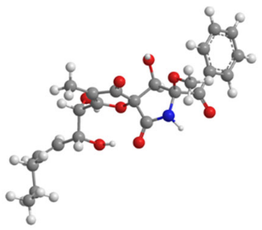

Conf. 7b-2, 24.2%

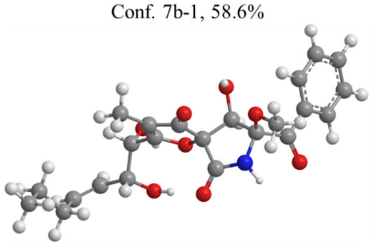

Conf. 7b-3, 9.4%

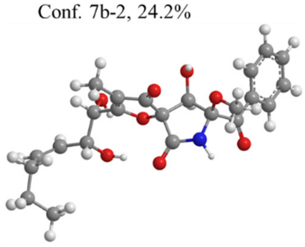

Conf. 7b-4, 7.4%

Figure S28. ^{1}H-NMR (500 MHz, DMSO-d_6) spectrum of compound **10**.

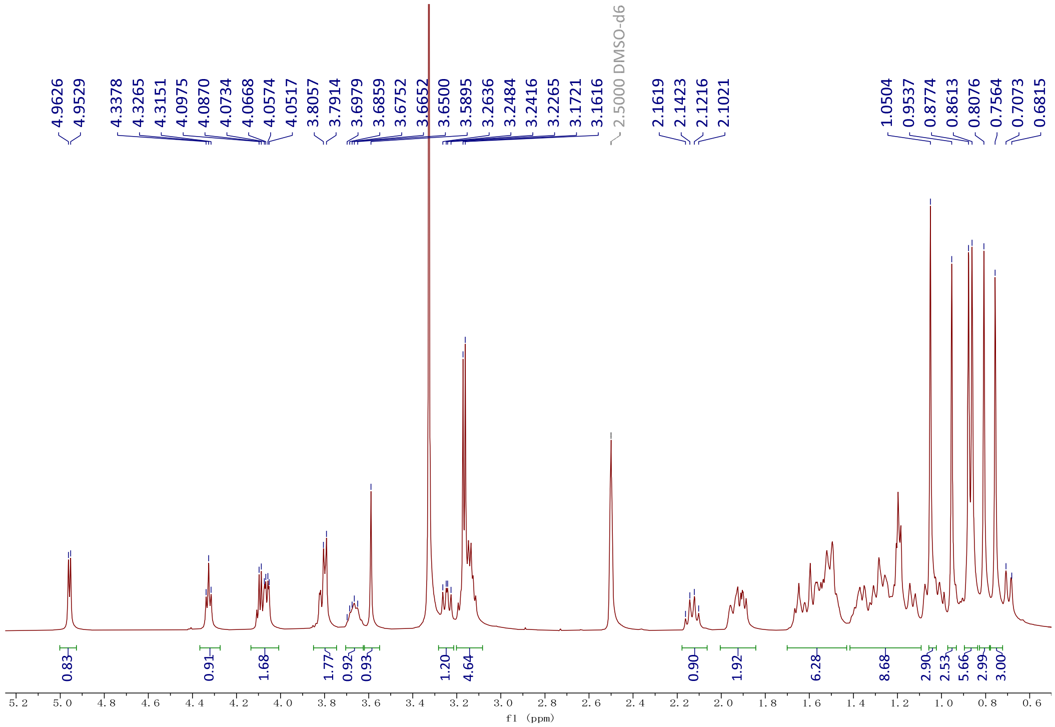

Figure S29. ^{13}C NMR (125 MHz, DMSO- d_6) and DEPT spectra of compound **10**.

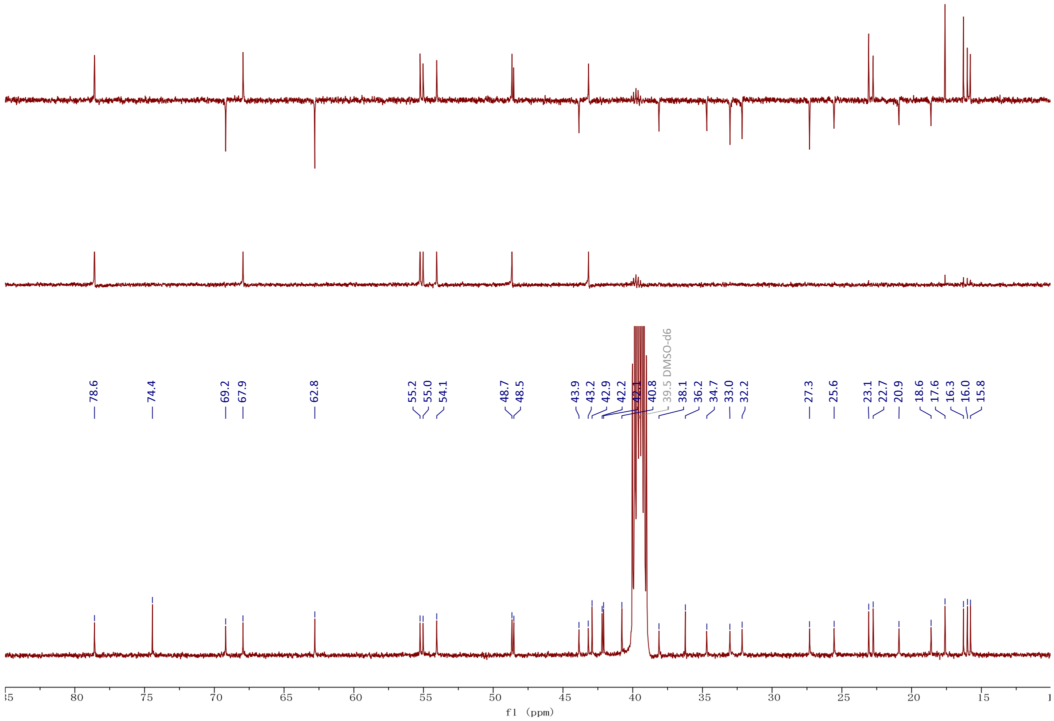

Figure S30. ^1H–^1H COSY spectrum of compound **10**.

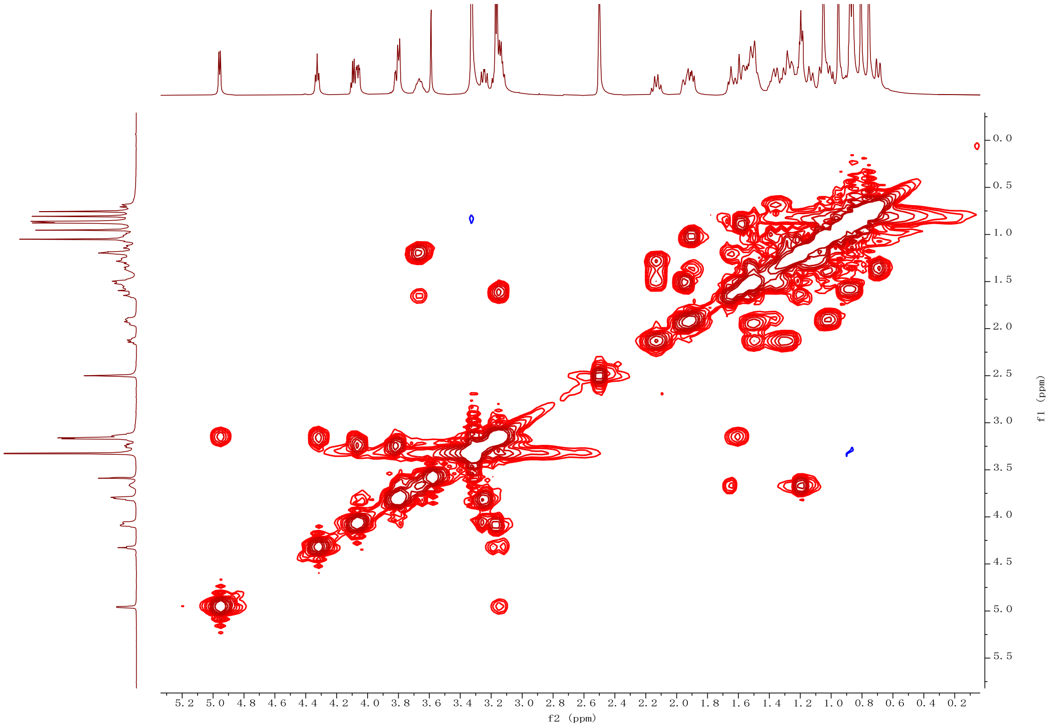

Figure S31. HSQC spectrum of compound **10**.

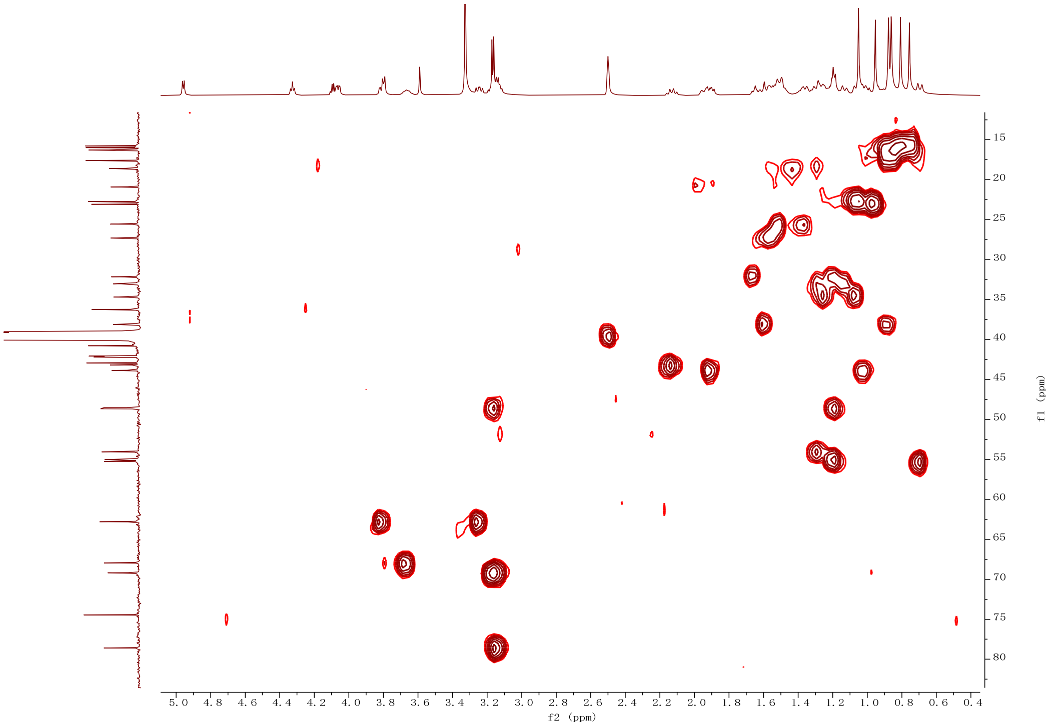

Figure S32. HMBC spectrum of compound **10**.

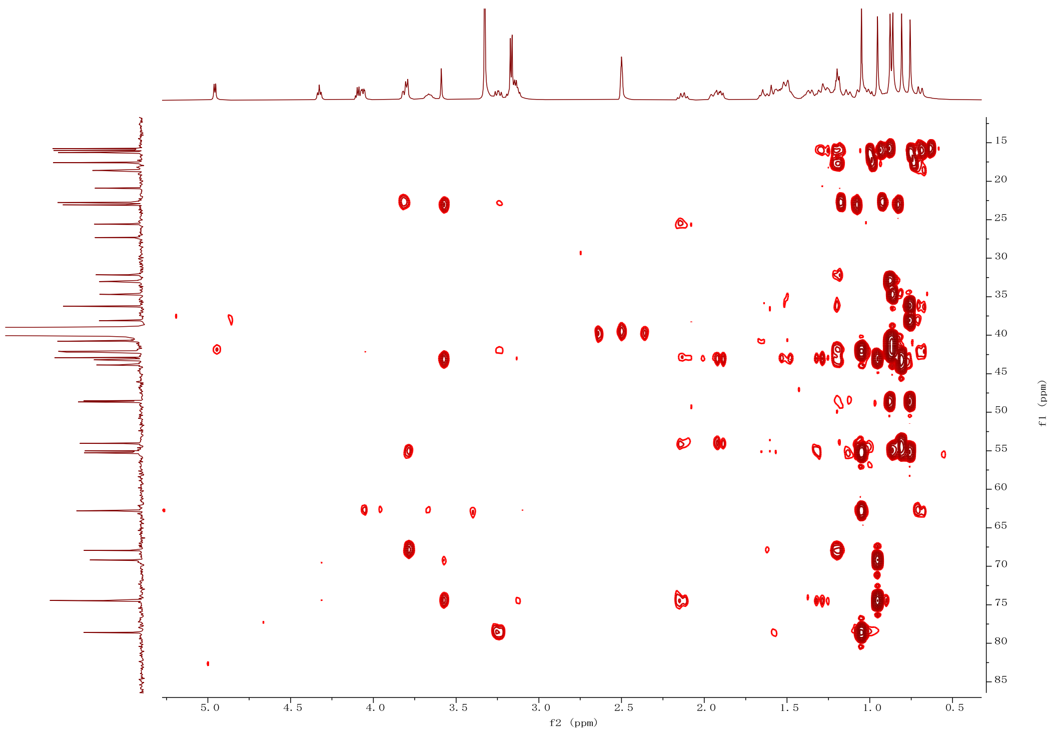

Figure S33. NOESY spectrum of compound **10**.

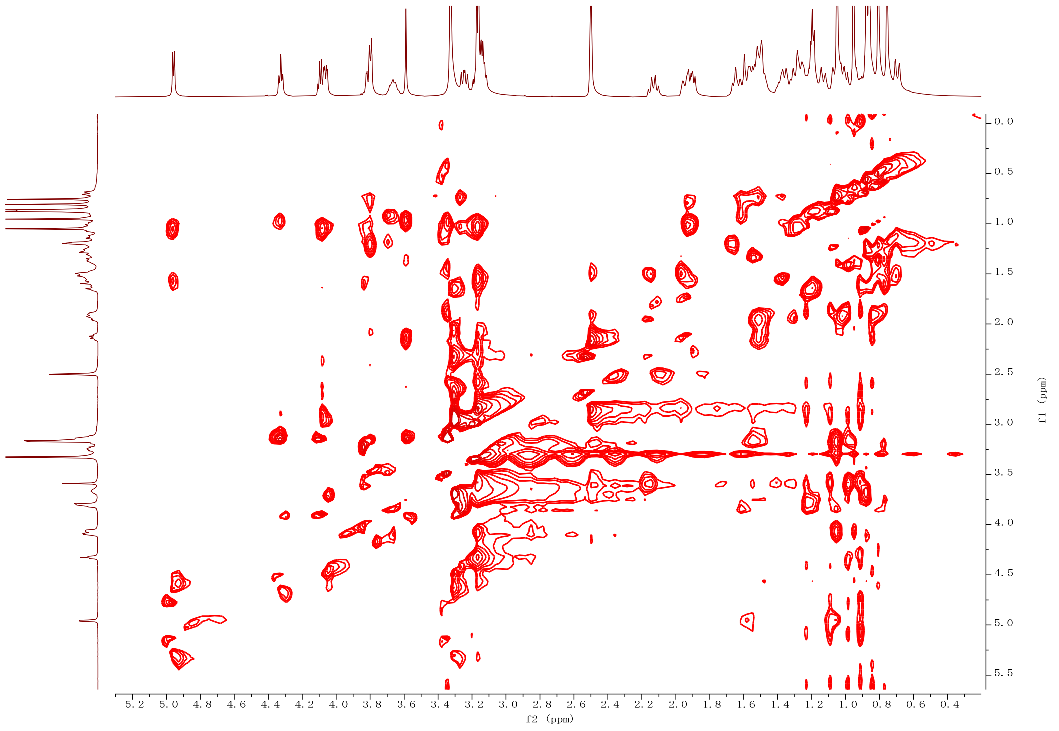

Figure S34. HR-ESI-MS spectrum of compound **10**.

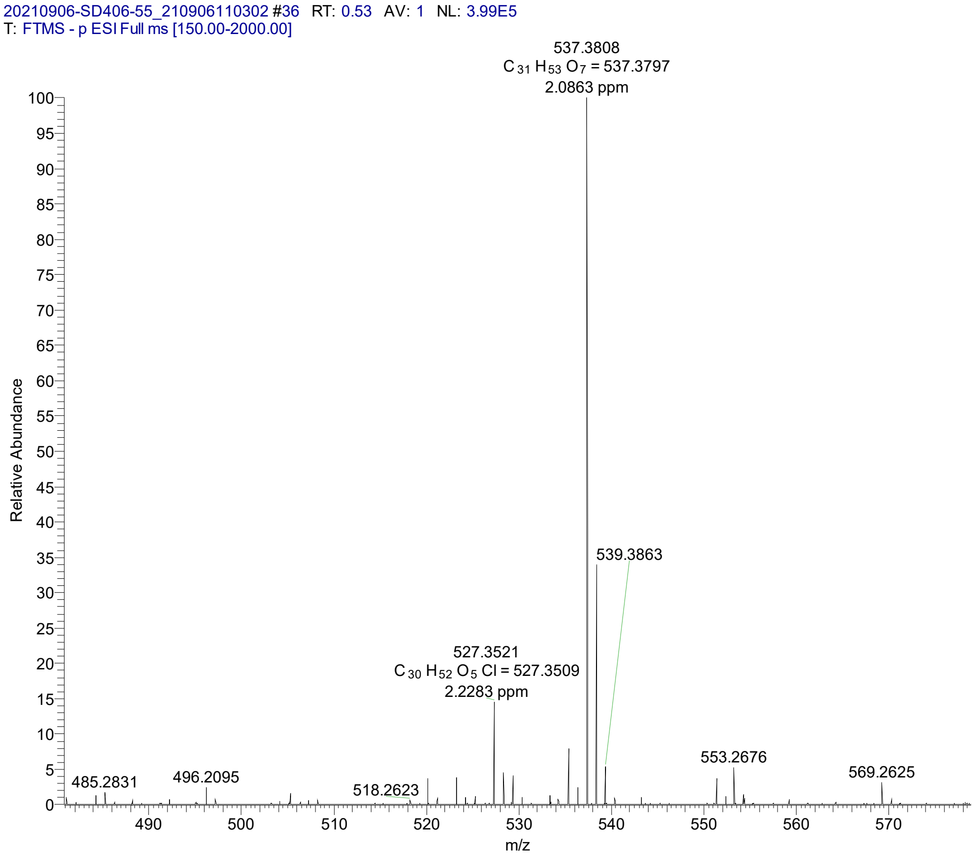

Figure S35. ^{1}H-NMR (500 MHz, DMSO-*d*6) spectrum of compound **11**.

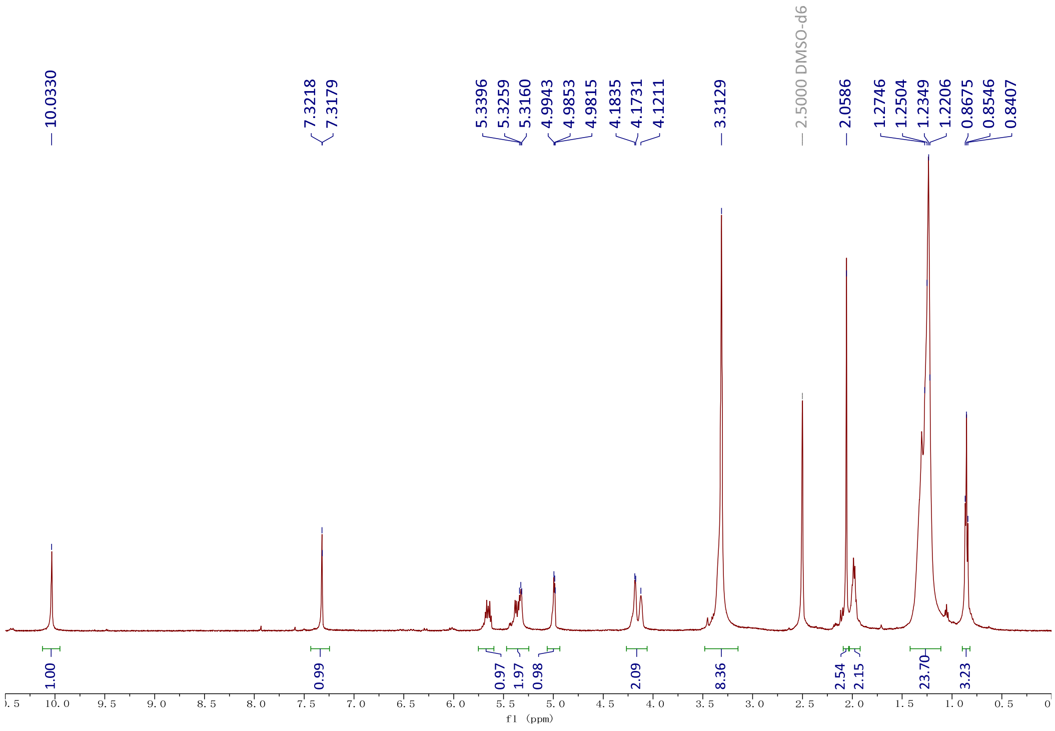

Figure S36. ^{13}C NMR (125 MHz, DMSO-d_6) and DEPT spectra of compound **11**.

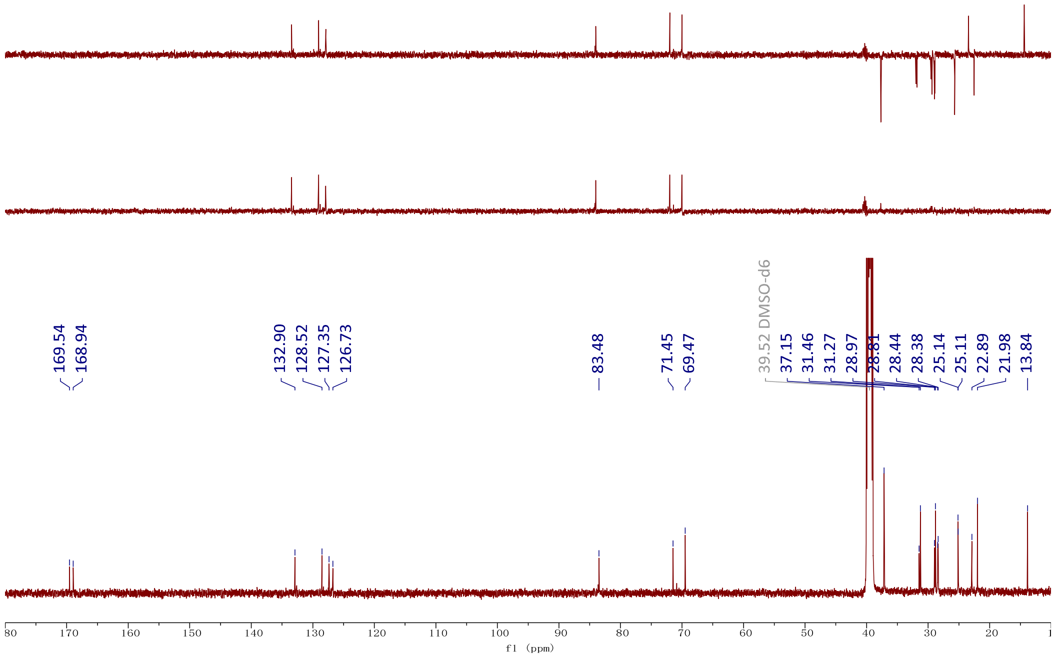

Figure S37. ^{1}H–^{1}H COSY spectrum of compound **11**.

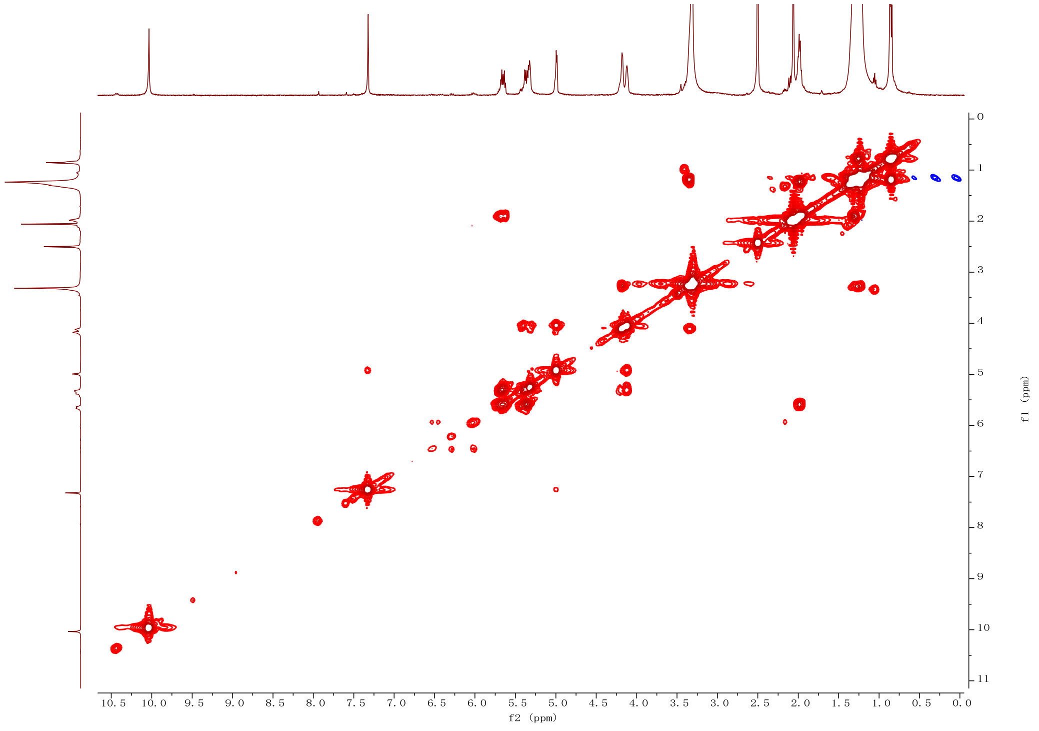

Figure S38 HSQC spectrum of compound **11**.

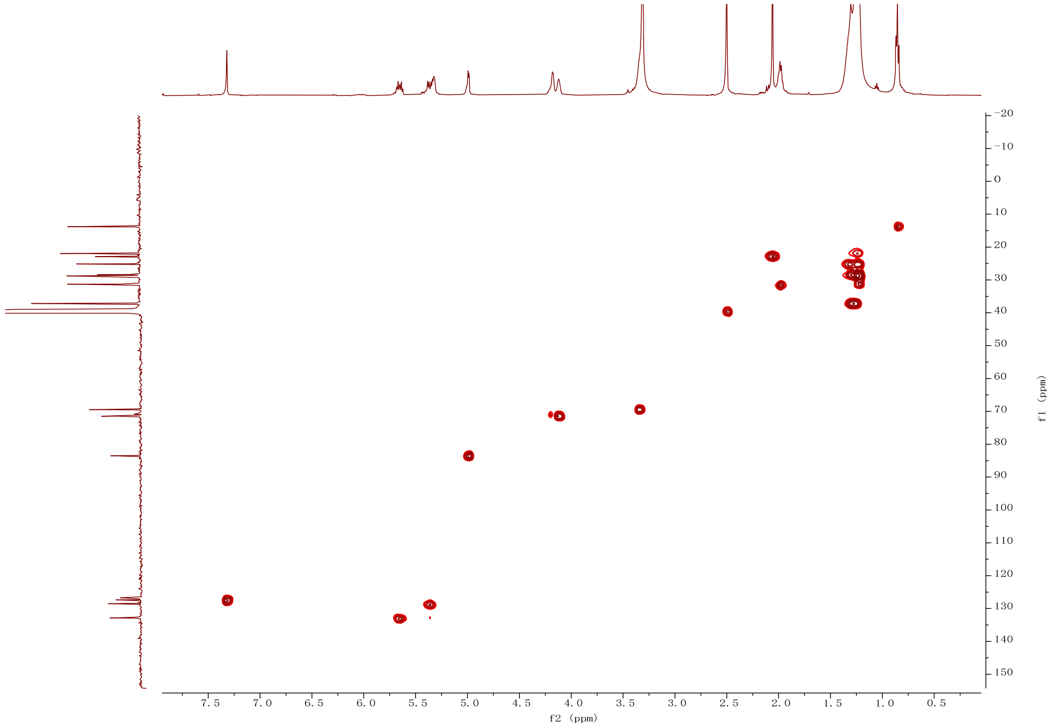

Figure S39. HMBC spectrum of compound **11**.

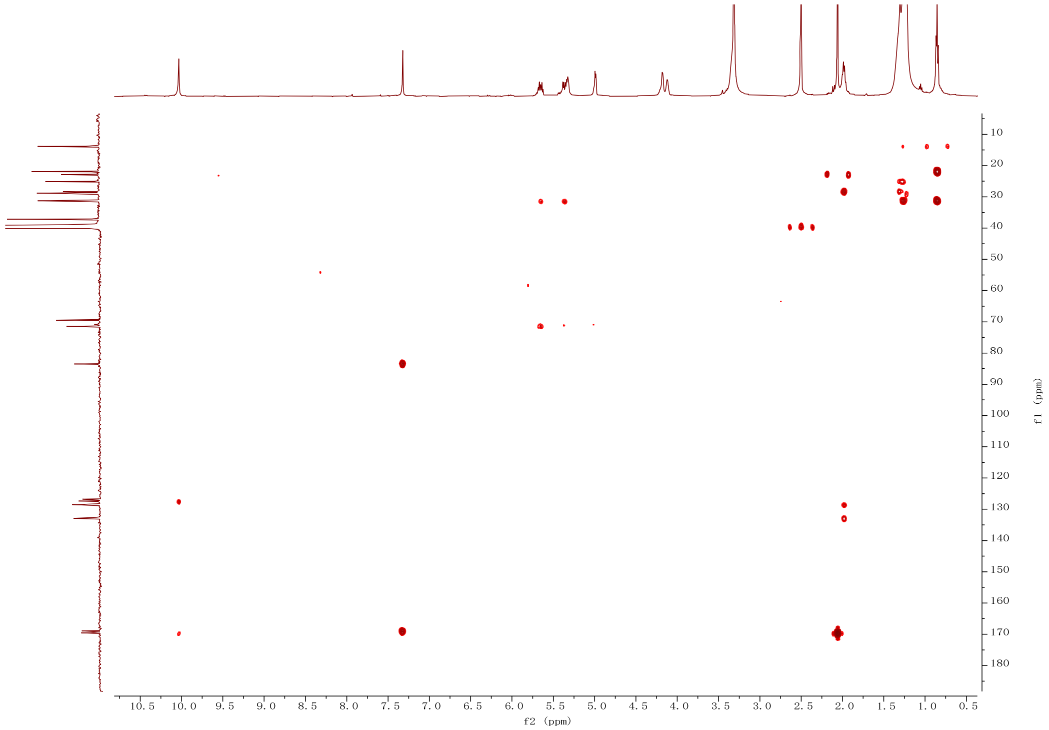

Figure S40. HR-ESI-MS spectrum of compound **11**.

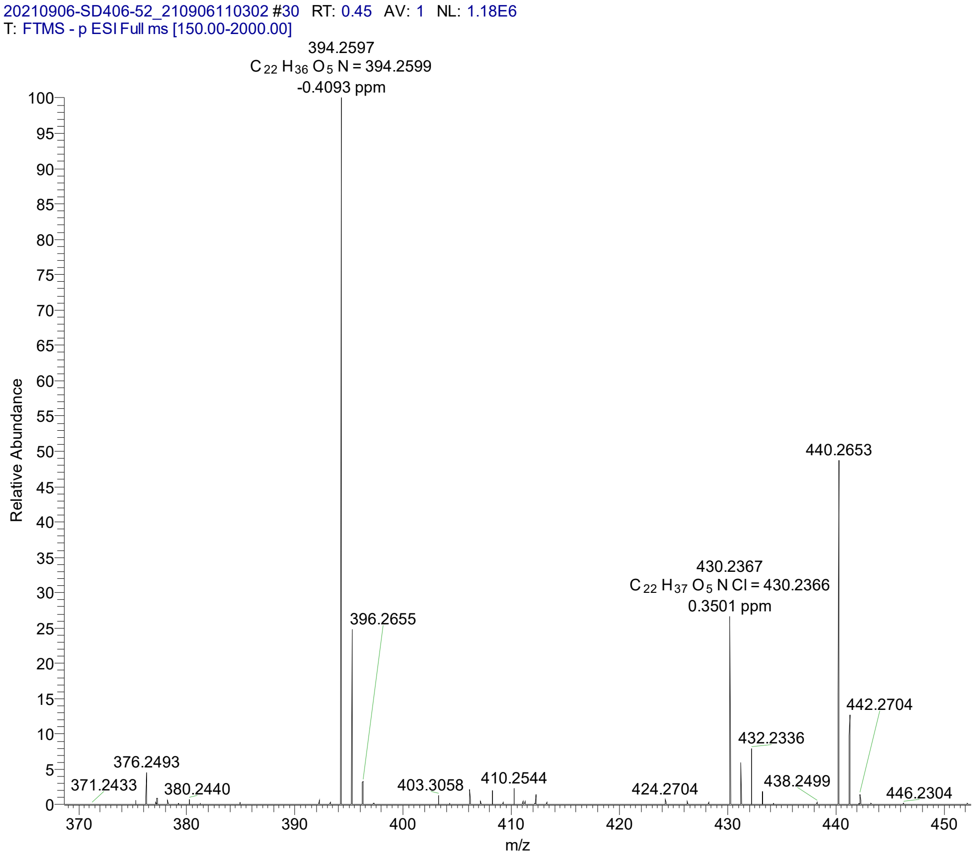

Figure S41. ECD spectrum of compound **11**.

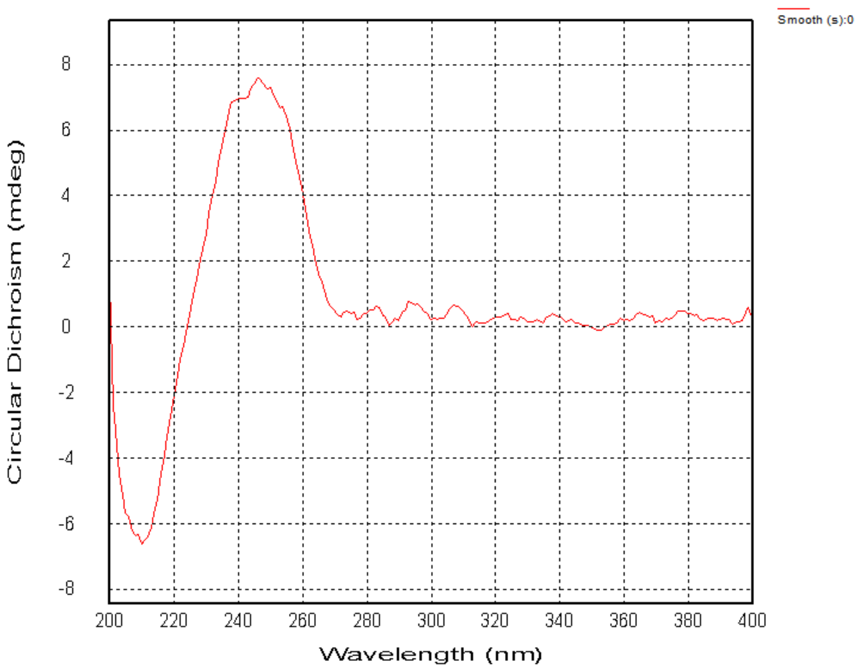

Supplement: Supplementary file 1 [file marinedrugs-20-00004-s001.zip › marinedrugs-1511161-supplementary/marinedrugs-1511161-supplementary-R3.pdf]
